# A large, consistent plasma proteomics data set from prospectively collected breast cancer patient and healthy volunteer samples

**DOI:** 10.1186/1479-5876-9-80

**Published:** 2011-05-27

**Authors:** Catherine P Riley, Xiang Zhang, Harikrishna Nakshatri, Bryan Schneider, Fred E Regnier, Jiri Adamec, Charles Buck

**Affiliations:** 1 Bindley Bioscience Center, Purdue University, West Lafayette, IN, USA; 2 Department of Chemistry, University of Louisville, Louisville, KY, USA; 3 Department of Surgery, Indiana University School of Medicine, Indianapolis, IN, USA; 4 Department of Medicine, Indiana University School of Medicine, Indianapolis, IN, USA

## Abstract

**Background:**

Variability of plasma sample collection and of proteomics technology platforms has been detrimental to generation of large proteomic profile datasets from human biospecimens.

**Methods:**

We carried out a clinical trial-like protocol to standardize collection of plasma from 204 healthy and 216 breast cancer patient volunteers. The breast cancer patients provided follow up samples at 3 month intervals. We generated proteomics profiles from these samples with a stable and reproducible platform for differential proteomics that employs a highly consistent nanofabricated ChipCube™ chromatography system for peptide detection and quantification with fast, single dimension mass spectrometry (LC-MS). Protein identification is achieved with subsequent LC-MS/MS analysis employing the same ChipCube™ chromatography system.

**Results:**

With this consistent platform, over 800 LC-MS plasma proteomic profiles from prospectively collected samples of 420 individuals were obtained. Using a web-based data analysis pipeline for LC-MS profiling data, analyses of all peptide peaks from these plasma LC-MS profiles reveals an average coefficient of variability of less than 15%. Protein identification of peptide peaks of interest has been achieved with subsequent LC-MS/MS analyses and by referring to a spectral library created from about 150 discrete LC-MS/MS runs. Verification of peptide quantity and identity is demonstrated with several Multiple Reaction Monitoring analyses. These plasma proteomic profiles are publicly available through ProteomeCommons.

**Conclusion:**

From a large prospective cohort of healthy and breast cancer patient volunteers and using a nano-fabricated chromatography system, a consistent LC-MS proteomics dataset has been generated that includes more than 800 discrete human plasma profiles. This large proteomics dataset provides an important resource in support of breast cancer biomarker discovery and validation efforts.

## Background

Proteomic analyses of readily accessible bodily fluids present a powerful opportunity to monitor experimental and control (e.g., healthy and disease) phenotypes with an extremely data-rich readout [1-3]. The proteomic approach enables detection and quantification of protein expression. Another distinct advantage of this technology is that measurement of functional gene products (i.e., proteins) may directly reflect mechanisms that differentiate groups. For example, altered expression of a cytokine protein in diseased samples can indicate signaling pathways impacted by this cytokine that may contribute to the disease process. The fact that proteomics approaches assess many hundreds and even thousands of proteins simultaneously, can also support the functional evaluation of a specific protein by revealing changes in other proteins in relevant and associated pathways. When applied in readily accessible human biofluids, such as plasma, this technology is especially promising for identification of protein biomarkers for disease diagnosis, progression, and for therapeutic efficacy [4-6].

Liquid chromatography coupled with two-dimensional mass spectrometry (LC-MS/MS) is the most commonly employed technology for proteomics [7-9]. Tryptic digestion of protein mixtures creates peptide fragments of suitable size for ionization to enable mass spectrometry analyses. High performance liquid chromatography (HPLC) is included to separate peptide mixtures according to the physical properties of the molecules and this separation of the peptides enables detection of larger numbers of peptide ions in the MS. Peptide ions are identified by dissociation within the mass spectrometer in the second MS dimension to obtain amino acid sequences that may be assigned to parent proteins via database search [10-12]. In this data-dependent 2^nd ^MS dimension identification step, the activity of the mass spectrometer is intermittently co-opted; additional peptide ion detection does not occur in this phase of the process. The second dimension MS step is typically undertaken during profiling to ensure that identified peptides are identical to the ions detected and quantified at a specific point in the same experiment [13]. Although effective, this approach introduces bias by occupying the duty cycle of the instrument for peptide ion selection and identification, rather than detection and quantification. Peptide ions originating from low abundance proteins or those with low ionization efficiency may not be selected for identification, even though some of these peptides/proteins may actually contribute to disease development. Nevertheless, this method is widely employed because variability of chromatography complicates the alternative approach of sequential, non-coupled LC-MS/MS for peptide (and protein) identification.

Proteomics technology has not yet provided validated biomarkers [14]. One reason for this is that many of the required steps suffer from a high degree of variability, particularly the chromatography component. In addition, the protocols for LC and MS require optimization of the specific technology platform (i.e., the instruments). Because of the complexity of these instruments, this process is often unique to the laboratory, not standardized, and poorly reproducible between laboratories. Although concerted efforts are underway to improve the reproducibility of targeted proteomic analyses in complex biofluids [15-17], relatively few consistent and reproducible proteomics profiling platforms have been reported. Notably, the generation of large numbers of comparable proteomic profiles from complex biofluids that will enable a data-driven evaluation of this technology on a larger scale (i.e., 'omics scale) has not been described.

The source of material for proteomic analyses is a particularly important consideration. For example, with cancer indications, it has been suggested that tumor tissue *per se *should be collected and proteins from this tissue be assessed with proteomic methods [18]. Both the availability and choice of control tissue is a significant and potentially confounding issue. Normal tissue may be difficult or impossible to obtain from living donors under conditions similar to those used for collection of tumor material. In addition, because of tumor heterogeneity, the choice of tissue to best represent the proteome of the tumor is not straightforward and will be difficult to standardize at different clinical sites. An alternative approach utilizes more readily accessible and available biological sample material such as urine or blood. Proteomic analyses of such fluids should indicate tumor proteins shed or excreted by the tumor that could be diagnostic for the presence of the tumor. These same proteins may also be useful targets for therapeutic intervention. In the case of blood plasma, such analyses are complicated by abundant proteins that comprise a disproportionate fraction of the total protein pool [19-22]. Regardless of what tissue or fluid is selected, an important goal is to standardize tissue/fluid collection in order to minimize variability in the proteomic profile that may arise from conditions of collection or storage of the biosamples.

We describe a highly reproducible proteomics platform that employs a commercially-available, nanofabricated liquid chromatography apparatus and single dimension ion trap mass spectrometry for LC-MS peptide profiling (detection and quantification). The profiling step is followed by separate LC-MS/MS analyses for protein identification with the identical, coupled LC-MS platform (Agilent ChipCube™ and XCT II ion trap mass spectrometer) [23,24]. The platform provides consistent peptide profiles with respect to quantity and quality of peptides detected in the same sample over time, including from different tryptic digestions and with different operators of the equipment [25,26]. To provide further evidence validating this platform, we report the generation of over 800 LC-MS proteomic profiles from human plasma samples that were prospectively collected and stored under standard operating procedures in a clinical trial-like protocol. Samples from healthy volunteers and breast cancer patient volunteers are included. Follow up samples from the breast cancer patient volunteers at 3 month intervals are also included. Consistency of these data is illustrated with multiple peptide peaks detected across the complex chromatograms. Follow on analyses of selected samples with LC-MS/MS provides protein identification for a high percentage of detected LC-MS peaks and enabled creation of a spectral library for human plasma. Identified proteins agree substantially with previous high confidence plasma proteomic analyses [27].

Validation of quantitative features of detected peptide peaks is further demonstrated for discrete peaks of high, medium and low abundance proteins with targeted multiple reaction monitoring (MRM) analyses [17]. For these studies, targeted analyses of plasma samples were performed on a triple quadrupole mass spectrometer employing the same ChipCube™ chromatography apparatus. The study protocol for sample collection from breast cancer patient volunteers included follow up samples from each patient at three month intervals. Proteomic profiles from hundreds of these follow up samples have been generated to enable evaluation of disease progression and therapeutic efficacy. To our knowledge this is the largest LC-MS proteomics dataset generated to date. We expect this dataset to be of substantial value for biomarker discovery and verification.

## Methods

### Trypsin digestion

Two hundred four healthy and 216 breast cancer plasma samples (100 μg per sample) were denatured with 8 M urea and 10 mM dithiothreitol (DTT) for 1.5 h at 37°C. The mixtures were subjected to reduction and alkylation with 0.5% triethylphosphine (TEP), 2% 2-Iodoethanol and 97.5% acetonitrile for 1.5 h at 37°C [28]. Samples were dried down, resuspended and digested in 100 mM ammonium bicarbonate containing 2 μg of trypsin and incubated at 37°C for 16 h. Finally, 2.5 μl of 10% trifluoroacetic acid (TFA) was added to stop the digestion. Additional discrete plasma samples collected from the breast cancer patient volunteers at 3 month intervals after study enrollment were prepared in the same fashion (followed up to 30 months). All chemicals, solvents and buffers were from Fischer Scientific (Pittsburgh, PA).

### NanoLC-Chip-MS

Plasma tryptic peptides (1 μg) were separated on a nanoLC-Chip system (1100 Series LC equipped with HPLC Chip interface, Agilent Technologies, Santa Clara, CA) [25]. The peptides were concentrated on the Agilent 300SB-C18 enrichment column and washed with 5% acetonitrile (ACN); 0.01% TFA at flow rate 3 μl/min for 5 min. The enrichment column was switched into the nano-flow path and peptides were separated with the C18 reversed phase ZORBAX 300SB-C18 analytical column (0.75 μm × 150 mm; Agilent) coupled to the electrospray ionization (ESI) source of the ion trap mass spectrometer (XCT II Plus, Agilent Technologies). The column was eluted with a 55 min linear gradient from 5% - 35% of a buffer containing 100% ACN, 0.01% TFA at a rate of 300 nl/min, followed by a 10 min gradient from 35% - 100%. The column was equilibrated with an isocratic flow (5% of same buffer) at 300 μl/min. The system was controlled by Agilent ChemStation software. NanoLC-MS chromatograms were acquired in positive ion mode. Acquisition range was 350 - 2000 m/z with 0.15 s maximum accumulation time and scan speed of 8,100 m/z per second.

### NanoLC-Chip-MS/MS and targeted MS/MS

Trypsin digested human healthy and breast cancer plasma peptides were separated on a nanoLC-Chip system using the same setup and gradient as described above. Automated MS/MS spectra were acquired during the run in the data-dependent acquisition mode with the selection of the three most abundant precursor ions (0.5 min active exclusion; 2+ ions preferred). These spectra were used to generate a plasma spectral library for the project. Targeted MS/MS spectra were acquired during the run in the data-dependent acquisition mode for specific masses associated with the peaks of interest when required for protein identification.

### Protein Identification

NanoLC-Chip-MS/MS spectra were analyzed using Spectrum Mill A.03.02.060 software (Agilent Technologies) and searches were performed against the human IPI database (International Protein Index, version 3.03). The parameters of the search were as follows; no more than two tryptic miscleavages allowed, cysteine searched as iodoethanol, 1.0 Da peptide mass tolerance and 0.7 Da fragment ion mass tolerance [29].

### Merging MS and MS/MS data

A peak list was generated from alignment of 204 healthy and 216 baseline breast cancer samples analyzed with LC-MS, and from 97 and 49 of these analyzed with LC-MS/MS, respectively. The raw data from the MS and MS/MS files were compared to ensure that the molecular information [m/z (+/- 0.7 Da), retention time (+/- 0.5 min), charge state] and chromatographic patterns were the same in each file. The lists were combined to provide a project peak list.

### Multiple Reaction Monitoring (MRM) analysis

MRM analysis was performed using the same Agilent nanoLC-chip system coupled to a triple quadruple tandem mass spectrometer (6410 series, Agilent Technologies) using the same column and gradient as described above. NanoLC-MS/MS chromatograms for three of the peptides identified using targeted MS/MS were acquired in positive ion mode under the following conditions: capillary voltage of 1950 V; dry temperature of 300°C; and dry gas flow of 4 l/min. Other acquisition parameters and the chromatographic retention times of the peptide compounds measured are listed in Table 1. Data acquisition and analysis were accomplished using MassHunter software (version B 2.0.1, Agilent Technologies).

**Table 1:** Proteins, peptides and transitions selected from LC-MS/MS spectra and the corresponding parameters for MRM verification of plasma expression levels.

*Proteins*	*Peptides*	Transitions precursor ion [M+H]*-> product ion	*Retention time (min)*	*dwell time (min)*	*Fragmentor energy (kV)*	*Collision energy (kV)*
ApoA1	DYVSQFEGSALGK	701.1->532.4	34	100	200	20
						
		701.1->661.4				25
						
		701.1->808.5				30

Hemopexin	EVGTPHGIILDSVDAAFICPGSSR	829.8>650.3	44	100	200	25
						
		829.8>992.3				30
						
		829.8>909.7				27

Angiotensin preprotein	ADSQAQLLLSTVVGVFTAPGLHLK	822.8->664.4	62	100	200	20
						
		822.8->877.1				25
						
		822.8->816.7				22

### Plasma sample collection

All samples were obtained from volunteers by healthcare professionals under defined standard operating procedures in a clinical trial-like protocol undertaken by the Hoosier Oncology Group, a not-for-profit project partner organization. All volunteers were enrolled following informed consent and in compliance with the health insurance portability and accountability act (HIPAA) and with authorization for release of personal health information (PHI). Inclusion criteria for the breast cancer cohort were: age ≥18 years, female (not pregnant), histologically/cytologically confirmed invasive disease or ductal carcinoma *in situ *(DCIS), preparing to begin a new therapeutic regimen. For the healthy control cohort inclusion criteria were: age ≥18 years, females (not pregnant), no history of invasive breast cancer or DCIS, no history of malignancy in past 5 years (with the exceptions of basal/squamous cell cancer with low potential for metastasis). Plasma sample processing was initiated within 30 min of blood draw to an ethylenediaminetetraacetic acid (EDTA) containing tube. Samples were spun for 30 min at 3500 rpm in a clinical centrifuge. Plasma was immediately harvested in approximately 1 ml aliquots and frozen at either -20°C or -80°C. Frozen samples were shipped by overnight courier to the Hoosier Oncology Group laboratory for storage at -80°C until use.

### Data Analysis and Statistics

The Proteome Discovery Pipeline (PDP) bioinformatics infrastructure created at the Bindley Bioscience Center at Purdue University was used for data management and data analyses [30]. Briefly, the pipeline converted the raw data into mzXML format using Bruker's CompassXport program and then processed the data files with Xmass and Xalign software for deconvolution and alignment [31,32]. A log linear model was used for peptide peak normalization across samples [33]. The parametric student's t test was employed for statistical evaluation of peptide peak expression levels between groups. Normalized values were employed to calculate the percentage coeffient of variance (CV) [34]. For LC-MS/MS peptide identification, only peptides with a Spectrum Mill score of 5 or higher and Spectrum Mill Scored Peak Intensity (SPI) of 70% or higher were considered positives [29]. Three specific and discrete transitions and their intensities were monitored for each peptide in the MRM analyses to ensure accuracy [15,35].

## Results

A stable proteomic profiling platform is required for proteomic analyses of plasma samples donated by healthy volunteers and breast cancer patients. We collected samples in a clinical trial-like protocol as part of an NCI-sponsored clinical proteomics technology assessment for cancer (CPTAC) biomarkers project. All plasma samples were specifically collected for proteomics analyses under standard operating procedures. A rapid data collection ion trap instrument was selected for profiling (Agilent XCT II Plus) coupled with HPLC via the nanofabricated Agilent ChipCube™ chromatography column for improved reproducibility and high resolution via a highly stable nano-flow rate (18 μl/h).

**Figure 1:**
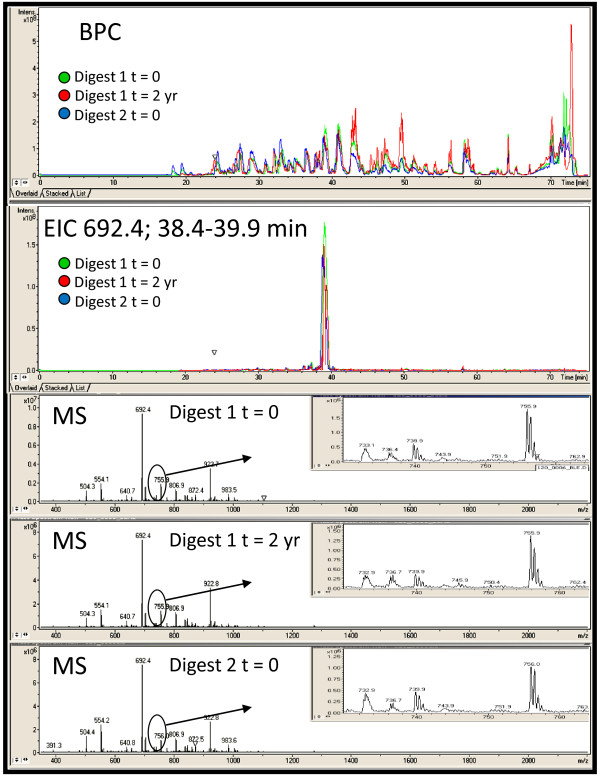
**Base peak (BPC) and extracted ion chromatographs (EIC; mass over charge (m/z) value of 692.4) from one healthy plasma sample analyzed on three different dates using the LC-MS platform**. Both the overall BPC and randomly selected EIC are consistently represented in the sample over time and between tryptic digests. The green chromatographs are from the original sample digest (10/27/2008) run on the day of the tryptic digestion, red traces are from the same sample digest stored at -80°C for 22 months (run on 8/30/2010), and the blue traces are from a new tryptic digest of the same plasma sample (digested and run on 8/31/2010). The corresponding MS scans illustrate summed spectra (RT 38.4-39.9 minutes) associated with the major peak from each of the EICs. Insets indicate similarity even for a very low intensity region of the spectra.

Proteomic analyses run on the same platform at different times have been reported to exhibit high variability on multiple proteomic platforms [1,36]. We assessed variability of our platform over time and with different technical operators (Figure 1). The same plasma sample digest analyzed two years apart showed good reproducibility with the sample stored at -80°C in the interval between runs (CV = 2.4%). Similarly, proteomic profiles of different tryptic digests, and a sample run two years apart, are reproducible (CV = 4.3%). These analyses were also run by two different operators. Similar consistency is observed throughout the life of the ChipCube™ column and between different columns and column batches. Additionally, as can be seen in the base peak chromatogram (BPC) overlays in Figure 1, there is more variability in these hydrophobic peptides eluted off the column after 40 min, compared to the peptides eluted off the column earlier. The consistency of the platform is further illustrated with a randomly selected ion from these single plasma sample analyses, illustrated by the extracted ion chromatographs (EIC; Figure 1). This low intensity peak is detected with excellent reproducibility between different tryptic digests and with analyses separated by two years. The sources of technical variability of the analytical platform, including plasma storage, protein digestion, chromatography, and data processing must all be separately controlled.

The consistency of the platform across multiple samples was assessed with samples from 10 individuals in each of two groups. The average CV of all peptide peak areas detected in plasma samples from 10 discrete healthy volunteers is 7.6% and 9.2% for 10 discrete breast cancer patient volunteer plasma samples. All 10 of the breast cancer patients selected for this group were diagnosed with stage I disease. The proteomics profiling platform showed good consistency between samples within the same group (healthy volunteers and breast cancer patient volunteers). Variations between biological samples confound the accuracy of the proteomics analyses. However, intra-group CVs of less than 10% for LC-MS proteomic profiles that simultaneously measure hundreds of proteins is excellent.

The behavior of the ChipCube™ chromatography column was assessed with multiple columns and samples. The total number of detected peptide peaks from 420 discrete plasma sample LC-MS proteomic profiles, including samples run over a span of two years with different nanofabricated columns, averages 2348 peaks with an average CV of 14.4%. Additionally, when these samples are aligned with our data analysis pipeline [30], 92% of all peaks aligned, indicating the stability of the profiling platform. The aligned peak intensities range from 7,844 to 53,400,700. The detected peaks are derived from proteins in all abundance classes (Additional file 1, Table S1).

**Figure 2:**
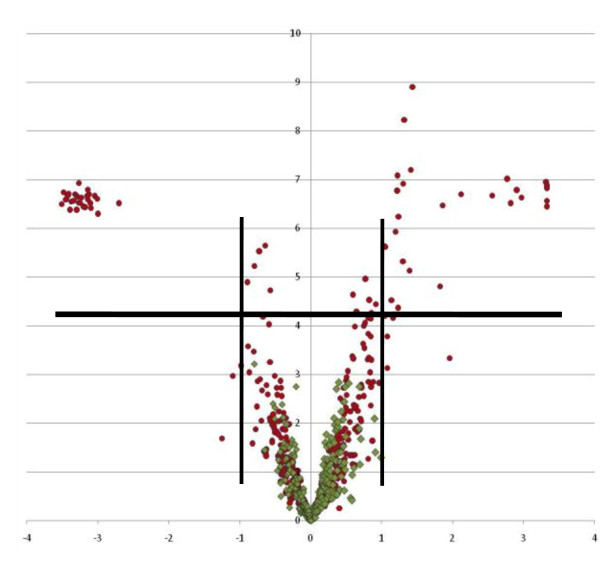
**Statistical evaluation of LC-MS peptide peak expression level differences**. Volcano plot displaying intensity differences of peaks from LC-MS proteomic profiles of 10 replicate injections of a single plasma sample (green). The same analyses of the intensity differences of peaks from a comparison of LC-MS profiles of healthy volunteer and breast cancer patient volunteer plasma samples is also displayed (red balls, 20 discrete plasma samples in each group). The negative log2 scale is displayed for each axis: horizontal and vertical lines indicate fold change greater than 2 and p values < 0.05.

A primary goal for differential proteomics is to detect those proteins that are significantly differently expressed between groups. To evaluate the likelihood of false discovery with our platform, we have compared LC-MS profiles on replicates of individual samples that would not be expected to provide significant differences in peptide peak intensity. Figure 2 shows the statistical evaluation of replicate injections of the same plasma sample. For comparison, the same statistical evaluation performed on LC-MS profiles from 20 healthy volunteer plasma compared with 20 breast cancer patient plasma samples is also included. The self-comparison does not result in peptides recognized as differentially expressed (no statistically different peaks are identified). In contrast, many peaks differentially expressed between these healthy volunteer and baseline breast cancer patient volunteers are identified (71 peaks with p value of < 0.05 and a fold change of 2 or higher). Candidate biomarkers from our very large dataset will be described elsewhere (Riley et al., in preparation).

While the LC-MS proteomics profiling platform offers several advantages, this approach does not include identification of proteins. This is a critical aspect of the proteomics workflow that enables assessment of the involvement of specific proteins in relevant processes and pathways. Because of the consistency of the ChipCube™ apparatus, identical conditions were employed to perform LC-MS/MS analyses of a group of the same plasma samples (including both healthy and baseline breast cancer patient volunteers) to obtain protein identification for peptide peaks of interest. Thus in our platform, specific peaks of interest (e.g., those differentially expressed between groups) may be targeted for LC-MS/MS analyses for peptide identification. In addition, we have completed full spectrum LC-MS/MS experiments on nearly 150 discrete human plasma samples to create an LC-MS/MS spectral library for these human plasma samples. Peptide peaks of interest may be identified directly from this spectral library without the requirement to re-run a sample in LC-MS/MS mode and to target a specific peptide mass and retention time. This same LC-MS/MS platform may be employed to target specific peptide peaks of interest for identification. In addition, the MS/MS spectral information can be employed to identify specific peptides of interest for follow-on, independent verification studies with sensitive and quantitative multiple reaction monitoring (MRM) studies on a triple quadrupole mass spectrometer employing the same nanochromatography unit (see below). The LC-MS/MS data from these plasma samples was submitted to protein database search algorithms to identify the proteins. We routinely employ the Spectrum Mill™ data search algorithm but other search algorithms can also be used to analyze the LC-MS/MS data for protein identification (e.g., X!tandem, Sequest, Mascot) [10-12,29]. Proteins identified are listed in Additional file 1, Table S1.

As expected, abundant plasma proteins are well represented in the database search results from the LC-MS/MS data. However, in 146 LC-MS/MS experiments, a total of 1351 discrete proteins were identified with high confidence. A manually-validated, high confidence, mass spectrometry protein data set generated from 11 human plasma samples depleted of abundant plasma proteins and containing 697 proteins, was recently described [27]. Our results confidently identify 306 of the proteins in this plasma protein reference set (44%). This indicates that protein identification with our methods provide coverage of the plasma proteome that is consistent with existing high confidence plasma proteome analyses and that our platform is not overwhelmed with detection of abundant plasma proteins.

**Figure 3:**
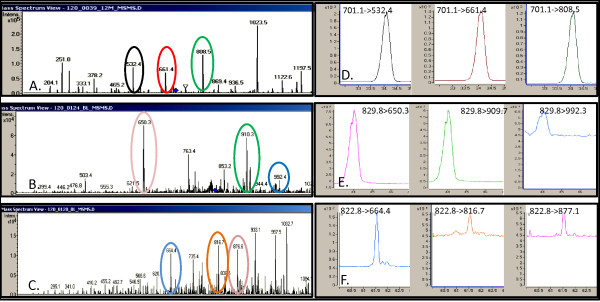
**Representative MRM analyses of three selected plasma proteins**. The proteins evaluated are ApoA1 (A, D); Hemopexin (B, E), and Angiotensin preprotein (C, F). Panels A-C illustrate LC-MS/MS scans from the spectral library used to develop the MRM. The transitions in the original MS/MS scan are indicated with the colored ovals matching the targeted MRM peaks in panels D-F that show each MRM transition and the relative intensity of each transition.

We employed multiple reaction monitoring (MRM) of peptide peaks in the triple quadrupole mass spectrometer to assess the consistency of our proteomics profiling platform and to obtain independent verification of the LC-MS-derived detection and the LC-MS/MS protein identification data that it provides [17]. We employ for these studies the Agilent 6410 triple quadrupole mass spec equipped with the ChipCube™ accessory to standardize chromatography; in this case, between the ion trap and triple quadrupole mass spectrometers. To confirm the consistency of the LC-MS profiling platform on a peak-by-peak basis, we arbitrarily selected specific LC-MS peptide peaks of high, medium and low intensities for MRM analyses in 10 plasma samples from the healthy volunteer group (Table 1). In each sample, the independent and targeted MRM analysis confirms the identity of these three peptides detected with LC-MS profiling and identified by LC-MS/MS (Figure 3). These independent analyses provide additional support for the consistency of our LCMS proteomic profiling platform. The relative plasma concentrations we detected by LC-MS for these proteins is consistent with other reports [37-39].

**Figure 4:**
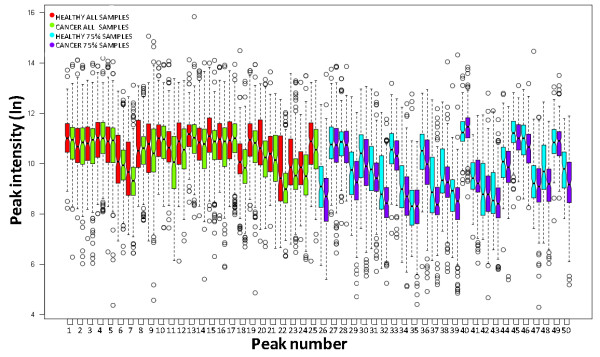
**Intensity distribution of plasma LC-MS peptide peaks**. Example intensity distributions are shown for 25 randomly selected LC-MS peaks found in each of 420 plasma samples (peaks 1-25, red and green boxes) and 25 randomly selected peaks found in at least 75% of all plasma samples (peaks 26-50, blue and purple boxes). The dark center line in each box represents the median intensity for each peak and the surrounding box contains the interquatrile (+/- 25%) of the data points for that peak. The whiskers show peaks with intensities up to two standard deviations from the median; circles represent peak intensities from these 420 plasma samples that are outside of this range.

To exploit the consistency of our LC-MS proteomic platform, we generated profiles from a very large collection of human plasma samples prospectively collected in our CPTAC program clinical trial-like protocol. The samples were obtained under institutional review board (IRB)-approved informed consent from healthy volunteers and volunteer breast cancer patients scheduled to begin a new treatment regime (here designated as 'baseline' samples). These patients also provided samples at each 3 month follow up visit with their oncologist. These time course samples were obtained to enable studies of therapeutic efficacy and disease progression. As was the case with the small sample sets, the consistency of profiles from this large number of plasma samples was excellent. To illustrate the performance of the LC-MS platform at this scale of analysis, we selected random peptide peaks that were detected in both the healthy volunteer and baseline breast cancer patient volunteer data sets. There were 79 and 68 peaks detected in every healthy (n = 204) and every breast cancer baseline plasma (n = 216) sample, respectively. A total of 50 peaks were detected in every one of these 420 plasma samples. In the breast cancer patient sample set, the average CV for each common peak was 9.3%. The CV for the common peaks in the healthy volunteer sample set was 10.8%. The intensity distributions of 25 of these peaks, selected at random, are illustrated in Figure 4 (red and green boxes).

We also performed the intensity distribution analysis on peaks that appear consistently in a group but not necessarily in every sample, consistent with many biomarker discovery approaches. A peak-by-peak assessment of randomly selected peaks that were detected in at least 75% of the 204 healthy and 216 breast cancer volunteer human plasma samples was performed (that is, the selected peaks were identified in greater than 150 of the plasma samples in each group). The intensity distributions across all samples of each of 25 randomly selected peaks that meet these criteria are also shown in Figure 4 (blue and purple boxes). The distribution of these peaks includes those with high, medium and low intensities. The average cv for each peak was 11.3% for the healthy volunteer sample set and 11.1% for the breast cancer patient sample set. The consistent LC-MS proteomics profiling platform is again demonstrated. Analysis with criteria for inclusion of peptide peaks that are not detected in every sample still provides quantitative detection of peaks with acceptable coefficients of variation. Furthermore, employing the 75% inclusion criteria, as for a biomarker discovery analysis, facilitates comparison of peak intensities between groups. Peaks with different intensities that reach statistical significance may be considered candidate biomarkers that warrant identification and additional evaluation.

## Discussion

As a result of widely appreciated difficulties with reproducibility of proteomic profiling, large datasets that will provide a richer molecular description of protein content in biosamples have not been reported. Although gel free LC-MS-based global proteomics has introduced remarkable speed and sensitivity for biomarker discovery [1-3], high technical variability has severely limited the use and impact of these approaches. Isotope labeling strategies have been developed to improve the reliability of LC-MS results [40-43]. Additionally, the advantages of ultra high performance LC-MS instruments such as Fourier transform ion cyclotron resonance (FT-ICR) MS have been extensively explored [27,44]. Unfortunately, the impact of these strategies is limited by the high costs for reagents and instruments and the associated need for in-depth technical expertise [45,46]. Nevertheless, highly reproducible proteomic technology platforms and protocols hold great promise for biomarker discovery. In addition, consistent data collected from large numbers of high quality samples will enable development of advanced informatic approaches to more effectively utilize proteomic data to classify experimental groups and patient populations.

Proteomic profiling of complex biosamples with LC-MS, rather than the more commonly employed data dependent LC-MS/MS approach, presents several advantages. First, the LC-MS approach enables more thorough collection of data in the mass spectrometer since the duty cycle of the instrument is not occupied with collecting the second MS information during profiling [47-49]. The cost per sample is also decreased with shorter sample run times. Second, generation and capture of more complete data from across the chromatographic spectrum provides a solution to the problem of biasing the results with peptides from abundant proteins and undersampling of complex mixtures. Since the instrument is less occupied with peak selection for a second MS dimension, it is more likely that less abundant and rare protein peptides will be detected in the mass spectrometer [50]. Third, quantification is simplified with area under the curve calculation for detected peaks. Fourth, inclusion of a protein identification step, which is error prone and computationally expensive, is not included in the initial proteomic detection and quantification steps of the LC-MS proteomics pipeline. In this case, the consistency of the ChipCube™ chromatography component integrated into our platform typically enables protein identification for peaks of interest directly from the human plasma LC-MS/MS spectral library we have created from MS/MS analyses of nearly 150 discrete human plasma samples; additional and subsequent targeted LC-MS/MS analysis is often not required to identify protein peaks of interest. However, peptide peaks not identified in the spectral library that correspond to proteins of interest, such as those that may differentiate sample groups, can be readily identified subsequent to the LC-MS profiling step in targeted LC-MS/MS follow-up sequencing experiments.

In the platform described here, chromatography is standardized with the nanofabricated ChipCube™ apparatus that enables strong reproducibility of peptide behaviors between samples and over time (Figure 1). The combination of the nano-flow rate and the ChipCube™ apparatus affords improved reliability with very consistent chromatography and excellent sensitivity for peptide detection with eliminated dead volumes and very low flow rates [49,51-53]. The LC-MS proteomics platform is coupled with a recently developed LC-MS data analysis pipeline to facilitate generation and analyses of large numbers of proteomic profiles from complex biological samples [30]. This developed platform has been employed to compare proteome profiles of large numbers of breast cancer patients with healthy volunteers. Proteomic profiling results with these samples on our LC-MS platform provides excellent consistency and reproducibility.

Independent verification of the accuracy of quantification derived from the LC-MS label free analysis must be preformed to improve confidence in candidate biomarker selection. An MRM analysis of additional samples is a highly sensitive and specific approach [17]. The information in our LC-MS/MS peptide spectral library can be effectively used to design MRM methods with little to no optimization. This independent verification of expression levels of specific proteins of interest can be augmented with software predictors for MRM method transition ions that avoid contaminating ions not belonging to the peptide of interest (such as Skyline; http://proteome.gs.washington.edu/software/skyline/) [54].

In addition to the 420 healthy and baseline breast cancer patient volunteer plasma samples, we completed LC-MS proteomic profiling analyses on approximately 400 follow up samples collected every three months from the breast cancer patient volunteers in our study (up to 36 months). These human plasma samples have been employed to reveal proteins that may indicate development or presence of breast cancer and to ascertain the changes in breast cancer plasma proteome with therapeutic treatment and disease progression (Riley et al., manuscript in preparation). This report provides the opportunity to make available this very large human plasma LC-MS proteomic profiles dataset that has been deposited with Tranche, a data repository of ProteomeCommons https://proteomecommons.org[55,56].

## Conclusions

A robust liquid (nano)chromatography mass spectrometer (LCMS) platform enables reproducible proteomic profiling from human plasma samples. Consistency of the platform enabled profiling of over 800 discrete human plasma samples comprising the largest human proteomic profile dataset to date. Comparison of plasma samples at the proteome scale (hundreds to thousands of proteins) will allow detection of candidate biomarkers (i.e., differentially expressed proteins). Associated LCMS/MS data from many of the same samples enables protein identification. The accuracy of LCMS proteomic profiling protein quantification and subsequent LCMS/MS identification was demonstrated with MRM using peptide transitions predicted from the platform. All of these data are available publicly for independent analysis and provide a resource for plasma protein biomarker discovery and verification.

## Competing interests

The authors declare that they have no competing interests.

## Authors' contributions

CPR carried out the experiments, performed the data analysis and contributed to writing the manuscript. XZ participated in experimental design and contributed to data analysis and preparation of the manuscript. HN and BS provided oversight for sample collection and analyses and provided clinical and cancer biology input for the manuscript. FER and JA provided technical expertise for proteomics studies. CB provided supervision for the research, performed data analysis, and wrote the manuscript. All authors read and approved the final manuscript.

## Supplementary Material

Additional file 1 **Table S1 - All protein identifications from LC-MS/MS analyses of human plasma samples**. Proteins identified with confidence using the Spectrum Mill^© ^search engine are provided as listed in the International Protein Index (IPI) database. Parameters for confidence evaluation are provided in the Methods section. Click here for file
