# Supplementary material for: A large, consistent plasma proteomics data set from prospectively collected breast cancer patient and healthy volunteer samples
Source: J Transl Med. 2011 May 27;9:80. doi: 10.1186/1479-5876-9-80 (PMC3120690; doi:10.1186/1479-5876-9-80)
Supplement: Additional file 1 — Table S1 - All protein identifications from LC-MS/MS analyses of human plasma samples. Proteins identified with confidence using the Spectrum Mill© search engine are provided as listed in the International Protein Index (IPI) database. Parameters for confidence evaluation are provided in the Methods section. [file 1479-5876-9-80-S1.PDF]

Table S1. Supplemental data for Riley et al., 2011 -- Proteins identified from LC-MS proteomics profiling platform

#	***Number of Peptides***	***Spectrum Mill Score***	***Percent Coverage***	***Database***	***Accession Number***	***Protein Name***
1	2	11.03	2	ipi.HUMAN	IPI00008787	NAGLU Alpha-N-acetylglucosaminidase precursor
2	2	10.07	15	ipi.HUMAN	IPI00010133	coronin 1A
3	2	16.09	3	ipi.HUMAN	IPI00217998	GPR115 84 kDa protein
4	2	10.1	17	ipi.HUMAN	IPI0011252	C8A Complement component C8 alpha chain precursor
5	3	10.11	1	ipi.HUMAN	IPI00006674	ABCC3 Isoform 3 of Canalicular multispecific organic anion transporter 2
6	2	11.12	5	ipi.HUMAN	IPI00166200	Gasdermin
7	2	12.12	8	ipi.HUMAN	IPI00644631	HLA class I histocompatibility antigen, alpha chain 2
8	2	10.28	3	ipi.HUMAN	IPI00792737	BRAP BRCA1-associated protein
9	2	10.34	17	ipi.HUMAN	IPI00060715	BTB/POZ domain-containing protein KCTD12
10	2	11.34	21	ipi.HUMAN	IPI00027507	Complement factor H-related protein 3 precursor
11	2	10.35	8	ipi.HUMAN	IPI00024175	PSMA7 Isoform 1 of Proteasome subunit alpha type-7
12	2	10.36	2	ipi.HUMAN	IPI00022200	type VII collagen
13	2	18.38	8	ipi.HUMAN	IPI00021772	MAT1A S-adenosylmethionine synthetase isoform type-1
14	2	10.41	13	ipi.HUMAN	IPI00009866	keratin type 1 cytoskeletal 13
15	2	10.42	2	ipi.HUMAN	IPI00023673	galectin-3 binding protein
16	2	10.44	14	ipi.HUMAN	IPI00218803	Fibulin-1 splice isoform 1
17	3	17.53	7	ipi.HUMAN	IPI00786897	LOC391508 similar to Phosphoglycerate mutase 1
18	2	10.55	6	ipi.HUMAN	IPI00004573	PIGR Polymeric immunoglobulin receptor precursor
19	2	10.56	6	ipi.HUMAN	IPI00291005	MDH1 Malate dehydrogenase, cytoplasmic
20	2	10.57	33	ipi.HUMAN	IPI00031490	collectin sub-family member 11
21	2	10.59	27	ipi.HUMAN	IPI00007917	collectin sub-family member 10
22	2	10.59	1	ipi.HUMAN	IPI00339228	Fibronectin splice isoform 8
23	2	11.66	0	ipi.HUMAN	IPI00024284	HSPG2 Basement membrane-specific heparan sulfate proteoglycan core protein precursor
24	22	10.74	6	ipi.HUMAN	IPI00000811	PSMB6 Proteasome subunit beta type-6 precursor
25	2	10.75	9	ipi.HUMAN	IPI00001610	insulin-like growth factor 1 A
26	2	11.8	79	ipi.HUMAN	IPI00005161	actin related protein 2/3 complex, subunit 2, 34kDa, isoform CRA_c

#	Number of Peptides	Spectrum Mill Score	Percent Coverage	Database	Accession Number	Protein Name
27	2	11.93	17	ipi.HUMAN	IPI00219575	bleomycin hydrolase
28	2	11.99	5	ipi.HUMAN	IPI000299150	CTSS Cathepsin s precursor
29	2	10.15	5	ipi.HUMAN	IPI00465431	galectin 3
30	2	10.3	14	ipi.HUMAN	IPI00010314	delta-aminolevulinic acid dehydratase
31	2	10.39	8	ipi.HUMAN	IPI00003351	extracellular matrix protein 1 precursor
32	2	11.39	4	ipi.HUMAN	IPI00009477	intercellular adhesion molecule 2, isoform CRA_c
33	2	11.45	15	ipi.HUMAN	IPI00019954	CST6 Cystatin-M precursor
34	3	10.58	6	ipi.HUMAN	IPI00003933	HAGH hydroxyacyl glutathione hydrolase isoform 1
35	2	9.59	4	ipi.HUMAN	IPI00296537	Fibulin-1 precursor
36	2	11.62	2	ipi.HUMAN	IPI00178440	elongation factor 1 b
37	2	10.79	3	ipi.HUMAN	IPI00644977	CFHR4 Complement factor H-related protein 4A precursor
38	3	10.83	25	ipi.HUMAN	IPI0021856	apolipoprotein C-II precursor
39	3	10.97	2	ipi.HUMAN	IPI00654888	KLKB1 Plasma kallikrein precursor
40	2	10.05	17	ipi.HUMAN	IPI00215746	fatty acid-binding protein, adipocyte
41	4	10.2	1	ipi.HUMAN	IPI00220143	MGAM Maltase-glucoamylase, intestinal
42	2	10.23	6	ipi.HUMAN	IPI00025318	SH3 domain binding glutamic acid-rich protein
43	2	10.24	3	ipi.HUMAN	IPI00219025	glutaredoxin-1
44	3	10.28	20	ipi.HUMAN	IPI00004656	B2M Beta-2-microglobulin
45	2	10.32	6	ipi.HUMAN	IPI00000513	E-cadherin
46	2	10.39	5	ipi.HUMAN	IPI00022417	leucine-rich alpha-2-glycoprotein 1
47	2	10.51	65	ipi.HUMAN	IPI00166768	TUBA6 protein
48	3	10.58	22	ipi.HUMAN	IPI00216393	Clathrin light chain A
49	2	10.62	14	ipi.HUMAN	IPI00219757	glutathione S-transferase P
50	2	10.73	4	ipi.HUMAN	IPI00008494	intercellular adhesion molecule 1 precursor
51	3	10.74	3	ipi.HUMAN	IPI00011229	CTSD Cathepsin D precursor
52	2	10.81	10	ipi.HUMAN	IPI00027350	peroxiredoxin 2, isoform CRA_c
53	2	10.88	13	ipi.HUMAN	IPI00219953	CMPK1 cytidine monophosphate (UMP-CMP) kinase 1, cytosolic
54	2	10.93	11	ipi.HUMAN	IPI00218728	PAFAH1B1 Isoform 1 of Platelet-activating factor acetylhydrolase IB subunit alpha

Table S1. Supplemental data for Riley et al., 2011 -- Proteins identified from LC-MS proteomics profiling platform

#	Number of Peptides	Spectrum Mill Score	Percent Coverage	Database	Accession Number	Protein Name
55	2	11.22	7	ipi.HUMAN	IPI00410323	TXNDC2 Isoform 1 of Thioredoxin domain-containing protein 2
56	2	11.25	3	ipi.HUMAN	IPI00027235	ATRN Isoform 1 of Attractin precursor
57	2	11.25	18	ipi.HUMAN	IPI00027255	MYL6
58	2	11.25	28	ipi.HUMAN	IPI00004798	cysteine-rich secretory protein
59	3	11.3	2	ipi.HUMAN	IPI00872555	CFI Complement factor I precursor
60	3	11.32	8	ipi.HUMAN	IPI00028147	angiotensin I converting enzyme
61	2	11.33	2	ipi.HUMAN	IPI00291807	CPAMD8 C3 and PZP-like, alpha-2-macroglobulin domain containing 8
62	2	11.37	23	ipi.HUMAN	IPI00032325	CSTA Cystatin-A
63	2	11.43	20	ipi.HUMAN	IPI00007425	desmocollin 1 isoform Dsc1b preproprotein
64	2	11.46	14	ipi.HUMAN	IPI00219299	aspartate aminotransferase
65	2	11.5	9	ipi.HUMAN	IPI00025426	pregnancy zone protein
66	2	11.54	5	ipi.HUMAN	IPI00019090	COL19A1 Collagen alpha-1
67	2	11.61	8	ipi.HUMAN	IPI00747707	KRT17 Radiated keratinocyte mRNA 266
68	2	11.63	6	ipi.HUMAN	IPI00041065	HGF activator like protein
69	2	11.64	3	ipi.HUMAN	IPI00220351	ITGB3 Isoform Beta-3C of Integrin beta-3 precursor
70	2	11.69	16	ipi.HUMAN	IPI00221224	aminopeptidase N
71	2	11.77	7	ipi.HUMAN	IPI00019038	lysozyme C
72	2	11.8	5	ipi.HUMAN	IPI00646007	PDILT Protein disulfide isomerase-like protein of the testis
73	2	11.8	2	ipi.HUMAN	IPI00159322	TCF20 Isoform 1 of Transcription factor 20
74	2	11.8	3	ipi.HUMAN	IPI00477992	Complement C1q subcomponent, B chain
75	2	11.81	10	ipi.HUMAN	IPI00216134	tropomyosin 1 alpha chain, splice isoform 2
76	2	11.88	5	ipi.HUMAN	IPI00019884	ACTN2 Alpha-actinin-2
77	2	11.88	7	ipi.HUMAN	IPI00465436	CAT Catalase
78	2	11.9	4	ipi.HUMAN	IPI00156171	ENPP2 Ectonucleotide pyrophosphatase/phosphodiesterase family member 2
79	2	11.97	1	ipi.HUMAN	IPI00289819	IGF2R Cation-independent mannose-6-phosphate receptor precursor
80	2	11.97	10	ipi.HUMAN	IPI00296099	thrombomodulin-1 precursor

#	Number of Peptides	Spectrum Mill Score	Percent Coverage	Database	Accession Number	Protein Name
81	2	12	36	ipi.HUMAN	IPI00005162	actin related protein 2/3 complex subunit 3
82	2	12.08	25	ipi.HUMAN	IPI00106687	Latexin
83	2	12.13	10	ipi.HUMAN	IPI00022418	Fibronectin splice isoform 7
84	2	12.15	4	ipi.HUMAN	IPI00010697	ITGA6 Integrin alpha-6 precursor
85	2	12.18	4	ipi.HUMAN	IPI00470535	CACNA2D1 Dihydropyridine receptor alpha 2 subunit
86	2	12.2	5	ipi.HUMAN	IPI0024046	CDH13 Cadherin-13 precursor
87	2	12.23	25	ipi.HUMAN	IPI00555956	PSMB4 Proteasome subunit beta type-4 precursor
88	2	12.29	20	ipi.HUMAN	IPI00164755	alpha 2 type 1 collagen
89	2	12.34	3	ipi.HUMAN	IPI00024825	PRG4 Isoform A of Proteoglycan-4 precursor
90	2	12.35	11	ipi.HUMAN	IPI00013219	ILK Integrin-linked protein kinase
91	2	12.41	4	ipi.HUMAN	IPI00217236	TBCD Isoform 4 of Tubulin-specific chaperone A
92	2	12.53	11	ipi.HUMAN	IPI00016768	LDHAL6B L-lactate dehydrogenase A-like 6B
93	2	12.56	17	ipi.HUMAN	IPI00290077	KRT15 Keratin, type I cytoskeletal 15
94	2	12.57	12	ipi.HUMAN	IPI00246975	GSTM3 Glutathione S-transferase Mu 3
95	2	12.63	5	ipi.HUMAN	IPI00013744	ITGA2 Integrin alpha-2 precursor
96	2	12.64	10	ipi.HUMAN	IPI00218474	beta-enolase
97	2	12.65	13	ipi.HUMAN	IPI00290462	CBR3 Carbonyl reductase [NADPH] 3
98	2	12.67	5	ipi.HUMAN	IPI00744685	BTD biotinidase precursor
99	2	12.7	27	ipi.HUMAN	IPI00514806	S100A2 S100 calcium binding protein A2
100	2	12.74	27	ipi.HUMAN	IPI00387169	PCSK9 Isoform 2 of Proprotein convertase subtilisin/kexin type 9 precursor
101	2	12.75	11	ipi.HUMAN	IPI00013179	PTGES2 Prostaglandin-H2 D-isomerase precursor
102	2	12.79	9	ipi.HUMAN	IPI00061200	KRT71 Keratin, type II cytoskeletal 71
103	2	12.79	6	ipi.HUMAN	IPI00010163	ZNF286A Zinc finger protein 286A
104	2	12.87	2	ipi.HUMAN	IPI00022937	F5 Coagulation factor V
105	2	12.87	12	ipi.HUMAN	IPI00022394	Complement C1q subcomponent subunit C
106	2	12.99	4	ipi.HUMAN	IPI00025084	Calpain-small subunit 1
107	2	12.99	5	ipi.HUMAN	IPI00741240	LOC643501 similar to alpha 1 type I collagen preproprotein
108	2	13.02	6	ipi.HUMAN	IPI00303797	BRAF B-Raf proto-oncogene serine/threonine-protein kinase

#	Number of Peptides	Spectrum Mill Score	Percent Coverage	Database	Accession Number	Protein Name
109	3	13.03	7	ipi.HUMAN	IPI00064667	CNDP1 Beta-Ala-His dipeptidase precursor
110	2	13.09	7	ipi.HUMAN	IPI00009950	LMAN2 Vesicular integral-membrane protein VIP36 precursor
111	2	13.13	11	ipi.HUMAN	IPI00002745	CTSZ Cathepsin Z precursor
112	2	13.16	3	ipi.HUMAN	IPI00171611	Histone 3
113	2	13.21	9	ipi.HUMAN	IPI00011654	tubulin, beta-2
114	2	13.3	3	ipi.HUMAN	IPI00816779	Leukocyte antigen
115	2	13.33	4	ipi.HUMAN	IPI00071509	PKP1 Isoform 2 of Plakophilin-1
116	2	13.34	12	ipi.HUMAN	IPI00550363	transgelin-2
117	2	13.41	4	ipi.HUMAN	IPI00018953	DPP4 Dipeptidyl peptidase 4
118	2	13.41	2	ipi.HUMAN	IPI00397801	FLG2 Ifapsoriasin
119	2	13.45	4	ipi.HUMAN	IPI00295414	COL15A1 Collagen alpha-1(XV) chain precursor
120	2	13.64	9	ipi.HUMAN	IPI00445227	MCAM Isoform 2 of Cell surface glycoprotein MUC18 precursor
121	2	13.77	9	ipi.HUMAN	IPI00643034	PLTP Isoform 1 of Phospholipid transfer protein precursor
122	2	13.8	34	ipi.HUMAN	IPI00418471	Vimentin
123	2	13.87	4	ipi.HUMAN	IPI00884222	KRT10 Keratin, type I cytoskeletal 10
124	2	13.99	5	ipi.HUMAN	IPI00645194	ITGB1 Integrin beta-1
125	2	14.11	33	ipi.HUMAN	IPI00787049	LOC391322 similar to D-dopachrome decarboxylase
126	2	14.19	14	ipi.HUMAN	IPI00021842	apolipoprotein E
127	2	14.22	9	ipi.HUMAN	IPI00022391	APCS Serum amyloid P-component precursor
128	2	14.25	8	ipi.HUMAN	IPI00019190	myocilin
129	2	14.29	6	ipi.HUMAN	IPI00884176	F13A1 coagulation factor XIII A1 subunit precursor
130	2	14.34	6	ipi.HUMAN	IPI00296608	C7 Complement component C7 precursor
131	2	14.55	1	ipi.HUMAN	IPI00333541	FLNA Filamin-A
132	2	14.56	5	ipi.HUMAN	IPI00218694	CALD1 Isoform 2 of Caldesmon
133	2	14.68	1	ipi.HUMAN	IPI00025276	TNXB Isoform XB of Tenascin-X precursor
134	2	14.72	5	ipi.HUMAN	IPI00414676	HSP90AB1 Heat shock protein HSP 90-beta
135	2	14.88	13	ipi.HUMAN	IPI00384369	TPM1 Tropomyosin 1 alpha variant 6
136	2	15.06	6	ipi.HUMAN	IPI00303963	C2 Complement C2 precursor (Fragment)
137	2	15.13	44	ipi.HUMAN	IPI00013945	uromodulin precursor

#	Number of Peptides	Spectrum Mill Score	Percent Coverage	Database	Accession Number	Protein Name
138	2	15.35	16	ipi.HUMAN	IPI00004373	MBL2 Mannose-binding protein C precursor
139	2	15.47	33	ipi.HUMAN	IPI00216984	Calmodulin-like protein 3
140	2	15.48	16	ipi.HUMAN	IPI00879749	GSTA2 Glutathione S-transferase
141	3	15.49	2	ipi.HUMAN	IPI00183913	IGSF10 Isoform 1 of Immunoglobulin superfamily member 10 precursor
142	2	15.58	8	ipi.HUMAN	IPI00220644	PKM2 Isoform M1 of Pyruvate kinase isozymes M1/M2
143	2	15.59	4	ipi.HUMAN	IPI00220665	HK1 Isoform 3 of Hexokinase-1
144	3	15.69	10	ipi.HUMAN	IPI00844485	SYT14 Isoform 6 of Synaptotagmin-14
145	3	15.73	5	ipi.HUMAN	IPI00001985	VPS18 Isoform 1 of Vacuolar protein sorting-associated protein 18 homolog
146	3	15.92	7	ipi.HUMAN	IPI00295818	GEN1 Flap endonuclease GEN homolog 1
147	3	15.94	34	ipi.HUMAN	IPI00742239	similar to small nuclear ribonucleoprotein D2
148	3	15.98	3	ipi.HUMAN	IPI00029107	WRN Werner syndrome ATP-dependent helicase
149	2	16	4	ipi.HUMAN	IPI00745251	MAN1C1 Mannosyl-oligosaccharide 1,2-alpha-mannosidase IC
150	2	16.02	9	ipi.HUMAN	IPI00031708	Fumarylacetoacetase
151	3	16.03	6	ipi.HUMAN	IPI00219978	TDRD5 Isoform 1 of Tudor domain-containing protein 5
152	3	16.04	16	ipi.HUMAN	IPI00216065	PROZ Isoform 2 of Vitamin K-dependent protein Z precursor
153	3	16.11	4	ipi.HUMAN	IPI00020134	SOS1 Alternate SOS1
154	3	16.25	5	ipi.HUMAN	IPI00302647	CC2D1A Isoform 1 of Coiled-coil and C2 domain-containing protein 1A
155	3	16.27	1	ipi.HUMAN	IPI00788200	MYO7B myosin VIIB
156	3	16.33	3	ipi.HUMAN	IPI00456970	CDC2L5 Isoform 1 of Cell division cycle 2-like protein kinase 5
157	3	16.33	60	ipi.HUMAN	IPI00006113	POLR2I DNA-directed RNA polymerase II subunit RPB9
158	3	16.35	4	ipi.HUMAN	IPI00410188	CCDC146 coiled-coil domain containing 146
159	3	16.35	3	ipi.HUMAN	IPI00328825	N4BP2 Isoform 1 of NEDD4-binding protein 2
160	3	16.36	8	ipi.HUMAN	IPI00306749	SLC4A1AP Kanadaptin

#	Number of Peptides	Spectrum Mill Score	Percent Coverage	Database	Accession Number	Protein Name
161	3	16.37	9	ipi.HUMAN	IPI00787556	TXNRD3 similar to thioredoxin reductase 3
162	2	16.39	15	ipi.HUMAN	IPI00398735	CNN2 calponin 2 isoform b
163	3	16.39	3	ipi.HUMAN	IPI00064201	FRMPD3 FERM and PDZ domain-containing protein 3
164	3	16.41	12	ipi.HUMAN	IPI00747198	MICA
165	3	16.42	7	ipi.HUMAN	IPI00456737	RAB6IP1 Rab6-interacting protein 1
166	3	16.44	8	ipi.HUMAN	IPI00289929	ACRC ACRC protein
167	3	16.46	5	ipi.HUMAN	IPI00297211	SMARCA5 SWI/SNF-related matrix-associated actin-dependent regulator of chromatin subfamily A member 5
168	2	16.5	3	ipi.HUMAN	IPI00026944	nidogen
169	3	16.53	6	ipi.HUMAN	IPI00337386	PRPF40A formin binding protein 3
170	3	16.58	2	ipi.HUMAN	IPI00760846	MYO18A Isoform 1 of Myosin-XVIIIa
171	3	16.58	5	ipi.HUMAN	IPI00412209	SRGAP3 Isoform 2 of SLIT-ROBO Rho GTPase-activating protein 3
172	3	16.59	5	ipi.HUMAN	IPI00455518	MORC2 MORC family CW-type zinc finger protein 2
173	3	16.61	3	ipi.HUMAN	IPI00027883	MAST1 Microtubule-associated serine/threonine-protein kinase 1
174	3	16.62	14	ipi.HUMAN	IPI00786991	THA1P similar to R102.4b
175	3	16.66	3	ipi.HUMAN	IPI00414027	ITSN2 Intersectin-2 (SH3 domain-containing protein 1B) (SH3P18) (SH3P18-like WASP-associated protein). Isoform 4
176	2	16.68	1	ipi.HUMAN	IPI00031008	TNC Isoform 1 of Tenascin precursor
177	2	16.68	4	ipi.HUMAN	IPI00012011	coagulation factor XIII, B polypeptide
178	3	16.69	2	ipi.HUMAN	IPI00220289	CHD6 Isoform 1 of Chromodomain-helicase-DNA-binding protein 6
179	3	16.69	8	ipi.HUMAN	IPI00007256	ZHX2 Zinc fingers and homeoboxes protein 2
180	3	16.75	1	ipi.HUMAN	IPI00009066	PTPRQ similar to protein tyrosine phosphatase, receptor type, Q isoform 1 precursor
181	2	16.76	5	ipi.HUMAN	IPI00157365	EFTUD1
182	2	16.78	11	ipi.HUMAN	IPI00027487	CKM Creatine kinase M-type
183	3	16.8	7	ipi.HUMAN	IPI00289572	TPO Isoform 1 of Thyroid peroxidase precursor

#	Number of Peptides	Spectrum Mill Score	Percent Coverage	Database	Accession Number	Protein Name
184	3	16.81	6	ipi.HUMAN	IPI00031583	USO1 Putative uncharacterized protein DKFZp451D234
185	3	16.83	4	ipi.HUMAN	IPI00855985	MAP3K1 Mitogen-activated protein kinase kinase kinase 1
186	3	16.83	4	ipi.HUMAN	IPI00064162	VCPIP1 Deubiquitinating protein VCIP135
187	2	16.85	6	ipi.HUMAN	IPI00026259	NGLY1 Isoform 1 of Peptide-N(4)-(N-acetyl-beta-glucosaminyl)-L-asparaginase
188	3	16.85	2	ipi.HUMAN	IPI00297241	URB1 Nucleolar pre-ribosomal-associated protein 1
189	3	16.86	7	ipi.HUMAN	IPI00410256	CTDP1 Isoform 1 of RNA polymerase II subunit A C-terminal domain phosphatase
190	2	16.87	35	ipi.HUMAN	IPI00479867	Complement C1r subcomponent
191	3	16.88	18	ipi.HUMAN	IPI00013178	WNT5A Protein Wnt-5a precursor
192	3	16.91	9	ipi.HUMAN	IPI00293005	GABRR2 gamma-aminobutyric acid (GABA) receptor, rho 2 precursor
193	3	16.92	16	ipi.HUMAN	IPI00025447	Elongation factor 1-alpha_1
194	3	16.93	9	ipi.HUMAN	IPI00022790	MFAP1 Microfibrillar-associated protein 1
195	3	16.95	8	ipi.HUMAN	IPI00641705	IL17RD Isoform 1 of Interleukin-17 receptor D precursor
196	3	16.95	6	ipi.HUMAN	IPI00074966	YSK4 Isoform 1 of SPS1/STE20-related protein kinase YSK4
197	3	16.96	14	ipi.HUMAN	IPI00465105	DNAJA4 DNAJA4 protein
198	3	16.97	4	ipi.HUMAN	IPI00413517	ARHGEF10 Isoform 1 of Rho guanine nucleotide exchange factor 10
199	3	16.99	4	ipi.HUMAN	IPI00815998	CFTR Isoform 1 of Cystic fibrosis transmembrane conductance regulator
200	3	17	4	ipi.HUMAN	IPI00043978	PARD3B Isoform 1 of Partitioning-defective 3 homolog B
201	3	17.04	9	ipi.HUMAN	IPI00165261	SCFD1 Sec1 family domain-containing protein 1
202	3	17.05	5	ipi.HUMAN	IPI00021129	AP3B1 Isoform 1 of AP-3 complex subunit beta-1
203	3	17.05	8	ipi.HUMAN	IPI00376941	DDHD1 Isoform 1 of Phospholipase DDHD1
204	3	17.06	9	ipi.HUMAN	IPI00793839	CS 48 kDa protein

#	Number of Peptides	Spectrum Mill Score	Percent Coverage	Database	Accession Number	Protein Name
205	3	17.06	10	ipi.HUMAN	IPI00056349	FBXW7 Isoform 1 of F-box/WD repeat-containing protein 7
206	3	17.07	2	ipi.HUMAN	IPI00852708	BAT2D1 HBxAg transactivated protein 2
207	3	17.08	16	ipi.HUMAN	IPI00167638	GTPBP10 Isoform 1 of GTP-binding protein 10
208	3	17.09	8	ipi.HUMAN	IPI00102268	ZSWIM3 Zinc finger SWIM domain-containing protein 3
209	3	17.1	1	ipi.HUMAN	IPI00657953	TRIO triple functional domain
210	3	17.11	6	ipi.HUMAN	IPI00015963	TRPA1 Transient receptor potential cation channel subfamily A member 1
211	3	17.17	12	ipi.HUMAN	IPI00216905	CNGA4 cyclic nucleotide gated channel alpha 4
212	3	17.17	3	ipi.HUMAN	IPI00441959	NIN Isoform 7 of Ninein
213	3	17.21	7	ipi.HUMAN	IPI00027192	PLOD1 Procollagen-lysine,2-oxoglutarate 5-dioxygenase 1 precursor
214	3	17.21	2	ipi.HUMAN	IPI00011044	RP1 Oxygen-regulated protein 1
215	3	17.23	5	ipi.HUMAN	IPI00217630	DHX37 Probable ATP-dependent RNA helicase DHX37
216	3	17.24	7	ipi.HUMAN	IPI00329791	DDX46 cDNA FLJ78679, highly similar to Homo sapiens DEAD (Asp-Glu-Ala-Asp) box polypeptide 46 (DDX46), mRNA
217	3	17.25	6	ipi.HUMAN	IPI00514856	UBAP2L Isoform 1 of Ubiquitin-associated protein 2-like
218	3	17.27	9	ipi.HUMAN	IPI00154910	UBASH3B Ubiquitin associated and SH3 domain-containing protein B
219	3	17.28	17	ipi.HUMAN	IPI00026054	MAPKAPK2 Isoform 1 of MAP kinase-activated protein kinase 2
220	3	17.28	9	ipi.HUMAN	IPI00783751	RET tyrosine kinase/cAMP protein kinase A subunit RI
221	3	17.3	3	ipi.HUMAN	IPI00742726	ADCY10 soluble adenylyl cyclase
222	3	17.3	24	ipi.HUMAN	IPI00018510	SPIN2A Spindlin-2A
223	3	17.33	13	ipi.HUMAN	IPI00218297	HPD 4-hydroxyphenylpyruvate dioxygenase
224	3	17.33	4	ipi.HUMAN	IPI00000203	LRP6 Low-density lipoprotein receptor-related protein 6 precursor

#	Number of Peptides	Spectrum Mill Score	Percent Coverage	Database	Accession Number	Protein Name
225	3	17.36	2	ipi.HUMAN	IPI00418790	EML5 echinoderm microtubule associated protein like 5
226	3	17.37	4	ipi.HUMAN	IPI00015902	PDGFRB Beta-type platelet-derived growth factor receptor precursor
227	3	17.38	5	ipi.HUMAN	IPI00646226	MED23 Isoform 5 of Mediator of RNA polymerase II transcription subunit 23
228	3	17.4	4	ipi.HUMAN	IPI00654603	CCDC88A Isoform 2 of Girdin
229	3	17.4	5	ipi.HUMAN	IPI00293887	STARD8 StAR-related lipid transfer protein 8
230	3	17.42	6	ipi.HUMAN	IPI00856109	C8orf80 Isoform 1 of Uncharacterized protein C8orf80
231	3	17.44	5	ipi.HUMAN	IPI00384508	HEATR4 HEAT repeat-containing protein 4
232	3	17.44	7	ipi.HUMAN	IPI00551003	TBC1D19 TBC1 domain family member 19
233	3	17.45	8	ipi.HUMAN	IPI00002150	AP4B1 AP-4 complex subunit beta-1
234	3	17.46	3	ipi.HUMAN	IPI00872771	RAPGEF6 PDZ domain-containing guanine nucleotide exchange factor I
235	3	17.47	3	ipi.HUMAN	IPI00413100	PHLDB1 146 kDa protein
236	3	17.49	3	ipi.HUMAN	IPI00291783	GEMIN5 Gem-associated protein 5
237	3	17.5	11	ipi.HUMAN	IPI00216329	RGS6 Isoform 9 of Regulator of G-protein signaling 6
238	3	17.5	3	ipi.HUMAN	IPI00007362	UTX Ubiquitously transcribed X chromosome tetratricopeptide repeat protein
239	2	17.51	7	ipi.HUMAN	IPI00022417	LRG1 Leucine-rich alpha-2-glycoprotein precursor
240	3	17.52	16	ipi.HUMAN	IPI00796337	PCBP2 poly(rC)-binding protein 2 isoform a
241	3	17.54	15	ipi.HUMAN	IPI00032406	DNAJA2 DnaJ homolog subfamily A member 2
242	3	17.55	42	ipi.HUMAN	IPI00021391	CLEC2B C-type lectin domain family 2 member B
243	3	17.57	9	ipi.HUMAN	IPI00046057	STXBP1 Isoform 2 of Syntaxin-binding protein 1
244	3	17.58	12	ipi.HUMAN	IPI00386437	SPATA22 Isoform 1 of Spermatogenesis-associated protein 22
245	3	17.59	7	ipi.HUMAN	IPI00018931	VPS35 Vacuolar protein sorting-associated protein 35
246	3	17.6	8	ipi.HUMAN	IPI00029737	ACSL4 Isoform Long of Long-chain-fatty-acid--CoA ligase 4
247	3	17.6	14	ipi.HUMAN	IPI00019901	ADD1 Isoform 1 of Alpha-adducin

#	Number of Peptides	Spectrum Mill Score	Percent Coverage	Database	Accession Number	Protein Name
248	3	17.6	5	ipi.HUMAN	IPI00065515	CWF19L2 Isoform 2 of CWF19-like protein 2
249	3	17.6	3	ipi.HUMAN	IPI00398162	NRAP Isoform 2 of Nebulin-related-anchoring protein
250	3	17.61	5	ipi.HUMAN	IPI00397675	FHOD3 Isoform 1 of FH1/FH2 domain-containing protein 3
251	3	17.63	6	ipi.HUMAN	IPI00783931	COL18A1 Isoform 1 of Collagen alpha-1(XVIII) chain precursor
252	3	17.67	12	ipi.HUMAN	IPI00024214	TERF2 Isoform 1 of Telomeric repeat-binding factor 2
253	4	17.69	7	ipi.HUMAN	IPI00014575	CDC6 Cell division control protein 6 homolog
254	3	17.69	3	ipi.HUMAN	IPI00032087	DIP2C Disco-interacting protein 2 homolog C
255	2	17.69	24	ipi.HUMAN	IPI00300096	RAB35 Ras-related protein Rab-35
256	3	17.7	3	ipi.HUMAN	IPI00871686	CLASP2 Isoform 1 of CLIP-associating protein 2
257	3	17.71	4	ipi.HUMAN	IPI00759546	MLLT4 Isoform 5 of Afadin
258	3	17.73	11	ipi.HUMAN	IPI00872474	LYN Isoform LYN A of Tyrosine-protein kinase Lyn
259	3	17.75	5	ipi.HUMAN	IPI00549996	PDZRN4 Isoform 1 of PDZ domain-containing RING finger protein 4
260	3	17.75	1	ipi.HUMAN	IPI00749489	PKHD1L1 fibrocystin L
261	3	17.76	11	ipi.HUMAN	IPI00398796	RASSF6 Isoform 1 of Ras association domain-containing protein 6
262	3	17.78	14	ipi.HUMAN	IPI00332633	RASSF5 Isoform 1 of Ras association domain-containing family protein 5
263	3	17.8	11	ipi.HUMAN	IPI00170921	TIGD7 Isoform 1 of Tigger transposable element-derived protein 7
264	3	17.81	9	ipi.HUMAN	IPI00009070	HBS1L Isoform 1 of HBS1-like protein
265	3	17.82	4	ipi.HUMAN	IPI00480187	PHF8 Isoform 1 of PHD finger protein 8
266	3	17.84	7	ipi.HUMAN	IPI00852660	DCLK3 Serine/threonine-protein kinase DCLK3
267	3	17.85	8	ipi.HUMAN	IPI00646527	IRAK1 24 kDa protein
268	3	17.89	5	ipi.HUMAN	IPI00375339	ATP1A4 Isoform 1 of Sodium/potassium-transporting ATPase subunit alpha-4
269	3	17.89	5	ipi.HUMAN	IPI00023466	MLLT6 Protein AF-17
270	3	17.9	14	ipi.HUMAN	IPI00061178	CCBL2 kynurenine aminotransferase III isoform 3

#	Number of Peptides	Spectrum Mill Score	Percent Coverage	Database	Accession Number	Protein Name
271	3	17.9	3	ipi.HUMAN	IPI00013272	GOLGA4 Isoform 1 of Golgin subfamily A member 4
272	3	17.91	6	ipi.HUMAN	IPI00012738	CDK5R2 Cyclin-dependent kinase 5 activator 2 precursor
273	3	17.93	7	ipi.HUMAN	IPI00646839	EIF3C Eukaryotic translation initiation factor 3 subunit C
274	3	17.93	2	ipi.HUMAN	IPI00607818	MYH14 Isoform 2 of Myosin-14
275	3	17.93	3	ipi.HUMAN	IPI00782966	ZFP106 Zinc finger protein 106 homolog
276	3	17.97	5	ipi.HUMAN	IPI00877084	CCDC144C Isoform 1 of Coiled-coil domain-containing protein 144C
277	3	17.97	10	ipi.HUMAN	IPI00005782	PPM1D Protein phosphatase 1D
278	3	17.97	2	ipi.HUMAN	IPI00657805	XRN1 Isoform 1 of 5'-3' exoribonuclease 1
279	3	18	5	ipi.HUMAN	IPI00018889	GLI3 Zinc finger protein GLI3
280	3	18.01	7	ipi.HUMAN	IPI00023647	UBA6 Isoform 1 of Ubiquitin-like modifier-activating enzyme 6
281	3	18.04	9	ipi.HUMAN	IPI00736251	PPM1H Protein phosphatase 1H
282	3	18.04	2	ipi.HUMAN	IPI00165459	SETD1B similar to SET domain containing 1A
283	3	18.05	4	ipi.HUMAN	IPI00303136	DGKD Isoform 2 of Diacylglycerol kinase delta
284	3	18.06	7	ipi.HUMAN	IPI00005677	GNPAT Dihydroxyacetone phosphate acyltransferase
285	3	18.06	5	ipi.HUMAN	IPI00431791	MCTP1 Isoform 1 of Multiple C2 and transmembrane domain-containing protein 1
286	3	18.06	18	ipi.HUMAN	IPI00034280	MGC4172 Isoform 1 of Dehydrogenase/reductase SDR family member 11 precursor
287	3	18.08	3	ipi.HUMAN	IPI00024766	PLXNC1 Plexin-C1 precursor
288	3	18.1	10	ipi.HUMAN	IPI00642798	SC65 Nucleolar autoantigen No55
289	3	18.11	5	ipi.HUMAN	IPI00396048	C4orf41 hypothetical protein LOC60684 isoform a
290	3	18.12	6	ipi.HUMAN	IPI00182540	CTNND1 Isoform 1ABC of Catenin delta-1
291	3	18.12	6	ipi.HUMAN	IPI00339311	MCF2 MCF.2 cell line derived transforming sequence
292	3	18.12	5	ipi.HUMAN	IPI00023814	NEO1 Isoform 1 of Neogenin precursor
293	3	18.12	6	ipi.HUMAN	IPI00183487	XYLT1 Xylosyltransferase 1

#	Number of Peptides	Spectrum Mill Score	Percent Coverage	Database	Accession Number	Protein Name
294	3	18.13	4	ipi.HUMAN	IPI00299059	CHL1 Isoform 2 of Neural cell adhesion molecule L1-like protein precursor
295	3	18.13	8	ipi.HUMAN	IPI00396423	CLSTN3 Alcadein beta
296	3	18.13	1	ipi.HUMAN	IPI00014186	ZFHX3 Isoform A of Zinc finger homeobox protein 3
297	3	18.14	3	ipi.HUMAN	IPI00759642	CD163 Isoform 2 of Scavenger receptor cysteine-rich type 1 protein M130 precursor
298	3	18.14	7	ipi.HUMAN	IPI00385612	SLC8A2 Putative uncharacterized protein DKFZp761D171
299	3	18.16	13	ipi.HUMAN	IPI00293963	CDYL Isoform 1 of Chromodomain Y-like protein
300	3	18.18	10	ipi.HUMAN	IPI00030097	NTF4 Neurotrophin-5 precursor
301	3	18.21	8	ipi.HUMAN	IPI00878981	A26B3 ANKRD26-like family B member 3
302	3	18.23	6	ipi.HUMAN	IPI00174574	CCDC147 Coiled-coil domain-containing protein 147
303	2	18.23	7	ipi.HUMAN	IPI00002732	EXT2 Exostosin-2
304	3	18.23	5	ipi.HUMAN	IPI00396218	SCYL2 SCY1-like protein 2
305	3	18.25	5	ipi.HUMAN	IPI00164610	TBC1D1 TBC1 domain family member 1
306	3	18.26	5	ipi.HUMAN	IPI00295976	Integrin alpha-IIb precursor
307	3	18.27	5	ipi.HUMAN	IPI00470464	ZKSCAN2 Isoform 1 of Zinc finger protein with KRAB and SCAN domains 2
308	3	18.28	12	ipi.HUMAN	IPI00401758	ANKS3 Ankyrin repeat and SAM domain-containing protein 3
309	3	18.34	3	ipi.HUMAN	IPI00306851	LRP4 Low-density lipoprotein receptor-related protein 4 precursor
310	3	18.35	6	ipi.HUMAN	IPI00014312	CUL3 Isoform 1 of Cullin-3
311	3	18.35	5	ipi.HUMAN	IPI00418741	FUK Isoform 1 of L-fucose kinase
312	3	18.35	10	ipi.HUMAN	IPI00031050	SLC34A1 Sodium-dependent phosphate transport protein 2A
313	3	18.36	7	ipi.HUMAN	IPI00011219	ARHGAP6 Isoform 3 of Rho GTPase-activating protein 6
314	3	18.36	4	ipi.HUMAN	IPI00021250	DAPK1 Death-associated protein kinase 1
315	3	18.37	8	ipi.HUMAN	IPI00448879	AMPD1 AMP deaminase 1
316	3	18.38	6	ipi.HUMAN	IPI00217831	ANKRD13A Ankyrin repeat domain-containing protein 13A

#	Number of Peptides	Spectrum Mill Score	Percent Coverage	Database	Accession Number	Protein Name
317	3	18.38	8	ipi.HUMAN	IPI00385649	LRCH3 Isoform 2 of Leucine-rich repeat and calponin homology domain-containing protein 3 precursor
318	3	18.39	10	ipi.HUMAN	IPI00169430	STRBP Isoform 1 of Spermatid perinuclear RNA-binding protein
319	3	18.41	8	ipi.HUMAN	IPI00292499	HSPA14 Heat shock 70 kDa protein 14
320	3	18.42	1	ipi.HUMAN	IPI00394762	CSMD3 Isoform 4 of CUB and sushi domain-containing protein 3 precursor
321	3	18.44	3	ipi.HUMAN	IPI00455733	NHSL1 NHS-like 1
322	3	18.45	5	ipi.HUMAN	IPI00645643	BICC1 Isoform 1 of Protein bicaudal C homolog 1
323	3	18.45	9	ipi.HUMAN	IPI00329633	TARS Threonyl-tRNA synthetase, cytoplasmic
324	3	18.47	4	ipi.HUMAN	IPI00294787	RAD54L2 Helicase ARIP4
325	3	18.48	28	ipi.HUMAN	IPI00215914	ARF5 ADP-ribosylation factor 3
326	3	18.5	4	ipi.HUMAN	IPI00514030	COL4A6 Uncharacterized protein COL4A6
327	3	18.51	3	ipi.HUMAN	IPI00216219	TJP1 Isoform Long of Tight junction protein ZO-1
328	3	18.52	7	ipi.HUMAN	IPI00302434	HAP1 Isoform 1 of Huntingtin-associated protein 1
329	3	18.53	10	ipi.HUMAN	IPI00031115	GOLGA1 Golgin subfamily A member 1
330	3	18.54	4	ipi.HUMAN	IPI00299524	NCAPD2 Condensin complex subunit 1
331	3	18.54	2	ipi.HUMAN	IPI00301288	SVEP1 polydom
332	3	18.56	2	ipi.HUMAN	IPI00017603	F8 Coagulation factor VIII precursor
333	3	18.57	12	ipi.HUMAN	IPI00640240	SPTLC3 59 kDa protein
334	2	18.58	3	ipi.HUMAN	IPI00010290	fatty acid-binding protein, liver
335	3	18.59	4	ipi.HUMAN	IPI00853256	SYDE2 synapse defective 1, Rho GTPase, homolog 2
336	3	18.61	10	ipi.HUMAN	IPI00217791	CCDC105 Coiled-coil domain-containing protein 105
337	3	18.61	6	ipi.HUMAN	IPI00025094	MYH16 CDNA: FLJ22037 fis, clone HEP08868 (Fragment)
338	3	18.61	11	ipi.HUMAN	IPI00303402	RNUXA RNA U small nuclear RNA export adapter protein
339	3	18.61	4	ipi.HUMAN	IPI00411559	SMC4 Isoform 1 of Structural maintenance of chromosomes protein 4
340	3	18.62	2	ipi.HUMAN	IPI00844237	BAT2 HLA-B associated transcript-2
341	3	18.64	5	ipi.HUMAN	IPI00031549	DSC3 Isoform 3A of Desmocollin-3 precursor
342	3	18.65	20	ipi.HUMAN	IPI00794537	CLDN7 25 kDa protein

#	Number of Peptides	Spectrum Mill Score	Percent Coverage	Database	Accession Number	Protein Name
343	3	18.66	16	ipi.HUMAN	IPI00375527	IMPDH1 inosine monophosphate dehydrogenase 1 isoform a
344	3	18.66	41	ipi.HUMAN	IPI00185217	RAB1A Isoform 3 of Ras-related protein Rab-1A
345	3	18.67	4	ipi.HUMAN	IPI00004516	PER2 Isoform 1 of Period circadian protein homolog 2
346	3	18.67	10	ipi.HUMAN	IPI00783302	PTCD3 Isoform 1 of Pentatricopeptide repeat-containing protein 3, mitochondrial precursor
347	3	18.68	3	ipi.HUMAN	IPI00412415	BAZ1A Isoform 1 of Bromodomain adjacent to zinc finger domain protein 1A
348	3	18.68	3	ipi.HUMAN	IPI00006935	EIF5B Eukaryotic translation initiation factor 5AII
349	3	18.68	6	ipi.HUMAN	IPI00020996	IGFALS Insulin-like growth factor-binding protein complex acid labile chain precursor
350	3	18.68	6	ipi.HUMAN	IPI00871815	LNPEP 124 kDa protein
351	3	18.68	12	ipi.HUMAN	IPI00744230	MGAT4B mannosyl (alpha-1,3-)-glycoprotein beta-1,4-N-acetylglucosaminyltransferase, isoenzyme B isoform 2
352	3	18.68	4	ipi.HUMAN	IPI00010200	YTHDC2 YTH domain containing 2
353	3	18.69	5	ipi.HUMAN	IPI00646689	thioredoxin-like protein 5
354	3	18.71	5	ipi.HUMAN	IPI00478192	VEPH1 Isoform 1 of Ventricular zone-expressed PH domain-containing protein homolog 1
355	3	18.74	1	ipi.HUMAN	IPI00103595	CEP350 Centrosome-associated protein 350
356	3	18.75	12	ipi.HUMAN	IPI00022904	PPP4R2 55 kDa protein
357	3	18.75	5	ipi.HUMAN	IPI00104396	RGSL1 RGSL1 protein
358	3	18.75	5	ipi.HUMAN	IPI00884448	SSPO LOC643641 protein
359	3	18.76	7	ipi.HUMAN	IPI00301465	HJURP 14-3-3-associated AKT substrate
360	3	18.78	20	ipi.HUMAN	IPI00383160	ATP5S ATP synthase coupling factor B-like 1
361	3	18.78	3	ipi.HUMAN	IPI00607584	MYBBP1A Isoform 2 of Myb-binding protein 1A
362	3	18.78	6	ipi.HUMAN	IPI00303832	RTF1 Paf1/RNA polymerase II complex component
363	3	18.79	3	ipi.HUMAN	IPI00303112	CXXC6 CXXC-type zinc finger protein 6
364	3	18.79	10	ipi.HUMAN	IPI00221354	FUS Isoform Short of RNA-binding protein FUS
365	3	18.79	2	ipi.HUMAN	IPI00787921	SETD1B SET domain containing 1B
366	2	18.8	10	ipi.HUMAN	IPI00000874	peroxiredoxin 1

#	Number of Peptides	Spectrum Mill Score	Percent Coverage	Database	Accession Number	Protein Name
367	3	18.8	2	ipi.HUMAN	IPI00328550	thrombomodulin-4 precursor
368	3	18.84	5	ipi.HUMAN	IPI00847504	PLCL2 Isoform 1 of Inactive phospholipase C-like protein 2
369	3	18.86	6	ipi.HUMAN	IPI00456694	DDX26B Isoform 1 of Protein DDX26B
370	3	18.87	6	ipi.HUMAN	IPI00514295	CIZ1 Cip1-interacting zinc finger protein
371	3	18.87	6	ipi.HUMAN	IPI00295388	GLDC 115 kDa protein
372	3	18.87	8	ipi.HUMAN	IPI00016861	GTF3C2 102 kDa protein
373	3	18.87	5	ipi.HUMAN	IPI00003802	MAN2A1 Alpha-mannosidase 2
374	3	18.88	3	ipi.HUMAN	IPI00470537	ARID2 Isoform 1 of AT-rich interactive domain-containing protein 2
375	3	18.88	8	ipi.HUMAN	IPI00019359	KRT9 Keratin, type I cytoskeletal 9
376	3	18.88	19	ipi.HUMAN	IPI00017558	SFRP5 Secreted frizzled-related protein 5 precursor
377	3	18.92	6	ipi.HUMAN	IPI00884093	TEK TEK tyrosine kinase, endothelial precursor
378	3	18.93	2	ipi.HUMAN	IPI00414482	GTF3C1 Isoform 1 of General transcription factor 3C polypeptide 1
379	3	18.94	11	ipi.HUMAN	IPI00011306	CCDC130 Coiled-coil domain-containing protein 130
380	3	18.94	7	ipi.HUMAN	IPI00030880	GRIA1 Isoform Flop of Glutamate receptor 1 precursor
381	3	18.95	13	ipi.HUMAN	IPI00180404	NEXN Isoform 1 of Nexilin
382	3	18.97	8	ipi.HUMAN	IPI00433284	GLCE D-glucuronyl C5-epimerase
383	3	18.98	6	ipi.HUMAN	IPI00217830	TMEM67 Isoform 1 of Meckelin
384	3	18.99	10	ipi.HUMAN	IPI00030847	TM9SF3 Transmembrane 9 superfamily member 3 precursor
385	3	19.01	4	ipi.HUMAN	IPI00249249	RIMS2 Isoform 6 of Regulating synaptic membrane exocytosis protein 2
386	3	19.01	11	ipi.HUMAN	IPI00399220	USP27X Ubiquitin carboxyl-terminal hydrolase 27
387	3	19.02	5	ipi.HUMAN	IPI00103487	NLRP7 NLRP7 protein
388	3	19.03	8	ipi.HUMAN	IPI00477479	RNF19B Isoform 1 of E3 ubiquitin-protein ligase RNF19B
389	3	19.05	5	ipi.HUMAN	IPI00644127	IARS Isoleucyl-tRNA synthetase, cytoplasmic
390	3	19.05	5	ipi.HUMAN	IPI00297723	RBM6 RNA-binding protein 6

#	Number of Peptides	Spectrum Mill Score	Percent Coverage	Database	Accession Number	Protein Name
391	3	19.06	10	ipi.HUMAN	IPI00029045	IKBKE Inhibitor of nuclear factor kappa-B kinase subunit epsilon
392	3	19.06	3	ipi.HUMAN	IPI00384909	KIDINS220 KIDINS220 protein
393	3	19.06	4	ipi.HUMAN	IPI00218089	SLC4A5 sodium bicarbonate transporter 4 isoform a
394	3	19.07	1	ipi.HUMAN	IPI00186711	PLEC1 plectin 1 isoform 6
395	3	19.07	6	ipi.HUMAN	IPI00176345	UNC5A Isoform 3 of Netrin receptor UNC5A precursor
396	3	19.08	14	ipi.HUMAN	IPI00152159	GSG1L GSG1-like isoform 1
397	3	19.08	13	ipi.HUMAN	IPI00784154	HSPD1 60 kDa heat shock protein, mitochondrial precursor
398	3	19.08	8	ipi.HUMAN	IPI00640027	ODF2 Isoform 1 of Outer dense fiber protein 2
399	3	19.09	18	ipi.HUMAN	IPI00179473	SQSTM1 Isoform 1 of Sequestosome-1
400	3	19.1	10	ipi.HUMAN	IPI00719231	FMNL1 FMNL1 protein
401	3	19.1	5	ipi.HUMAN	IPI00784013	JAK1 janus kinase 1
402	3	19.1	5	ipi.HUMAN	IPI00413022	RNF216 Isoform 2 of E3 ubiquitin-protein ligase RNF216
403	3	19.11	7	ipi.HUMAN	IPI00007913	ARHGAP26 Isoform 1 of Rho GTPase-activating protein 26
404	3	19.11	4	ipi.HUMAN	IPI00413880	MAGI2 Isoform 1 of Membrane-associated guanylate kinase, WW and PDZ domain-containing protein 2
405	3	19.13	10	ipi.HUMAN	IPI00554494	FBXL19 F-box and leucine-rich repeat protein 19
406	3	19.14	10	ipi.HUMAN	IPI00025698	LIMK2 LIM domain kinase 2 isoform 1
407	3	19.14	15	ipi.HUMAN	IPI00411715	NFKB2 Isoform 2 of Nuclear factor NF-kappa-B p100 subunit
408	3	19.14	1	ipi.HUMAN	IPI00221012	USP9X ubiquitin specific protease 9, X-linked isoform 3
409	4	19.15	5	ipi.HUMAN	IPI00001245	CNTNAP3 Isoform 1 of Contactin-associated protein-like 3 precursor
410	3	19.15	16	ipi.HUMAN	IPI00016005	FMO2 Dimethylaniline monooxygenase [N-oxide-forming] 2

#	Number of Peptides	Spectrum Mill Score	Percent Coverage	Database	Accession Number	Protein Name
411	3	19.15	3	ipi.HUMAN	IPI00018321	SETDB1 Isoform 1 of Histone-lysine N-methyltransferase SETDB1
412	3	19.16	3	ipi.HUMAN	IPI00465102	POLE DNA polymerase epsilon catalytic subunit A
413	3	19.17	6	ipi.HUMAN	IPI00001496	PCDHA5 Isoform 1 of Protocadherin alpha 5 precursor
414	3	19.17	5	ipi.HUMAN	IPI00217804	SLFN12 Schlafen family member 12
415	3	19.21	14	ipi.HUMAN	IPI00014939	MKKS McKusick-Kaufman/Bardet-Biedl syndromes putative chaperonin
416	3	19.22	3	ipi.HUMAN	IPI00871202	BAZ2A 215 kDa protein
417	3	19.22	7	ipi.HUMAN	IPI00219434	DLGAP3 Disks large-associated protein 3
418	3	19.22	2	ipi.HUMAN	IPI00852639	SCN5A voltage-gated sodium channel type V alpha isoform c
419	3	19.25	11	ipi.HUMAN	IPI00043373	CEP63 Isoform 1 of Centrosomal protein of 63 kDa
420	3	19.26	6	ipi.HUMAN	IPI00792423	MED24 Thyroid hormone receptor-associated protein complex 100 kDa component
421	3	19.27	12	ipi.HUMAN	IPI00217795	PGS1 Phosphatidylglycerophosphate synthase 1
422	3	19.27	13	ipi.HUMAN	IPI00395914	SCARA5 Isoform 1 of Scavenger receptor class A member 5
423	3	19.29	2	ipi.HUMAN	IPI00010604	PLCE1 Isoform 1 of 1-phosphatidylinositol-4,5-bisphosphate phosphodiesterase epsilon-1
424	3	19.31	5	ipi.HUMAN	IPI00296421	EHBP1L1 EH domain-binding protein 1-like protein 1
425	3	19.31	8	ipi.HUMAN	IPI00478124	UCKL1 61 kDa protein
426	3	19.32	2	ipi.HUMAN	IPI00333405	FER1L6 fer-1-like 6
427	3	19.34	10	ipi.HUMAN	IPI00298373	ALS2CR13 Isoform 1 of Amyotrophic lateral sclerosis 2 chromosomal region candidate gene 13 protein
428	3	19.35	5	ipi.HUMAN	IPI00787531	GAK Cyclin G-associated kinase
429	3	19.37	6	ipi.HUMAN	IPI00792186	ABCF1 ATP-binding cassette, sub-family F (GCN20), member 1
430	3	19.37	8	ipi.HUMAN	IPI00329561	RAPGEF4 Isoform 2 of Rap guanine nucleotide exchange factor 4
431	3	19.41	6	ipi.HUMAN	IPI00005565	DGKQ Diacylglycerol kinase theta
432	3	19.41	6	ipi.HUMAN	IPI00873518	MAD1L1 MAD1 mitotic arrest deficient-like 1

#	*Number of Peptides*	*Spectrum Mill Score*	*Percent Coverage*	*Database*	*Accession Number*	*Protein Name*
433	3	19.42	6	ipi.HUMAN	IPI00741826	DKFZp779B1634 similar to ankyrin repeat domain 26 isoform 4
434	3	19.42	3	ipi.HUMAN	IPI00026625	NUP155 Isoform 1 of Nuclear pore complex protein Nup155
435	3	19.42	4	ipi.HUMAN	IPI00400812	SSFA2 Isoform 1 of Sperm-specific antigen 2
436	3	19.43	7	ipi.HUMAN	IPI00181285	CDY2B
437	3	19.44	2	ipi.HUMAN	IPI00297089	AKAP6 A-kinase anchor protein 6
438	3	19.45	3	ipi.HUMAN	IPI00552671	PLXNA1 Plexin-A1 precursor
439	3	19.45	10	ipi.HUMAN	IPI00031608	SPATA5L1 Spermatogenesis-associated protein 5-like protein 1
440	3	19.46	2	ipi.HUMAN	IPI00786844	NOTCH2 similar to notch 2 preproprotein
441	3	19.46	3	ipi.HUMAN	IPI00007928	PRPF8 Pre-mRNA-processing-splicing factor 8
442	3	19.47	5	ipi.HUMAN	IPI00017450	GTF2F1 Transcription initiation factor IIF subunit alpha
443	3	19.47	7	ipi.HUMAN	IPI00024684	MX2 Interferon-induced GTP-binding protein Mx2
444	3	19.48	6	ipi.HUMAN	IPI00031407	NEDD9 Enhancer of filamentation 1
445	3	19.48	13	ipi.HUMAN	IPI00017870	Keratin-8-like protein 1
446	3	19.49	2	ipi.HUMAN	IPI00397474	ESPL1 Isoform 1 of Separin
447	3	19.49	3	ipi.HUMAN	IPI00298994	TLN1 Talin-1
448	3	19.51	3	ipi.HUMAN	IPI00871745	JARID1A Histone demethylase JARID1A
449	3	19.51	4	ipi.HUMAN	IPI00783392	RB1CC1 RB1-inducible coiled-coil protein 1
450	3	19.51	5	ipi.HUMAN	IPI00412441	WAPAL Isoform 3 of Wings apart-like protein homolog
451	3	19.53	21	ipi.HUMAN	IPI00292793	MDFIC Isoform 1 of MyoD family inhibitor domain-containing protein
452	3	19.53	6	ipi.HUMAN	IPI00020060	SLC9A1 Isoform 1 of Sodium/hydrogen exchanger 1
453	3	19.53	8	ipi.HUMAN	IPI00829637	SYTL5 protein
454	3	19.55	3	ipi.HUMAN	IPI00410666	SCRIB Isoform 3 of Protein LAP4
455	3	19.56	7	ipi.HUMAN	IPI00807722	CCDC81 Isoform 1 of Coiled-coil domain-containing protein 81
456	3	19.57	3	ipi.HUMAN	IPI00745991	PTPRB protein tyrosine phosphatase, receptor type, B isoform a

#	Number of Peptides	Spectrum Mill Score	Percent Coverage	Database	Accession Number	Protein Name
457	3	19.59	8	ipi.HUMAN	IPI00292134	EPS15 Epidermal growth factor receptor substrate 15
458	3	19.6	9	ipi.HUMAN	IPI00015505	HPS6 Hermansky-Pudlak syndrome 6 protein
459	3	19.6	11	ipi.HUMAN	IPI00334013	LCA5 Uncharacterized protein C6orf152
460	3	19.6	4	ipi.HUMAN	IPI00294173	RP4-691N24.1 Ninein-like protein
461	3	19.62	1	ipi.HUMAN	IPI00873889	LAMA2 Uncharacterized protein LAMA2
462	3	19.63	5	ipi.HUMAN	IPI00289131	TMEM8 Transmembrane protein 8 precursor
463	3	19.65	8	ipi.HUMAN	IPI00783982	COPG Coatomer subunit gamma
464	3	19.66	2	ipi.HUMAN	IPI00746049	ANKRD30B Similar to Ankyrin repeat domain-containing protein 30B
465	3	19.68	5	ipi.HUMAN	IPI00001872	PCDHGC3
466	3	19.68	1	ipi.HUMAN	IPI00221325	RANBP2 E3 SUMO-protein ligase RanBP2
467	3	19.68	9	ipi.HUMAN	IPI00873762	TAF15 65 kDa protein
468	3	19.7	5	ipi.HUMAN	IPI00456707	ERC2 Isoform 2 of ERC protein 2
469	3	19.7	1	ipi.HUMAN	IPI00788121	ODZ3 Teneurin-3
470	3	19.74	6	ipi.HUMAN	IPI00398009	IPO4 Isoform 2 of Importin-4
471	3	19.76	20	ipi.HUMAN	IPI00071318	LUC7L Uncharacterized protein LUC7L
472	3	19.78	10	ipi.HUMAN	IPI00009315	ACBD3 Golgi resident protein GCP60
473	3	19.79	8	ipi.HUMAN	IPI00335946	FAM120B Family with sequence similarity 120B
474	3	19.8	10	ipi.HUMAN	IPI00382412	BCAT1 Branched-chain-amino-acid aminotransferase, cytosolic
475	3	19.8	8	ipi.HUMAN	IPI00218763	CAPN3 Isoform II of Calpain-3
476	3	19.8	8	ipi.HUMAN	IPI00169342	RBM47 Isoform 1 of RNA-binding protein 47
477	3	19.82	12	ipi.HUMAN	IPI00477526	C7orf28A
478	3	19.82	13	ipi.HUMAN	IPI00289746	PAK1 Isoform 2 of Serine/threonine-protein kinase PAK 1
479	3	19.82	5	ipi.HUMAN	IPI00007927	SMC2 Isoform 1 of Structural maintenance of chromosomes protein 2
480	3	19.82	3	ipi.HUMAN	IPI00021428	actin, alpha 1, skeletal muscle
481	3	19.83	7	ipi.HUMAN	IPI00218730	PDE6A Rod cGMP-specific 3',5'-cyclic phosphodiesterase subunit alpha
482	3	19.85	10	ipi.HUMAN	IPI00014168	HSAJ2425 Protein p65

#	Number of Peptides	Spectrum Mill Score	Percent Coverage	Database	Accession Number	Protein Name
483	3	19.85	7	ipi.HUMAN	IPI00292709	PCK1 Phosphoenolpyruvate carboxykinase, cytosolic
484	3	19.86	19	ipi.HUMAN	IPI00016900	GPR173 Probable G-protein coupled receptor 173
485	3	19.88	3	ipi.HUMAN	IPI00220445	COL20A1 Isoform 1 of Collagen alpha-1(XX) chain precursor
486	3	19.9	3	ipi.HUMAN	IPI00012851	ATP8B1 Probable phospholipid-transporting ATPase IC
487	3	19.9	1	ipi.HUMAN	IPI00479143	PCNT Pericentrin
488	3	19.91	3	ipi.HUMAN	IPI00398837	CCDC88B coiled-coil domain containing 88
489	3	19.91	1	ipi.HUMAN	IPI00797574	FAM44A Protein FAM44A
490	2	19.91	20	ipi.HUMAN	IPI00019399	SAA4 Serum amyloid A-4 protein precursor
491	3	19.91	7	ipi.HUMAN	IPI00410477	XKR5 Isoform 1 of XK-related protein 5
492	3	19.92	8	ipi.HUMAN	IPI00216159	GFPT2 Glucosamine--fructose-6-phosphate aminotransferase [isomerizing] 2
493	3	19.92	9	ipi.HUMAN	IPI00418531	GLDN Isoform 1 of Gliomedin
494	3	19.93	4	ipi.HUMAN	IPI00658203	C9orf84 Isoform 1 of Uncharacterized protein C9orf84
495	3	19.93	16	ipi.HUMAN	IPI00216040	CCNE1 Isoform E1S of G1/S-specific cyclin-E1
496	3	19.95	7	ipi.HUMAN	IPI00162199	DKFZP434B0335 DKFZP434B0335 protein
497	3	19.98	9	ipi.HUMAN	IPI00477729	ACOX1 Isoform 2 of Acyl-coenzyme A oxidase 1, peroxisomal
498	3	19.98	8	ipi.HUMAN	IPI00470372	LRIG1 Isoform 2 of Leucine-rich repeats and immunoglobulin-like domains protein 1 precursor
499	3	20	6	ipi.HUMAN	IPI00032358	POM121 Nuclear envelope pore membrane protein POM 121
500	3	20.01	9	ipi.HUMAN	IPI00873424	ABLIM1 Uncharacterized protein ABLIM1
501	3	20.03	4	ipi.HUMAN	IPI00216047	SMARCC2 Isoform 1 of SWI/SNF-related matrix-associated actin-dependent regulator of chromatin subfamily C member 2
502	3	20.04	2	ipi.HUMAN	IPI00844585	ABCA4 ABCA4 variant protein
503	3	20.04	7	ipi.HUMAN	IPI00185219	GAS2L3 GAS2-like protein 3
504	3	20.04	1	ipi.HUMAN	IPI00871227	HMCN1 Isoform 1 of Hemicentin-1 precursor

#	Number of Peptides	Spectrum Mill Score	Percent Coverage	Database	Accession Number	Protein Name
505	3	20.06	21	ipi.HUMAN	IPI00871355	SAC3D1 SAC3 domain containing 1
506	3	20.06	17	ipi.HUMAN	IPI00002970	SGCB Beta-sarcoglycan
507	3	20.09	10	ipi.HUMAN	IPI00215637	DDX3X ATP-dependent RNA helicase DDX3X
508	3	20.09	4	ipi.HUMAN	IPI00855787	TNKS1BP1 tankyrase 1-binding protein 1
509	3	20.1	3	ipi.HUMAN	IPI00240793	ATP11B Probable phospholipid-transporting ATPase IF
510	3	20.1	4	ipi.HUMAN	IPI00024970	INCENP Inner centromere protein
511	3	20.1	8	ipi.HUMAN	IPI00337541	NNT NAD(P) transhydrogenase, mitochondrial precursor
512	3	20.14	4	ipi.HUMAN	IPI00856117	TUBGCP5 tubulin, gamma complex associated protein 5 isoform b
513	3	20.16	13	ipi.HUMAN	IPI00749398	SERPINB6 Serpin B6
514	3	20.17	5	ipi.HUMAN	IPI00646493	COPA coatomer protein complex, subunit alpha isoform 1
515	3	20.18	3	ipi.HUMAN	IPI00304817	SASH1 SAM and SH3 domain-containing protein 1
516	3	20.18	6	ipi.HUMAN	IPI00470669	SSX2IP Isoform 1 of Afadin- and alpha-actinin-binding protein
517	3	20.19	6	ipi.HUMAN	IPI00220510	ISOFORM 7 OF MAP/MICROTUBULE AFFINITY-REGULATING KINASE 3
518	3	20.21	8	ipi.HUMAN	IPI00741630	WIZ Isoform 2 of Protein Wiz
519	3	20.22	5	ipi.HUMAN	IPI00743284	MTR Methionine synthase
520	3	20.23	7	ipi.HUMAN	IPI00102377	ANKRD27 Ankyrin repeat domain-containing protein 27
521	3	20.23	3	ipi.HUMAN	IPI00219299	TLN2 Talin-2
522	3	20.24	17	ipi.HUMAN	IPI00294469	COQ4 Ubiquinone biosynthesis protein COQ4 homolog
523	2	20.24	1	ipi.HUMAN	IPI00028413	ITIH3 Isoform 1 of Inter-alpha-trypsin inhibitor heavy chain H3 precursor
524	3	20.25	11	ipi.HUMAN	IPI00844469	PLEKHN1 Isoform 1 of Pleckstrin homology domain-containing family N member 1
525	3	20.26	3	ipi.HUMAN	IPI00607554	USP47 Isoform 1 of Ubiquitin carboxyl-terminal hydrolase 47

#	Number of Peptides	Spectrum Mill Score	Percent Coverage	Database	Accession Number	Protein Name
526	3	20.27	5	ipi.HUMAN	IPI00419518	ANKRD32 BRCT domain containing protein
527	3	20.27	7	ipi.HUMAN	IPI00853270	JAKMIP1 janus kinase and microtubule interacting protein 1 isoform 1
528	3	20.28	6	ipi.HUMAN	IPI00032137	ACTN3 Alpha-actinin-3
529	3	20.29	26	ipi.HUMAN	IPI00007019	PPIL1 Peptidyl-prolyl cis-trans isomerase-like 1
530	3	20.3	8	ipi.HUMAN	IPI00514832	TRIM56 Isoform 1 of Tripartite motif-containing protein 56
531	3	20.31	2	ipi.HUMAN	IPI00018279	COL5A3 Collagen alpha-3(V) chain precursor
532	3	20.31	5	ipi.HUMAN	IPI00301317	CTNND2 Isoform 1 of Catenin delta-2
533	3	20.33	5	ipi.HUMAN	IPI00152540	CD109 Isoform 1 of CD109 antigen precursor
534	3	20.33	2	ipi.HUMAN	IPI00413794	DOPEY1 Protein dopey-1
535	3	20.33	4	ipi.HUMAN	IPI00005704	ST18 Suppression of tumorigenicity protein 18
536	3	20.34	8	ipi.HUMAN	IPI00396627	ELAC2 Isoform 1 of Zinc phosphodiesterase ELAC protein 2
537	3	20.34	5	ipi.HUMAN	IPI00828028	KIF24 Isoform 1 of Kinesin-like protein KIF24
538	3	20.37	5	ipi.HUMAN	IPI00437751	ACE Isoform Somatic-1 of Angiotensin-converting enzyme, somatic isoform precursor
539	3	20.41	8	ipi.HUMAN	IPI00743749	PCNXL3 pecanex-like 3
540	3	20.42	1	ipi.HUMAN	IPI00456492	CROCC Isoform 1 of Rootletin
541	3	20.42	2	ipi.HUMAN	IPI00289034	DOT1L Isoform 2 of Histone-lysine N-methyltransferase, H3 lysine-79 specific
542	3	20.42	12	ipi.HUMAN	IPI00386377	SYT9 Synaptotagmin-9
543	3	20.43	4	ipi.HUMAN	IPI00783156	BMPR2 Bone morphogenetic protein receptor type-2 precursor
544	3	20.43	5	ipi.HUMAN	IPI00719817	DUOX1 Isoform 2 of Dual oxidase 1 precursor
545	3	20.43	4	ipi.HUMAN	IPI00792333	IBTK Isoform 1 of Inhibitor of Bruton tyrosine kinase
546	3	20.44	4	ipi.HUMAN	IPI00410588	ADAMTSL3 ADAMTS-like protein 3 precursor
547	3	20.46	26	ipi.HUMAN	IPI00001960	CLIC4 Chloride intracellular channel protein 4
548	3	20.46	13	ipi.HUMAN	IPI00102804	GRK7 G protein-coupled receptor kinase 7 precursor
549	3	20.46	5	ipi.HUMAN	IPI00024953	PRDM9 PR domain-containing 9

#	Number of Peptides	Spectrum Mill Score	Percent Coverage	Database	Accession Number	Protein Name
550	3	20.48	1	ipi.HUMAN	IPI00220624	AKAP9 A-kinase anchor protein 9 (Protein kinase A-anchoring protein 9) (PRKA9) (A-kinase anchor protein 450 kDa) (AKAP 450) (A-kinase anchor protein 350 kDa) (AKAP 350) (hgAKAP 350) (AKAP 120-like protein) (Protein hyperion) (Protein yotiao) (Centrosome- and Golgi-localized PKN-associated protein) (CG-NAP). Isoform 2
551	3	20.49	6	ipi.HUMAN	IPI00004344	AFF4 Isoform 1 of AF4/FMR2 family member 4
552	3	20.49	1	ipi.HUMAN	IPI00556369	SMG1 Isoform 1 of Serine/threonine-protein kinase SMG1
553	3	20.5	5	ipi.HUMAN	IPI00302133	TRPV5 Transient receptor potential cation channel subfamily V member 5
554	3	20.51	6	ipi.HUMAN	IPI00166339	EPHA10 Isoform 1 of Ephrin type-A receptor 10 precursor
555	3	20.51	7	ipi.HUMAN	IPI00744637	RUFY1 Isoform 1 of RUN and FYVE domain-containing protein 1
556	3	20.52	2	ipi.HUMAN	IPI00414303	ABCA2 ATP-binding cassette, sub-family A, member 2 isoform b
557	3	20.52	9	ipi.HUMAN	IPI00027497	GPI Glucose-6-phosphate isomerase
558	3	20.52	4	ipi.HUMAN	IPI00449049	PARP1 Poly [ADP-ribose] polymerase 1
559	3	20.52	7	ipi.HUMAN	IPI00442171	PREPL Isoform 1 of Prolyl endopeptidase-like
560	4	20.53	10	ipi.HUMAN	IPI00448121	CDC7 Cell division cycle 7-related protein kinase
561	3	20.54	10	ipi.HUMAN	IPI00018671	Dual specificity protein phosphatase 3
562	3	20.55	13	ipi.HUMAN	IPI00788599	TBXAS1 thromboxane A synthase 1 (platelet, cytochrome P450, family 5, subfamily A) isoform TXS-I
563	3	20.57	5	ipi.HUMAN	IPI00464980	SIN3B Isoform 1 of Paired amphipathic helix protein Sin3b
564	2	20.59	5	ipi.HUMAN	IPI00026314	GSN Isoform 1 of Gelsolin precursor
565	3	20.59	5	ipi.HUMAN	IPI00026580	OFD1 Isoform 1 of Oral-facial-digital syndrome 1 protein

#	Number of Peptides	Spectrum Mill Score	Percent Coverage	Database	Accession Number	Protein Name
566	3	20.59	5	ipi.HUMAN	IPI00291711	PLEKHG3 Isoform 1 of Pleckstrin homology domain-containing family G member 3
567	3	20.59	6	ipi.HUMAN	IPI00002548	STON1 Stonin-1
568	3	20.62	62	ipi.HUMAN	IPI00816686	RRN3 RNA polymerase I-specific transcription initiation factor RRN3
569	3	20.65	16	ipi.HUMAN	IPI00012828	ACAA1 3-ketoacyl-CoA thiolase, peroxisomal precursor
570	3	20.68	2	ipi.HUMAN	IPI00719285	CIT Citron
571	3	20.69	3	ipi.HUMAN	IPI00873930	PLEKHG4B 142 kDa protein
572	3	20.7	12	ipi.HUMAN	IPI00386258	MTCH1 CGI-64 protein
573	3	20.7	5	ipi.HUMAN	IPI00418275	ROBO3 Isoform 1 of Roundabout homolog 3 precursor
574	3	20.71	8	ipi.HUMAN	IPI00014021	BMP1 Isoform BMP1-1 of Bone morphogenetic protein 1 precursor
575	3	20.74	10	ipi.HUMAN	IPI00329141	CHSY1 Chondroitin sulfate synthase 1
576	3	20.75	8	ipi.HUMAN	IPI00013290	HDGF2 hepatoma-derived growth factor-related protein 2 isoform 1
577	3	20.75	11	ipi.HUMAN	IPI00441473	PRMT5 Protein arginine N-methyltransferase 5
578	3	20.76	1	ipi.HUMAN	IPI00884018	CTTNBP2 CTTNBP2
579	3	20.76	17	ipi.HUMAN	IPI00414463	LCMT1 Isoform 2 of Leucine carboxyl methyltransferase 1
580	3	20.78	16	ipi.HUMAN	IPI00011685	COL10A1 Collagen alpha-1(X) chain precursor
581	3	20.78	3	ipi.HUMAN	IPI00737429	ODZ4 Teneurin-4
582	3	20.79	6	ipi.HUMAN	IPI00473136	CTNNA1 Isoform 2 of Catenin alpha-1
583	3	20.79	5	ipi.HUMAN	IPI00873446	NRCAM Isoform 5 of Neuronal cell adhesion molecule precursor
584	3	20.79	8	ipi.HUMAN	IPI00641288	TRIM67 Isoform 2 of Tripartite motif-containing protein 67
585	3	20.8	1	ipi.HUMAN	IPI00306929	MYO18B Isoform 2 of Myosin-XVIIIb
586	3	20.8	4	ipi.HUMAN	IPI00374748	TMC3 Isoform 1 of Transmembrane channel-like protein 3
587	3	20.81	8	ipi.HUMAN	IPI00019907	GPC3 Glypican-3 precursor

#	Number of Peptides	Spectrum Mill Score	Percent Coverage	Database	Accession Number	Protein Name
588	3	20.82	5	ipi.HUMAN	IPI00015117	LAMC2 Isoform Long of Laminin subunit gamma-2 precursor
589	3	20.82	18	ipi.HUMAN	IPI00411356	VPS4A Vacuolar protein sorting-associating protein 4A
590	3	20.84	13	ipi.HUMAN	IPI00009634	SQRDL Sulfide:quinone oxidoreductase, mitochondrial precursor
591	3	20.87	8	ipi.HUMAN	IPI00871296	ATP2A3 118 kDa protein
592	3	20.87	6	ipi.HUMAN	IPI00011822	N4BP2L2 phosphonoformate immuno-associated protein 5 isoform 1
593	3	20.88	6	ipi.HUMAN	IPI00550503	TTC14 Isoform 1 of Tetratricopeptide repeat protein 14
594	3	20.89	5	ipi.HUMAN	IPI00470407	NARG2 Isoform 1 of NMDA receptor-regulated protein 2
595	3	20.9	5	ipi.HUMAN	IPI00872635	ATP2C2 Calcium-transporting ATPase type 2C member 2
596	3	20.93	3	ipi.HUMAN	IPI00878288	ATG2A Isoform 3 of Autophagy-related protein 2 homolog A
597	3	20.93	18	ipi.HUMAN	IPI00008669	KRT81 Keratin type II cuticular Hb1
598	3	20.93	18	ipi.HUMAN	IPI00027763	SLC25A16 Grave disease carrier protein
599	3	20.95	17	ipi.HUMAN	IPI00154618	FEZF2 Isoform 1 of Fez family zinc finger protein 2
600	3	20.95	3	ipi.HUMAN	IPI00166528	RICTOR Isoform 3 of Rapamycin-insensitive companion of mTOR
601	3	20.95	6	ipi.HUMAN	IPI00165984	WDR35 Isoform 1 of WD repeat-containing protein 35
602	3	20.96	4	ipi.HUMAN	IPI00162547	LPHN3 latrophilin 3 precursor
603	3	20.96	3	ipi.HUMAN	IPI00217996	NALCN Isoform 1 of Sodium leak channel non-selective protein
604	3	20.97	22	ipi.HUMAN	IPI00178116	MBNL1 muscleblind-like 1 isoform e
605	3	20.99	8	ipi.HUMAN	IPI00830067	ARHGAP30 Isoform 1 of Rho GTPase-activating protein 30
606	3	20.99	17	ipi.HUMAN	IPI00057097	DNTTIP1 Terminal deoxynucleotidyltransferase-interacting factor 1

#	Number of Peptides	Spectrum Mill Score	Percent Coverage	Database	Accession Number	Protein Name
607	3	21	3	ipi.HUMAN	IPI00782992	SRRM2 Isoform 1 of Serine/arginine repetitive matrix protein 2
608	3	21.03	6	ipi.HUMAN	IPI00024006	PIK3R4 Phosphoinositide 3-kinase regulatory subunit 4
609	3	21.03	5	ipi.HUMAN	IPI00006610	ZBED4 Zinc finger BED domain-containing protein 4
610	3	21.04	11	ipi.HUMAN	IPI00291175	vinculin isoform VCL
611	3	21.05	1	ipi.HUMAN	IPI00019502	MYH9 Myosin-9
612	3	21.06	10	ipi.HUMAN	IPI00337328	ASTE1 18 kDa protein
613	3	21.06	10	ipi.HUMAN	IPI00478128	GATAD2A 70 kDa protein
614	3	21.1	6	ipi.HUMAN	IPI00872622	ANKS1B Uncharacterized protein ANKS1B
615	3	21.1	13	ipi.HUMAN	IPI00642971	EEF1D eukaryotic translation elongation factor 1 delta isoform 1
616	3	21.11	5	ipi.HUMAN	IPI00792389	ATP2A2 115 kDa protein
617	3	21.11	3	ipi.HUMAN	IPI00306791	SORBS1 Sorbin and SH3 domain-containing protein 1
618	3	21.13	4	ipi.HUMAN	IPI00218539	COL11A1 Isoform B of Collagen alpha-1(XI) chain precursor
619	3	21.13	9	ipi.HUMAN	IPI00335421	FIGNL1 Isoform 1 of Fidgetin-like protein 1
620	3	21.15	3	ipi.HUMAN	IPI00640957	CDC42BPA Isoform 2 of Serine/threonine-protein kinase MRCK alpha
621	3	21.16	15	ipi.HUMAN	IPI00007641	IRAK4 Interleukin-1 receptor-associated kinase 4
622	3	21.17	9	ipi.HUMAN	IPI00011454	GANAB Isoform 2 of Neutral alpha-glucosidase AB precursor
623	3	21.18	7	ipi.HUMAN	IPI00655743	ANKS6 Isoform 4 of Ankyrin repeat and SAM domain-containing protein 6
624	3	21.19	11	ipi.HUMAN	IPI00025311	BCAS1 Isoform 1 of Breast carcinoma-amplified sequence 1
625	3	21.19	8	ipi.HUMAN	IPI00409671	DDX42 Isoform 1 of ATP-dependent RNA helicase DDX42
626	3	21.2	5	ipi.HUMAN	IPI00004388	PTPN21 Tyrosine-protein phosphatase non-receptor type 21
627	3	21.21	16	ipi.HUMAN	IPI00005126	EFNB2 Ephrin-B2 precursor
628	3	21.21	7	ipi.HUMAN	IPI00644408	EPHB2 106 kDa protein

Table S1. Supplemental data for Riley et al., 2011 -- Proteins identified from LC-MS proteomics profiling platform

#	Number of Peptides	Spectrum Mill Score	Percent Coverage	Database	Accession Number	Protein Name
629	3	21.21	2	ipi.HUMAN	IPI00152881	SHROOM3 Protein Shroom3
630	3	21.21	3	ipi.HUMAN	IPI00465378	apolipoprotein A-V precursor
631	2	21.22	13	ipi.HUMAN	IPI00021855	APOC1 Apolipoprotein C-I precursor
632	3	21.24	5	ipi.HUMAN	IPI00007843	MYT1L Isoform 1 of Myelin transcription factor 1-like protein
633	3	21.25	7	ipi.HUMAN	IPI00413780	PKN3 Serine/threonine-protein kinase N3
634	3	21.26	4	ipi.HUMAN	IPI00160290	BCL9 B-cell CLL/lymphoma 9 protein
635	3	21.29	10	ipi.HUMAN	IPI00419847	SORBS3 Isoform Alpha of Vinexin
636	3	21.31	13	ipi.HUMAN	IPI00444138	AFAP1 actin filament associated protein 1
637	3	21.31	3	ipi.HUMAN	IPI00012837	KIF5B Kinesin heavy chain
638	3	21.33	7	ipi.HUMAN	IPI00005793	AP3B2 AP-3 complex subunit beta-2
639	3	21.33	4	ipi.HUMAN	IPI00413385	ITIH5L Inter-alpha-trypsin inhibitor heavy chain H5-like protein precursor
640	3	21.34	4	ipi.HUMAN	IPI00743871	INTS7 Isoform 1 of Integrator complex subunit 7
641	3	21.34	3	ipi.HUMAN	IPI00010800	NES Nestin
642	3	21.36	6	ipi.HUMAN	IPI00784366	AP2B1 Isoform 2 of AP-2 complex subunit beta-1
643	3	21.37	11	ipi.HUMAN	IPI00871397	DDX51 Uncharacterized protein DDX51
644	2	21.37	21	ipi.HUMAN	IPI00253036	CD99 antigen, isoform CRA_d
645	3	21.38	6	ipi.HUMAN	IPI00006680	FAM13A1 Isoform 1 of Protein FAM13A1
646	3	21.39	4	ipi.HUMAN	IPI00022881	CLTCL1 Isoform 1 of Clathrin heavy chain 2
647	3	21.39	6	ipi.HUMAN	IPI00003843	TJP2 Isoform A1 of Tight junction protein ZO-2
648	3	21.4	7	ipi.HUMAN	IPI00293203	SULF1 Extracellular sulfatase Sulf-1 precursor
649	3	21.42	19	ipi.HUMAN	IPI00006662	APOD Apolipoprotein D precursor
650	3	21.42	3	ipi.HUMAN	IPI00815893	CHD2 Isoform 1 of Chromodomain-helicase-DNA-binding protein 2
651	3	21.42	11	ipi.HUMAN	IPI00237011	STK38L Serine/threonine-protein kinase 38-like
652	3	21.45	3	ipi.HUMAN	IPI00215615	ATP7A Isoform 2 of Copper-transporting ATPase 1
653	3	21.45	8	ipi.HUMAN	IPI00746452	IMPG1 Interphotoreceptor matrix proteoglycan 1 precursor
654	3	21.47	5	ipi.HUMAN	IPI00396234	ATP11C Isoform 1 of Probable phospholipid-transporting ATPase IG
655	3	21.47	9	ipi.HUMAN	IPI00147874	NANS Sialic acid synthase

#	Number of Peptides	Spectrum Mill Score	Percent Coverage	Database	Accession Number	Protein Name
656	3	21.47	5	ipi.HUMAN	IPI00026262	RASA1 Isoform 1 of Ras GTPase-activating protein 1
657	3	21.48	2	ipi.HUMAN	IPI00448465	ANKRD12 Isoform 1 of Ankyrin repeat domain-containing protein 12
658	3	21.49	3	ipi.HUMAN	IPI00021493	TNKS Isoform 1 of Tankyrase-1
659	3	21.5	5	ipi.HUMAN	IPI00658047	ADAMTS6 ADAM metallopeptidase with thrombospondin type 1 motif, 6 preproprotein
660	3	21.5	10	ipi.HUMAN	IPI00027178	AP1G2 AP-1 complex subunit gamma-like 2
661	3	21.5	7	ipi.HUMAN	IPI00033019	KCNB1 Potassium voltage-gated channel subfamily B member 1
662	3	21.51	5	ipi.HUMAN	IPI00746412	SFRS2IP splicing factor, arginine/serine-rich 2, interacting protein
663	3	21.52	4	ipi.HUMAN	IPI00807589	COBL Isoform 2 of Protein cordon-bleu
664	3	21.53	3	ipi.HUMAN	IPI00293460	ABCA1 ATP-binding cassette sub-family A member 1
665	3	21.53	7	ipi.HUMAN	IPI00021333	NEK4 Serine/threonine-protein kinase Nek4
666	3	21.53	16	ipi.HUMAN	IPI00009032	SSB Lupus La protein
667	3	21.55	5	ipi.HUMAN	IPI00419992	AASDH Isoform 1 of Acyl-CoA synthetase family member 4
668	4	21.58	8	ipi.HUMAN	IPI00023014	von Willebrand factor
669	3	21.61	14	ipi.HUMAN	IPI00465054	THUMPD1 Putative uncharacterized protein DKFZp686C1054
670	3	21.63	4	ipi.HUMAN	IPI00374520	FAM75A6 Protein FAM75A6
671	4	21.63	2	ipi.HUMAN	IPI00175649	LRRK2 Leucine-rich repeat serine/threonine-protein kinase 2
672	3	21.64	7	ipi.HUMAN	IPI00015952	EIF4G2 Eukaryotic translation initiation factor 4 gamma 2
673	3	21.65	3	ipi.HUMAN	IPI00465045	DIP2B DIP2 disco-interacting protein 2 homolog B
674	3	21.65	3	ipi.HUMAN	IPI00174025	DNMBP Isoform 1 of Dynamin-binding protein
675	3	21.67	7	ipi.HUMAN	IPI00288894	ADAM17 Isoform A of ADAM 17 precursor
676	3	21.67	14	ipi.HUMAN	IPI00303276	IFI44L interferon-induced protein 44-like
677	3	21.67	4	ipi.HUMAN	IPI00444656	RUSC2 Iporin
678	3	21.69	5	ipi.HUMAN	IPI00000877	HYOU1 Hypoxia up-regulated protein 1 precursor
679	3	21.7	10	ipi.HUMAN	IPI00796219	KIF17 Kinesin family member 17

#	Number of Peptides	Spectrum Mill Score	Percent Coverage	Database	Accession Number	Protein Name
680	3	21.71	12	ipi.HUMAN	IPI00640364	OTUD5 Isoform 1 of OTU domain-containing protein 5
681	4	21.72	10	ipi.HUMAN	IPI00090327	VPS45 Vacuolar protein sorting-associated protein 45
682	3	21.73	23	ipi.HUMAN	IPI00013508	actinin, alpha 1 isoform b
683	3	21.75	2	ipi.HUMAN	IPI00171716	TTC28 OTTHUMP00000028696
684	4	21.76	5	ipi.HUMAN	IPI00217413	DHX29 Putative ATP-dependent RNA helicase DHX29
685	3	21.76	7	ipi.HUMAN	IPI00412252	RAPGEF5 Uncharacterized protein RAPGEF5
686	3	21.77	7	ipi.HUMAN	IPI00478667	PCDH20 Protocadherin-20 precursor
687	3	21.78	2	ipi.HUMAN	IPI00184772	DOCK6 Dedicator of cytokinesis protein 6
688	4	21.78	8	ipi.HUMAN	IPI00008485	IREB2 Iron-responsive element-binding protein 1
689	3	21.81	3	ipi.HUMAN	IPI00299037	CACNA1G Isoform 5 of Voltage-dependent T-type calcium channel subunit alpha-1G
690	3	21.81	2	ipi.HUMAN	IPI00021175	CRKRS Isoform 1 of Cell division cycle 2-related protein kinase 7
691	3	21.81	5	ipi.HUMAN	IPI00552073	DHX16 DEAH
692	3	21.81	17	ipi.HUMAN	IPI00328094	FOXP3 Isoform 1 of Forkhead box protein P3
693	4	21.81	3	ipi.HUMAN	IPI00873792	MYH11 228 kDa protein
694	3	21.84	5	ipi.HUMAN	IPI00871726	MAP4 128 kDa protein
695	3	21.84	4	ipi.HUMAN	IPI00219563	PLCB1 Isoform A of 1-phosphatidylinositol-4,5-bisphosphate phosphodiesterase beta-1
696	3	21.87	4	ipi.HUMAN	IPI00303152	COL22A1 collagen, type XXII, alpha 1
697	3	21.93	5	ipi.HUMAN	IPI00395692	HS6ST2 heparan sulfate 6-O-sulfotransferase 2 isoform L
698	3	21.93	12	ipi.HUMAN	IPI00021975	TNFRSF10A Tumor necrosis factor receptor superfamily member 10A precursor
699	3	21.94	5	ipi.HUMAN	IPI00396243	WDR19 Isoform 1 of WD repeat-containing protein 19
700	3	21.95	2	ipi.HUMAN	IPI00002355	CABIN1 Calcineurin-binding protein Cabin 1
701	3	21.96	14	ipi.HUMAN	IPI00395601	SYT10 Synaptotagmin-10

Table S1. Supplemental data for Riley et al., 2011 -- Proteins identified from LC-MS proteomics profiling platform

#	Number of Peptides	Spectrum Mill Score	Percent Coverage	Database	Accession Number	Protein Name
702	3	21.98	3	ipi.HUMAN	IPI00292056	PIK3C2B Phosphatidylinositol-4-phosphate 3-kinase C2 domain-containing beta polypeptide
703	3	21.99	3	ipi.HUMAN	IPI00410096	IFT172 selective LIM binding factor homolog
704	3	22	7	ipi.HUMAN	IPI00398765	PDE1C Isoform PDE1C2 of Calcium/calmodulin-dependent 3',5'-cyclic nucleotide phosphodiesterase 1C
705	3	22.01	3	ipi.HUMAN	IPI00307829	CGNL1 Isoform 1 of Cingulin-like protein 1
706	4	22.01	15	ipi.HUMAN	IPI00290566	TCP1 T-complex protein 1 subunit alpha
707	3	22.02	4	ipi.HUMAN	IPI00218919	ATP4A Potassium-transporting ATPase alpha chain 1
708	3	22.03	4	ipi.HUMAN	IPI00845229	DEPDC2 Isoform 2 of DEP domain-containing protein 2
709	3	22.03	6	ipi.HUMAN	IPI00171741	PGBD4 PiggyBac transposable element-derived protein 4
710	3	22.04	5	ipi.HUMAN	IPI00298285	ERBB3 Isoform 1 of Receptor tyrosine-protein kinase erbB-3 precursor
711	3	22.05	5	ipi.HUMAN	IPI00024048	CDH15 Cadherin-15 precursor
712	3	22.05	5	ipi.HUMAN	IPI00398625	HRNR Hornerin
713	3	22.07	9	ipi.HUMAN	IPI00299063	STIM1 Stromal interaction molecule 1 precursor
714	4	22.09	8	ipi.HUMAN	IPI00011938	ADCY6 Isoform 1 of Adenylate cyclase type 6
715	3	22.09	5	ipi.HUMAN	IPI00014977	MCM9 Isoform 1 of DNA replication licensing factor MCM9
716	3	22.09	3	ipi.HUMAN	IPI00218823	MLL4 Isoform 1 of WW domain-binding protein 7
717	3	22.09	7	ipi.HUMAN	IPI00061007	VWCE von Willebrand factor C and EGF domains
718	3	22.1	2	ipi.HUMAN	IPI00295004	ALS2 Isoform 1 of Alsin
719	3	22.11	9	ipi.HUMAN	IPI00005904	DDX20 Probable ATP-dependent RNA helicase DDX20
720	3	22.11	6	ipi.HUMAN	IPI00181703	MAP3K3 mitogen-activated protein kinase kinase kinase 3 isoform 1
721	3	22.11	7	ipi.HUMAN	IPI00719725	SAPS3 Isoform 5 of SAPS domain family member 3
722	3	22.11	3	ipi.HUMAN	IPI00412399	TRPM2 Isoform 2 of Transient receptor potential cation channel subfamily M member 2
723	3	22.12	23	ipi.HUMAN	IPI00218559	SGCG Gamma-sarcoglycan

#	Number of Peptides	Spectrum Mill Score	Percent Coverage	Database	Accession Number	Protein Name
724	3	22.12	9	ipi.HUMAN	IPI00641980	SOX6 Isoform 1 of Transcription factor SOX-6
725	3	22.15	4	ipi.HUMAN	IPI00396063	DENND4A C-myc promoter-binding protein
726	3	22.16	5	ipi.HUMAN	IPI00031476	PLA2G6 Isoform LH-iPLA2 of 85 kDa calcium-independent phospholipase A2
727	3	22.16	12	ipi.HUMAN	IPI00397579	PPFIA4 Isoform 2 of Liprin-alpha-4
728	3	22.17	10	ipi.HUMAN	IPI00032465	KCNN3 Small-conductance calcium-activated potassium channel SK3
729	3	22.18	2	ipi.HUMAN	IPI00021954	GBF1 Golgi-specific brefeldin A-resistance guanine nucleotide exchange factor 1
730	3	22.18	13	ipi.HUMAN	IPI00418563	TMPRSS11A Isoform 1 of Transmembrane protease, serine 11A
731	3	22.2	5	ipi.HUMAN	IPI00647185	CEP170 180 kDa protein
732	3	22.21	3	ipi.HUMAN	IPI00217405	UBR1 Isoform 1 of E3 ubiquitin-protein ligase UBR1
733	3	22.22	15	ipi.HUMAN	IPI00376239	H1FNT H1 histone family member N, testis-specific
734	3	22.23	5	ipi.HUMAN	IPI00293227	NLRC4 Isoform 1 of NLR family CARD domain-containing protein 4
735	3	22.29	5	ipi.HUMAN	IPI00009793	Complement C1r subcomponent
736	3	22.29	11	ipi.HUMAN	IPI00385777	CDNA FLJ35251 fis, clone PROST2003635, weakly similar to MULTIFUNCTIONAL AMINOACYL-TRNA SYNTHETASE
737	3	22.3	5	ipi.HUMAN	IPI00012500	BRIP1 Isoform 1 of Fanconi anemia group J protein
738	3	22.3	6	ipi.HUMAN	IPI00873717	KCNH7 135 kDa protein
739	3	22.3	15	ipi.HUMAN	IPI00844281	PRAMEF14 Uncharacterized protein PRAMEF14
740	3	22.32	1	ipi.HUMAN	IPI00009329	UTRN Utrophin
741	3	22.38	8	ipi.HUMAN	IPI00000057	COG2 Conserved oligomeric Golgi complex component 2
742	3	22.38	8	ipi.HUMAN	IPI00414008	LMOD1 70 kDa protein
743	4	22.38	10	ipi.HUMAN	IPI00784808	SEPT9 Isoform 5 of Septin-9
744	3	22.39	3	ipi.HUMAN	IPI00157513	ADAMTSL1 ADAMTS-like 1 isoform 4 precursor
745	3	22.39	42	ipi.HUMAN	IPI00794649	ARHGEF3 11 kDa protein
746	4	22.4	18	ipi.HUMAN	IPI00471941	ANXA8
747	3	22.44	12	ipi.HUMAN	IPI00549434	SEPT8 Isoform 1 of Septin-8

#	Number of Peptides	Spectrum Mill Score	Percent Coverage	Database	Accession Number	Protein Name
748	3	22.45	7	ipi.HUMAN	IPI00006671	ZBTB5 Zinc finger and BTB domain-containing protein 5
749	4	22.46	6	ipi.HUMAN	IPI00014843	LRRC16 Leucine-rich repeat-containing protein 16
750	4	22.49	9	ipi.HUMAN	IPI00397836	GSG2 Isoform 1 of Serine/threonine-protein kinase haspin
751	3	22.53	9	ipi.HUMAN	IPI00301277	Heat shock 70 kDa protein L1
752	3	22.53	11	ipi.HUMAN	IPI00796513	IKZF2 60 kDa protein
753	3	22.58	4	ipi.HUMAN	IPI00328753	KTN1 Isoform 1 of Kinectin
754	3	22.59	11	ipi.HUMAN	IPI00024709	IKBKB Inhibitor of nuclear factor kappa-B kinase subunit beta
755	3	22.59	9	ipi.HUMAN	IPI00013871	RRM1 Ribonucleoside-diphosphate reductase large subunit
756	3	22.61	1	ipi.HUMAN	IPI00005826	HERC2 Probable E3 ubiquitin-protein ligase HERC2
757	3	22.61	3	ipi.HUMAN	IPI00328905	IQGAP3 Ras GTPase-activating-like protein IQGAP3
758	4	22.61	6	ipi.HUMAN	IPI00418316	ZMYND8 RACK7 isoform e
759	3	22.63	6	ipi.HUMAN	IPI00306794	CARD6 Caspase recruitment domain-containing protein 6
760	3	22.64	15	ipi.HUMAN	IPI00736030	LOC390427 similar to TBP-associated factor 15 isoform 1
761	3	22.66	4	ipi.HUMAN	IPI00031960	POLR1A polymerase (RNA) I polypeptide A, 194kDa
762	3	22.68	5	ipi.HUMAN	IPI00017304	NOS2A Isoform 1 of Nitric oxide synthase, inducible
763	3	22.68	16	ipi.HUMAN	IPI00514688	RABEPK Rab9 effector protein with kelch motifs
764	3	22.7	8	ipi.HUMAN	IPI00298070	PDE4C Isoform PDE4C1 of cAMP-specific 3',5'-cyclic phosphodiesterase 4C
765	3	22.71	6	ipi.HUMAN	IPI00296563	GUF1 GTP-binding protein GUF1 homolog
766	3	22.75	22	ipi.HUMAN	IPI00514032	CCT3 Chaperonin containing TCP1, subunit 3
767	3	22.76	5	ipi.HUMAN	IPI00477759	INTS2 Integrator complex subunit 2
768	3	22.77	16	ipi.HUMAN	IPI00220563	FOXN3 Isoform 2 of Forkhead box protein N3
769	3	22.78	7	ipi.HUMAN	IPI00002590	DGKB Isoform 1 of Diacylglycerol kinase beta
770	3	22.78	10	ipi.HUMAN	IPI00014307	MTMR2 Myotubularin-related protein 2
771	4	22.81	10	ipi.HUMAN	IPI00789002	AP1GBP1 78 kDa protein
772	3	22.81	10	ipi.HUMAN	IPI00290368	HDC Histidine decarboxylase

#	Number of Peptides	Spectrum Mill Score	Percent Coverage	Database	Accession Number	Protein Name
773	3	22.81	4	ipi.HUMAN	IPI00306984	KCNH8 Potassium voltage-gated channel subfamily H member 8
774	3	22.81	4	ipi.HUMAN	IPI00297617	PHLPP PH domain leucine-rich repeat-containing protein phosphatase
775	3	22.83	10	ipi.HUMAN	IPI00028491	AGPAT5 1-acyl-sn-glycerol-3-phosphate acyltransferase epsilon
776	3	22.84	5	ipi.HUMAN	IPI00384471	BRD1 BRD1 protein
777	4	22.87	16	ipi.HUMAN	IPI00013418	BIRC2 Baculoviral IAP repeat-containing protein 2
778	3	22.91	10	ipi.HUMAN	IPI00852925	ADHFE1 alcohol dehydrogenase, iron containing, 1
779	3	22.92	3	ipi.HUMAN	IPI00413362	SIPA1L1 Isoform 1 of Signal-induced proliferation-associated 1-like protein 1
780	3	22.93	37	ipi.HUMAN	IPI00000350	FAM119B Isoform 2 of Protein FAM119B
781	4	22.94	3	ipi.HUMAN	IPI00868928	KIF26B Isoform 1 of Kinesin-like protein KIF26B
782	3	22.94	13	ipi.HUMAN	IPI00646541	TMEM200A UPF0455 protein KIAA1913
783	3	22.97	6	ipi.HUMAN	IPI00455137	OTUD7A Isoform 2 of OTU domain-containing protein 7A
784	4	22.97	2	ipi.HUMAN	IPI00010369	TEX15 Testis-expressed sequence 15 protein
785	3	22.98	16	ipi.HUMAN	IPI00290928	GNA13 Guanine nucleotide-binding protein alpha-13 subunit
786	3	22.98	10	ipi.HUMAN	IPI00300789	STAU2 Isoform 1 of Double-stranded RNA-binding protein Staufen homolog 2
787	3	22.99	5	ipi.HUMAN	IPI00748953	SAP130 Isoform 1 of Histone deacetylase complex subunit SAP130
788	3	23	10	ipi.HUMAN	IPI00180764	MYST2 Histone acetyltransferase MYST2
789	4	23.01	3	ipi.HUMAN	IPI00160622	CEP250 Isoform 1 of Centrosome-associated protein CEP250
790	4	23.04	6	ipi.HUMAN	IPI00221235	NUP160 nucleoporin 160kDa
791	4	23.06	1	ipi.HUMAN	IPI00329784	RYR3 Isoform 1 of Ryanodine receptor 3
792	3	23.07	4	ipi.HUMAN	IPI00549205	RAD50 Isoform 2 of DNA repair protein RAD50
793	3	23.08	8	ipi.HUMAN	IPI00003166	PLA2G4C Cytosolic phospholipase A2 gamma precursor
794	3	23.13	11	ipi.HUMAN	IPI00796855	IFT81 73 kDa protein

#	Number of Peptides	Spectrum Mill Score	Percent Coverage	Database	Accession Number	Protein Name
795	3	23.13	12	ipi.HUMAN	IPI00029773	REL C-Rel proto-oncogene protein
796	3	23.16	6	ipi.HUMAN	IPI00295401	MAP3K10 Mitogen-activated protein kinase kinase kinase 10
797	3	23.19	16	ipi.HUMAN	IPI00018099	FANCL E3 ubiquitin-protein ligase FANCL
798	4	23.21	9	ipi.HUMAN	IPI00186290	EEF2 Elongation factor 2
799	3	23.21	13	ipi.HUMAN	IPI00157414	ENPP6 Ectonucleotide pyrophosphatase/phosphodiesterase family member 6 precursor
800	3	23.21	13	ipi.HUMAN	IPI00514274	SPAG4L Uncharacterized protein SPAG4L
801	3	23.22	2	ipi.HUMAN	IPI00646645	SETX Isoform 4 of Probable helicase senataxin
802	4	23.27	3	ipi.HUMAN	IPI00883989	FER1L5 fer-1-like 5 isoform 2
803	3	23.29	6	ipi.HUMAN	IPI00304379	USP1 Ubiquitin carboxyl-terminal hydrolase 1
804	3	23.3	7	ipi.HUMAN	IPI00787268	PARP10 Poly [ADP-ribose] polymerase 10
805	4	23.33	6	ipi.HUMAN	IPI00853219	RAPGEF2 Rap guanine nucleotide exchange factor 2
806	3	23.34	10	ipi.HUMAN	IPI00871890	PICALM Uncharacterized protein PICALM
807	3	23.34	4	ipi.HUMAN	IPI00647655	POLA1 DNA polymerase
808	3	23.35	6	ipi.HUMAN	IPI00795553	ARMC8 Isoform 1 of Armadillo repeat-containing protein 8
809	3	23.36	3	ipi.HUMAN	IPI00329707	BCORL1 Isoform 3 of BCoR-like protein 1
810	3	23.36	13	ipi.HUMAN	IPI00414328	BPIL3 Bactericidal/permeability-increasing protein-like 3 precursor
811	3	23.36	10	ipi.HUMAN	IPI00167904	CCDC63 Coiled-coil domain-containing protein 63
812	4	23.4	2	ipi.HUMAN	IPI00218052	WDFY3 Isoform 1 of WD repeat and FYVE domain-containing protein 3
813	3	23.44	13	ipi.HUMAN	IPI00007919	SARM1 Isoform 2 of Sterile alpha and TIR motif-containing protein 1
814	3	23.47	5	ipi.HUMAN	IPI00477535	ERCC5 DNA-repair protein complementing XP-G cells
815	3	23.5	9	ipi.HUMAN	IPI00044761	PUS7 Pseudouridylate synthase 7 homolog
816	3	23.54	4	ipi.HUMAN	IPI00046309	DGKK Diacylglycerol kinase kappa
817	3	23.54	11	ipi.HUMAN	IPI00646510	HCK Isoform p60-HCK of Tyrosine-protein kinase HCK

#	Number of Peptides	Spectrum Mill Score	Percent Coverage	Database	Accession Number	Protein Name
818	3	23.64	8	ipi.HUMAN	IPI00787845	WTIP Wilms tumor 1 interacting protein
819	4	23.69	6	ipi.HUMAN	IPI00797766	M-RIP Protein
820	3	23.71	6	ipi.HUMAN	IPI00465264	NLRX1 Isoform 1 of NLR family member X1
821	3	23.74	7	ipi.HUMAN	IPI00386189	NARG1 Isoform 1 of NMDA receptor-regulated protein 1
822	3	23.74	6	ipi.HUMAN	IPI00304865	TGFBR3 transforming growth factor, beta receptor III
823	4	23.75	11	ipi.HUMAN	IPI00514983	HSPH1 Isoform Alpha of Heat shock protein 105 kDa
824	4	23.75	8	ipi.HUMAN	IPI00175989	MCF2L2 Rho family guanine-nucleotide exchange factor
825	4	23.77	3	ipi.HUMAN	IPI00328195	NBEA Isoform 1 of Protein neurobeachin
826	3	23.78	6	ipi.HUMAN	IPI00294395	C8A Complement component C8 beta chain precursor
827	4	23.79	9	ipi.HUMAN	IPI00843995	AGBL3 Isoform 1 of Cytosolic carboxypeptidase 3
828	3	23.79	5	ipi.HUMAN	IPI00871494	SECISBP2 96 kDa protein
829	3	23.8	8	ipi.HUMAN	IPI00793722	ARHGAP9 Isoform 1 of Rho GTPase-activating protein 9
830	4	23.82	4	ipi.HUMAN	IPI00159049	SETBP1 SET-binding protein
831	3	23.84	4	ipi.HUMAN	IPI00743696	COL4A1 Uncharacterized protein COL4A1
832	3	23.87	10	ipi.HUMAN	IPI00220759	ABCG1 Isoform 3 of ATP-binding cassette sub-family G member 1
833	3	23.88	3	ipi.HUMAN	IPI00025442	PLEKHG4 Isoform 1 of Puratrophin-1
834	4	23.89	4	ipi.HUMAN	IPI00154755	DSCAML1 Down syndrome cell adhesion molecule like 1
835	3	23.9	11	ipi.HUMAN	IPI00656113	SIRPA Signal-regulatory protein alpha
836	4	23.92	5	ipi.HUMAN	IPI00411733	PON1 Serum paraoxonase/arylesterase 1
837	3	23.96	1	ipi.HUMAN	IPI00465428	VPS13C Isoform 1 of Vacuolar protein sorting-associated protein 13C
838	3	23.96	3	ipi.HUMAN	IPI00394789	XXyac-YX155B6.1 Uncharacterized protein ENSP00000358230
839	3	23.99	7	ipi.HUMAN	IPI00007367	MEFV Isoform 1 of Pyrin
840	4	24.01	7	ipi.HUMAN	IPI00020920	ADCY7 Adenylate cyclase type 7
841	3	24.01	20	ipi.HUMAN	IPI00337671	PSEN1 Isoform 5 of Presenilin-1

#	Number of Peptides	Spectrum Mill Score	Percent Coverage	Database	Accession Number	Protein Name
842	3	24.04	6	ipi.HUMAN	IPI00021033	COL3A1 Isoform 1 of Collagen alpha-1(III) chain precursor
843	3	24.07	17	ipi.HUMAN	IPI00418229	C9orf114 HSPC109
844	3	24.08	4	ipi.HUMAN	IPI00796052	CACNA2D2 Isoform 5 of Voltage-dependent calcium channel subunit alpha-2/delta-2 precursor
845	4	24.08	1	ipi.HUMAN	IPI00166612	CMYA5 cardiomyopathy associated 5
846	3	24.09	5	ipi.HUMAN	IPI00303343	SCAF1 Splicing factor, arginine/serine-rich 19
847	3	24.1	1	ipi.HUMAN	IPI00218338	CACNA1E Isoform 2 of Voltage-dependent R-type calcium channel subunit alpha-1E
848	3	24.12	8	ipi.HUMAN	IPI00020672	DPP3
849	4	24.13	3	ipi.HUMAN	IPI00182194	ODZ2 Teneurin-2
850	4	24.14	8	ipi.HUMAN	IPI00027505	ITGAV Isoform 1 of Integrin alpha-V precursor
851	4	24.16	5	ipi.HUMAN	IPI00028493	TSC2 Isoform 1 of Tuberin
852	4	24.19	24	ipi.HUMAN	IPI00292304	C9orf4 Uncharacterized protein C9orf4
853	4	24.19	4	ipi.HUMAN	IPI00013933	DSP Isoform DPI of Desmoplakin
854	3	24.2	1	ipi.HUMAN	IPI00328113	FBN1 Fibrillin-1 precursor
855	4	24.2	10	ipi.HUMAN	IPI00171768	KIF18A Kinesin-like protein KIF18A
856	4	24.2	2	ipi.HUMAN	IPI00020557	LRP1 Prolow-density lipoprotein receptor-related protein 1 precursor
857	4	24.21	4	ipi.HUMAN	IPI00154664	PIWIL2 Piwi-like protein 2
858	3	24.23	7	ipi.HUMAN	IPI00293757	UNC5C Isoform 1 of Netrin receptor UNC5C precursor
859	4	24.25	8	ipi.HUMAN	IPI00061009	WDR67 Isoform 1 of WD repeat-containing protein 67
860	4	24.27	11	ipi.HUMAN	IPI00034308	SARDH Sarcosine dehydrogenase, mitochondrial precursor
861	3	24.27	7	ipi.HUMAN	IPI00791410	TSGA10 testis specific, 10
862	3	24.28	5	ipi.HUMAN	IPI00304187	RBM28 RNA-binding protein 28
863	4	24.31	6	ipi.HUMAN	IPI00056324	HPS3 Isoform 1 of Hermansky-Pudlak syndrome 3 protein
864	4	24.34	16	ipi.HUMAN	IPI00307737	NRG1 Isoform 9 of Pro-neuregulin-1, membrane-bound isoform precursor

Table S1. Supplemental data for Riley et al., 2011 -- Proteins identified from LC-MS proteomics profiling platform

#	Number of Peptides	Spectrum Mill Score	Percent Coverage	Database	Accession Number	Protein Name
865	4	24.35	8	ipi.HUMAN	IPI00552701	DLG3 synapse-associated protein 102 isoform b
866	4	24.41	5	ipi.HUMAN	IPI00657839	C9orf39 Isoform 2 of Uncharacterized protein C9orf39
867	4	24.42	5	ipi.HUMAN	IPI00409582	PLEKHA5 81 kDa protein
868	4	24.43	7	ipi.HUMAN	IPI00001712	CTNNA3 Isoform 1 of Catenin alpha-3
869	3	24.44	4	ipi.HUMAN	IPI00479904	COL27A1 Uncharacterized protein COL27A1
870	4	24.44	17	ipi.HUMAN	IPI00009829	CPA3 Mast cell carboxypeptidase A precursor
871	3	24.45	22	ipi.HUMAN	IPI00647244	SPO11 Isoform 1 of Meiotic recombination protein SPO11
872	4	24.45	7	ipi.HUMAN	IPI00008200	YEATS2 Isoform 1 of YEATS domain-containing protein 2
873	3	24.48	3	ipi.HUMAN	IPI00024853	PRX Isoform 1 of Periaxin
874	3	24.49	11	ipi.HUMAN	IPI00299568	CYP2A6 Cytochrome P450 2A6
875	4	24.5	4	ipi.HUMAN	IPI00009342	IQGAP1 Ras GTPase-activating-like protein IQGAP1
876	4	24.55	9	ipi.HUMAN	IPI00178568	CCDC66 CCDC66 protein
877	3	24.55	5	ipi.HUMAN	IPI00152527	HEL308 DNA helicase HEL308
878	3	24.56	5	ipi.HUMAN	IPI00013721	PRPF4B 43 kDa protein
879	3	24.58	5	ipi.HUMAN	IPI00440727	BRD4 Isoform 1 of Bromodomain-containing protein 4
880	3	24.63	15	ipi.HUMAN	IPI00878993	ZRANB3 49 kDa protein
881	4	24.65	4	ipi.HUMAN	IPI00411452	DOCK11 Uncharacterized protein DOCK11
882	3	24.65	3	ipi.HUMAN	IPI00400922	PDCD11 Protein RRP5 homolog
883	3	24.65	8	ipi.HUMAN	IPI00873445	PROS1 80 kDa protein
884	4	24.65	4	ipi.HUMAN	IPI00794521	WDR62 WD repeat domain 62 isoform 1
885	3	24.7	3	ipi.HUMAN	IPI00298281	LAMC1 Laminin subunit gamma-1 precursor
886	4	24.73	8	ipi.HUMAN	IPI00100151	XRN2 Isoform 1 of 5'-3' exoribonuclease 2
887	3	24.74	5	ipi.HUMAN	IPI00444572	SIPA1L2 Isoform 1 of Signal-induced proliferation-associated 1-like protein 2
888	4	24.8	4	ipi.HUMAN	IPI00654703	SBF2 Myotubularin-related protein 13
889	4	24.8	2	ipi.HUMAN	IPI00398505	USP24 Ubiquitin carboxyl-terminal hydrolase 24
890	4	24.81	3	ipi.HUMAN	IPI00002335	HTT Huntingtin
891	4	24.82	10	ipi.HUMAN	IPI00414320	ANXA11 Annexin A11

#	Number of Peptides	Spectrum Mill Score	Percent Coverage	Database	Accession Number	Protein Name
892	4	24.87	4	ipi.HUMAN	IPI00513791	DOCK7 Isoform 1 of Dedicator of cytokinesis protein 7
893	3	24.87	11	ipi.HUMAN	IPI00424183	SMOX Spermine oxidase isoform 5
894	4	24.89	7	ipi.HUMAN	IPI00042932	ABCB4 Isoform 1 of Multidrug resistance protein 3
895	4	24.9	11	ipi.HUMAN	IPI00301139	MED17 Isoform 1 of Mediator of RNA polymerase II transcription subunit 17
896	4	24.94	6	ipi.HUMAN	IPI00873071	PLCH2 Isoform 2 of 1-phosphatidylinositol-4,5-bisphosphate phosphodiesterase eta-2
897	3	25.02	5	ipi.HUMAN	IPI00414249	NRXN3 Isoform 1 of Neurexin-3-alpha precursor
898	4	25.09	7	ipi.HUMAN	IPI00167804	OSBPL6 oxysterol-binding protein-like protein 6 isoform b
899	4	25.19	3	ipi.HUMAN	IPI00420014	ASCC3L1 Isoform 1 of U5 small nuclear ribonucleoprotein 200 kDa helicase
900	3	25.19	11	ipi.HUMAN	IPI00008575	KHDRBS1 Isoform 1 of KH domain-containing, RNA-binding, signal transduction-associated protein 1
901	3	25.2	28	ipi.HUMAN	IPI00151444	PPAPDC1A Isoform 1 of Phosphatidic acid phosphatase type 2 domain-containing protein 1A
902	4	25.21	7	ipi.HUMAN	IPI00328829	ITIH5 inter-alpha trypsin inhibitor heavy chain precursor 5 isoform 1
903	4	25.21	9	ipi.HUMAN	IPI00024658	OTUD7B OTU domain-containing protein 7B
904	3	25.27	12	ipi.HUMAN	IPI00001562	PCYT1B Isoform 2 of Choline-phosphate cytidylyltransferase B
905	3	25.27	3	ipi.HUMAN	IPI00739504	hornerin
906	3	25.36	9	ipi.HUMAN	IPI00011689	EEF2K Elongation factor 2 kinase
907	4	25.4	5	ipi.HUMAN	IPI00299301	DMN Isoform 1 of Desmuslin
908	3	25.4	2	ipi.HUMAN	IPI00419908	GPR179 Uncharacterized protein GPR179
909	4	25.47	7	ipi.HUMAN	IPI00788863	EHMT2 Euchromatic histone-lysine N-methyltransferase 2
910	3	25.49	3	ipi.HUMAN	IPI00479069	HEATR5B Isoform 1 of HEAT repeat-containing protein 5B
911	4	25.56	11	ipi.HUMAN	IPI00642305	MCM8 MCM8 protein
912	4	25.71	7	ipi.HUMAN	IPI00746807	BAI2 brain-specific angiogenesis inhibitor 2

#	Number of Peptides	Spectrum Mill Score	Percent Coverage	Database	Accession Number	Protein Name
913	4	25.71	11	ipi.HUMAN	IPI00385978	CNTROB Isoform 2 of Centrobin
914	3	25.78	17	ipi.HUMAN	IPI00030320	DDX6 Probable ATP-dependent RNA helicase DDX6
915	3	25.79	12	ipi.HUMAN	IPI00556259	PABPC1L Polyadenylate-binding protein 1-like
916	4	25.8	3	ipi.HUMAN	IPI00294653	DOPEY2 Isoform 1 of Protein dopey-2
917	4	25.81	21	ipi.HUMAN	IPI00007127	DPEP2 Isoform 1 of Dipeptidase 2 precursor
918	3	25.85	11	ipi.HUMAN	IPI00739666	tcag7.956 Similar to Splicing factor, arginine/serine-rich, 46kD
919	4	25.91	7	ipi.HUMAN	IPI00444793	CSPP1 centrosome spindle pole associated protein 1 isoform a
920	4	25.92	6	ipi.HUMAN	IPI00746398	DENND4C Isoform 1 of DENN domain-containing protein 4C
921	4	25.95	7	ipi.HUMAN	IPI00002135	TACC3 Transforming acidic coiled-coil-containing protein 3
922	4	25.97	8	ipi.HUMAN	IPI00414612	HKDC1 Isoform 1 of Putative hexokinase HKDC1
923	4	25.97	5	ipi.HUMAN	IPI00759479	IQSEC2 Isoform 1 of IQ motif and Sec7 domain-containing protein 2
924	3	26.01	9	ipi.HUMAN	IPI00017068	GFI1 Zinc finger protein Gfi-1
925	4	26.02	7	ipi.HUMAN	IPI00299402	PC Pyruvate carboxylase, mitochondrial precursor
926	4	26.03	2	ipi.HUMAN	IPI00248651	REV3L Isoform 1 of DNA polymerase zeta catalytic subunit
927	3	26.04	6	ipi.HUMAN	IPI00218422	FGFR2 Isoform 16 of Fibroblast growth factor receptor 2 precursor
928	4	26.04	4	ipi.HUMAN	IPI00027310	MEGF8 Isoform 1 of Multiple epidermal growth factor-like domains 8
929	3	26.08	6	ipi.HUMAN	IPI00873243	YME1L1 89 kDa protein
930	4	26.09	14	ipi.HUMAN	IPI00398700	GNAO1 Guanine nucleotide-binding protein G(o) subunit alpha 2
931	4	26.09	2	ipi.HUMAN	IPI00299512	NF1 Isoform 2 of Neurofibromin
932	3	26.1	6	ipi.HUMAN	IPI00873137	COL1A2 130 kDa protein
933	3	26.11	7	ipi.HUMAN	IPI00788068	ALOX5 Arachidonate 5-lipoxygenase
934	2	26.12	3	ipi.HUMAN	IPI00019580	PLG Plasminogen precursor
935	4	26.12	23	ipi.HUMAN	IPI00793717	TCP10L Protein

#	Number of Peptides	Spectrum Mill Score	Percent Coverage	Database	Accession Number	Protein Name
936	4	26.17	11	ipi.HUMAN	IPI00783897	DNAH12L Isoform 1 of Axonemal dynein heavy chain 12-like protein
937	4	26.22	8	ipi.HUMAN	IPI00328273	ADCY2 Adenylate cyclase type 2
938	4	26.22	4	ipi.HUMAN	IPI00337397	NUP98 Isoform 5 of Nuclear pore complex protein Nup98-Nup96 precursor
939	4	26.23	6	ipi.HUMAN	IPI00304710	SPHKAP SPHKAP protein
940	4	26.3	11	ipi.HUMAN	IPI00017696	C1S Complement C1s subcomponent precursor
941	4	26.31	8	ipi.HUMAN	IPI00396130	SNIP SNAP25-interacting protein
942	4	26.32	6	ipi.HUMAN	IPI00642769	FCHO1 FCH domain only protein 1. Isoform 2
943	4	26.34	5	ipi.HUMAN	IPI00013455	CLIP1 CLIP1 protein
944	3	26.36	3	ipi.HUMAN	IPI00871702	DYSF Dysferlin
945	4	26.38	14	ipi.HUMAN	IPI00401217	REXO1L1 exonuclease GOR
946	3	26.39	7	ipi.HUMAN	IPI00001762	OPHN1 Oligophrenin 1
947	4	26.4	1	ipi.HUMAN	IPI00103397	MUC5AC Mucin 5AC, oligomeric mucus/gel-forming
948	4	26.4	6	ipi.HUMAN	IPI00872370	NEBL 117 kDa protein
949	4	26.42	4	ipi.HUMAN	IPI00409590	LMO7 Isoform 1 of LIM domain only protein 7
950	4	26.42	24	ipi.HUMAN	IPI00872985	NOLA1 26 kDa protein
951	4	26.44	2	ipi.HUMAN	IPI00152542	DMXL2 DmX-like protein 2
952	2	26.5	4	ipi.HUMAN	IPI00292950	SERPIND1 Serpin peptidase inhibitor, clade D (Heparin cofactor), member 1
953	4	26.5	22	ipi.HUMAN	IPI00478033	ST6GALNAC6 Isoform 1 of Alpha-N-acetylgalactosaminide alpha-2,6-sialyltransferase 6
954	3	26.52	3	ipi.HUMAN	IPI00438288	ERBB2IP Isoform 3 of Protein LAP2
955	4	26.58	3	ipi.HUMAN	IPI00744524	WDR87 Isoform 1 of WD repeat-containing protein 87
956	4	26.59	4	ipi.HUMAN	IPI00217442	ANKHD1-EIF4EBP3
957	4	26.64	2	ipi.HUMAN	IPI00848279	CACNA1A calcium channel, alpha 1A subunit isoform 2
958	4	26.66	14	ipi.HUMAN	IPI00007765	HSPA9 Stress-70 protein, mitochondrial precursor
959	4	26.72	2	ipi.HUMAN	IPI00788083	BAHCC1 BAH domain and coiled-coil containing 1
960	4	26.75	3	ipi.HUMAN	IPI00396015	ACACA Isoform 4 of Acetyl-CoA carboxylase 1
961	4	26.76	6	ipi.HUMAN	IPI00873146	COL4A2 168 kDa protein

#	Number of Peptides	Spectrum Mill Score	Percent Coverage	Database	Accession Number	Protein Name
962	4	26.89	7	ipi.HUMAN	IPI00647020	MYOM1 Uncharacterized protein MYOM1
963	3	26.89	24	ipi.HUMAN	IPI00604590	NME2 Nucleoside diphosphate kinase
964	4	26.89	4	ipi.HUMAN	IPI00291755	NUP210 Isoform 1 of Nuclear pore membrane glycoprotein 210 precursor
965	4	26.98	5	ipi.HUMAN	IPI00170706	TMEM2 Transmembrane protein 2
966	4	26.99	2	ipi.HUMAN	IPI00293274	PKHD1 Isoform 1 of Polycystic kidney and hepatic disease 1 precursor
967	4	27.02	4	ipi.HUMAN	IPI00101923	SPG11 Isoform 1 of Spatacsin
968	4	27.08	22	ipi.HUMAN	IPI00027223	IDH1 Isocitrate dehydrogenase [NADP] cytoplasmic
969	3	27.1	2	ipi.HUMAN	IPI00297851	CHD1 chromodomain helicase DNA binding protein 1
970	4	27.11	5	ipi.HUMAN	IPI00009724	EFCAB6 Isoform 1 of EF-hand calcium-binding domain-containing protein 6
971	4	27.14	15	ipi.HUMAN	IPI00253323	ANKRD57 Ankyrin repeat domain-containing protein 57
972	4	27.18	3	ipi.HUMAN	IPI00293200	PARC OTTHUMP00000016423
973	4	27.22	7	ipi.HUMAN	IPI00185036	MYO3A Myosin IIIA
974	4	27.25	1	ipi.HUMAN	IPI00741527	USH2A Isoform 3 of Usherin precursor
975	4	27.28	12	ipi.HUMAN	IPI00337705	STON2 Isoform 2 of Stonin-2
976	4	27.31	6	ipi.HUMAN	IPI00029455	ABCC6 Multidrug resistance-associated protein 6
977	4	27.31	5	ipi.HUMAN	IPI00376344	MYO1B Isoform 1 of Myosin-Ib
978	4	27.31	11	ipi.HUMAN	IPI00412404	SUPV3L1 ATP-dependent RNA helicase SUPV3L1, mitochondrial precursor
979	4	27.33	8	ipi.HUMAN	IPI00394879	LRRC9 Leucine-rich repeat-containing protein 9
980	4	27.34	11	ipi.HUMAN	IPI00221226	ANXA6 Annexin A6
981	4	27.36	6	ipi.HUMAN	IPI00472302	BCR Isoform 2 of Breakpoint cluster region protein
982	3	27.38	21	ipi.HUMAN	IPI00065430	MAGEB10 Melanoma-associated antigen B10
983	4	27.41	3	ipi.HUMAN	IPI00015346	CELSR2 Cadherin EGF LAG seven-pass G-type receptor 2 precursor
984	3	27.41	3	ipi.HUMAN	IPI00217407	UBR2 Isoform 4 of E3 ubiquitin-protein ligase UBR2
985	4	27.43	7	ipi.HUMAN	IPI00789188	SCUBE2 Isoform 2 of Signal peptide, CUB and EGF-like domain-containing protein 2 precursor

#	Number of Peptides	Spectrum Mill Score	Percent Coverage	Database	Accession Number	Protein Name
986	4	27.44	5	ipi.HUMAN	IPI00396634	CTA-221G9.4 Isoform 1 of Uncharacterized protein KIAA1671
987	4	27.47	7	ipi.HUMAN	IPI00554436	SFRS14 94 kDa protein
988	2	27.54	23	ipi.HUMAN	IPI00021856	APOC2 Apolipoprotein C-II precursor
989	4	27.56	7	ipi.HUMAN	IPI00173946	TANC1 tetratricopeptide repeat, ankyrin repeat and coiled-coil containing 1
990	4	27.58	6	ipi.HUMAN	IPI00792312	INTS3 Isoform 1 of Integrator complex subunit 3
991	4	27.62	7	ipi.HUMAN	IPI00556393	FGFR4 Fibroblast growth factor receptor 4 precursor
992	3	27.64	3	ipi.HUMAN	IPI00872594	CC2D2A coiled-coil and C2 domain containing 2A
993	4	27.68	3	ipi.HUMAN	IPI00022200	COL6A3 alpha 3 type VI collagen isoform 1 precursor
994	4	27.7	27	ipi.HUMAN	IPI00063121	ATPBD4 ATP-binding domain-containing protein 4
995	4	27.7	7	ipi.HUMAN	IPI00644079	HNRNPU heterogeneous nuclear ribonucleoprotein U isoform a
996	4	27.71	8	ipi.HUMAN	IPI00385743	NLRC3 Isoform 2 of Protein NLRC3
997	4	27.78	6	ipi.HUMAN	IPI00097494	PITPNM2 Isoform 1 of Membrane-associated phosphatidylinositol transfer protein 2
998	4	27.8	5	ipi.HUMAN	IPI00426066	DEPDC5 Pleckstrin/ G-protein, interacting region domain containing protein
999	4	27.84	2	ipi.HUMAN	IPI00296365	CENPE Isoform 1 of Centromeric protein E
1000	4	27.84	17	ipi.HUMAN	IPI00019450	TROVE2 Isoform Long of 60 kDa SS-A/Ro ribonucleoprotein
1001	4	27.86	4	ipi.HUMAN	IPI00070943	PI4KA Isoform 1 of Phosphatidylinositol 4-kinase alpha
1002	4	27.87	9	ipi.HUMAN	IPI00290039	CDCP1 Isoform 1 of CUB domain-containing protein 1 precursor
1003	4	27.87	4	ipi.HUMAN	IPI00514071	MAST2 Isoform 1 of Microtubule-associated serine/threonine-protein kinase 2
1004	3	27.87	24	ipi.HUMAN	IPI00022420	RBP4 Plasma retinol-binding protein precursor
1005	4	27.88	7	ipi.HUMAN	IPI00219597	ADAMTS14 ADAM metallopeptidase with thrombospondin type 1 motif, 14 isoform 1 preproprotein
1006	4	27.9	6	ipi.HUMAN	IPI00339214	ADCY5 Adenylate cyclase type 5

#	Number of Peptides	Spectrum Mill Score	Percent Coverage	Database	Accession Number	Protein Name
1007	4	27.9	3	ipi.HUMAN	IPI00152535	CHD5 Chromodomain-helicase-DNA-binding protein 5
1008	4	27.9	6	ipi.HUMAN	IPI00023756	JARID2 Protein Jumonji
1009	4	27.91	9	ipi.HUMAN	IPI00163565	AFAP1L1 Isoform 1 of Actin filament-associated protein 1-like 1
1010	4	27.91	7	ipi.HUMAN	IPI00291868	FBXO41 F-box protein 41
1011	5	28.04	9	ipi.HUMAN	IPI00027721	PDGFRA Isoform 1 of Alpha-type platelet-derived growth factor receptor precursor
1012	4	28.05	5	ipi.HUMAN	IPI00783833	MED12L Isoform 1 of Mediator of RNA polymerase II transcription subunit 12-like protein
1013	4	28.1	2	ipi.HUMAN	IPI00004970	UTP20 Small subunit processome component 20 homolog
1014	4	28.14	4	ipi.HUMAN	IPI00216354	CACNA1D Isoform Beta-cell-type of Voltage-dependent L-type calcium channel subunit alpha-1D
1015	4	28.15	3	ipi.HUMAN	IPI00792788	DOCK5 Isoform 1 of Dedicator of cytokinesis protein 5
1016	4	28.19	12	ipi.HUMAN	IPI00012857	KCNQ3 Potassium voltage-gated channel subfamily KQT member 3
1017	4	28.19	5	ipi.HUMAN	IPI00641172	LAMC3 Laminin subunit gamma-3 precursor
1018	5	28.31	14	ipi.HUMAN	IPI00019088	COL9A2 Collagen alpha-2(IX) chain precursor
1019	4	28.31	4	ipi.HUMAN	IPI00024279	HEATR1 HEAT repeat-containing protein 1
1020	4	28.31	6	ipi.HUMAN	IPI00852862	PCDH15 Protocadherin 15
1021	4	28.31	3	ipi.HUMAN	IPI00006714	PTPN13 Isoform 3 of Tyrosine-protein phosphatase non-receptor type 13
1022	4	28.37	2	ipi.HUMAN	IPI00298306	ATM Serine-protein kinase ATM
1023	4	28.39	8	ipi.HUMAN	IPI00012989	MAN2B1 Lysosomal alpha-mannosidase precursor
1024	4	28.43	3	ipi.HUMAN	IPI00307259	DNAJC13 DnaJ homolog subfamily C member 13
1025	4	28.46	2	ipi.HUMAN	IPI00794880	CHD7 Isoform 1 of Chromodomain-helicase-DNA-binding protein 7
1026	4	28.46	4	ipi.HUMAN	IPI00004237	PHKA2 Phosphorylase b kinase regulatory subunit alpha, liver isoform

Table S1. Supplemental data for Riley et al., 2011 -- Proteins identified from LC-MS proteomics profiling platform

#	Number of Peptides	Spectrum Mill Score	Percent Coverage	Database	Accession Number	Protein Name
1027	4	28.66	10	ipi.HUMAN	IPI00219647	PCDHAC1 Isoform Short of Protocadherin alpha C1 precursor
1028	4	28.68	4	ipi.HUMAN	IPI00152639	ADAMTS19 ADAMTS-19 precursor
1029	4	28.68	6	ipi.HUMAN	IPI00419100	FGD6 Isoform 1 of FYVE, RhoGEF and PH domain-containing protein 6
1030	4	28.68	8	ipi.HUMAN	IPI00293098	WDR63 WD40-like domain containing protein
1031	5	28.69	9	ipi.HUMAN	IPI00011651	PTPRG Isoform 1 of Receptor-type tyrosine-protein phosphatase gamma precursor
1032	4	28.69	5	ipi.HUMAN	IPI00015180	SHROOM2 Protein Shroom2
1033	5	28.73	1	ipi.HUMAN	IPI00749231	GPR98 G protein-coupled receptor 98 precursor
1034	4	28.75	9	ipi.HUMAN	IPI00329708	SUV420H1 Isoform 1 of Histone-lysine N-methyltransferase SUV420H1
1035	4	28.77	10	ipi.HUMAN	IPI00145593	NOM1 Nucleolar MIF4G domain-containing protein 1
1036	4	28.77	4	ipi.HUMAN	IPI00385511	TNRC6B Uncharacterized protein TNRC6B
1037	4	28.79	6	ipi.HUMAN	IPI00374337	GRID1 Glutamate receptor delta-1 subunit precursor
1038	4	28.88	3	ipi.HUMAN	IPI00220694	CASC5 Isoform 1 of Protein CASC5
1039	4	28.9	18	ipi.HUMAN	IPI00219005	FKBP4 FK506-binding protein 4
1040	5	29.02	9	ipi.HUMAN	IPI00031386	PIK3CA Phosphatidylinositol-4,5-bisphosphate 3-kinase catalytic subunit alpha isoform
1041	4	29.07	1	ipi.HUMAN	IPI00872759	MLL2 564 kDa protein
1042	4	29.12	7	ipi.HUMAN	IPI00400935	COL16A1 Isoform 1 of Collagen alpha-1(XVI) chain precursor
1043	4	29.13	4	ipi.HUMAN	IPI00783559	EXOC2 Exocyst complex component 2
1044	4	29.13	13	ipi.HUMAN	IPI00018090	IFT74 coiled-coil domain containing 2 isoform b
1045	4	29.21	4	ipi.HUMAN	IPI00879253	ZFYVE26 Isoform 1 of Zinc finger FYVE domain-containing protein 26
1046	4	29.23	11	ipi.HUMAN	IPI00795221	PTPN6 protein tyrosine phosphatase, non-receptor type 6 isoform 3
1047	3	29.25	3	ipi.HUMAN	IPI00444012	MYO16 Isoform 1 of Myosin-XVI
1048	5	29.32	6	ipi.HUMAN	IPI00029822	SMARCA4 SMARCA4 isoform 2
1049	4	29.34	2	ipi.HUMAN	IPI00783665	LAMA5 Laminin subunit alpha-5 precursor

#	Number of Peptides	Spectrum Mill Score	Percent Coverage	Database	Accession Number	Protein Name
1050	4	29.42	5	ipi.HUMAN	IPI00218397	SBF1 Isoform 4 of Myotubularin-related protein 5
1051	4	29.46	10	ipi.HUMAN	IPI00294982	ESR1 Isoform Long of Estrogen receptor
1052	4	29.48	20	ipi.HUMAN	IPI00478946	CREB5 cAMP responsive element binding protein 5 isoform alpha
1053	4	29.51	6	ipi.HUMAN	IPI00815713	TCOF1 Isoform 4 of Treacle protein
1054	4	29.54	2	ipi.HUMAN	IPI0010402	SH3 domain binding glutamic acid-rich protein like 3
1055	4	29.56	4	ipi.HUMAN	IPI00871372	HECTD1 HECT domain containing 1
1056	4	29.57	10	ipi.HUMAN	IPI00059279	EXOC4 Exocyst complex component 4
1057	4	29.57	13	ipi.HUMAN	IPI00007983	PDLIM2 Isoform 3 of PDZ and LIM domain protein 2
1058	4	29.65	5	ipi.HUMAN	IPI00031519	DNMT1 Isoform 1 of DNA (cytosine-5)-methyltransferase 1
1059	4	29.74	15	ipi.HUMAN	IPI00009822	SRP54 Signal recognition particle 54 kDa protein
1060	5	29.79	4	ipi.HUMAN	IPI00017891	APC2 Isoform 2 of Adenomatous polyposis coli protein 2
1061	5	29.8	5	ipi.HUMAN	IPI00477611	COL5A1 184 kDa protein
1062	4	29.8	2	ipi.HUMAN	IPI00289831	PTPRS Isoform PTPS of Receptor-type tyrosine-protein phosphatase S precursor
1063	4	29.81	4	ipi.HUMAN	IPI00788666	LOXHD1 Protein
1064	4	29.95	10	ipi.HUMAN	IPI00074258	MASTL Isoform 1 of Microtubule-associated serine/threonine-protein kinase-like
1065	4	30.06	19	ipi.HUMAN	IPI00816347	RGMA Isoform 2 of Repulsive guidance molecule A precursor
1066	4	30.1	6	ipi.HUMAN	IPI00783271	LRPPRC Leucine-rich PPR motif-containing protein, mitochondrial precursor
1067	4	30.11	4	ipi.HUMAN	IPI00872997	KIF21B Isoform 1 of Kinesin-like protein KIF21B
1068	4	30.14	3	ipi.HUMAN	IPI00604711	KIF1A Isoform 1 of Kinesin-like protein KIF1A
1069	4	30.16	4	ipi.HUMAN	IPI00783781	NUP205 Nuclear pore complex protein Nup205
1070	5	30.19	2	ipi.HUMAN	IPI00074962	ANK2 ankyrin 2 isoform 1
1071	4	30.2	9	ipi.HUMAN	IPI00217240	WDR75 WD repeat-containing protein 75
1072	4	30.23	4	ipi.HUMAN	IPI00043407	TNRC6C Isoform 2 of Trinucleotide repeat-containing gene 6C protein

#	Number of Peptides	Spectrum Mill Score	Percent Coverage	Database	Accession Number	Protein Name
1073	5	30.39	3	ipi.HUMAN	IPI00329573	COL12A1 Isoform 1 of Collagen alpha-1(XII) chain precursor
1074	4	30.45	11	ipi.HUMAN	IPI00852984	CGN CGN protein
1075	4	30.45	4	ipi.HUMAN	IPI00240812	PDS5B Isoform 1 of Sister chromatid cohesion protein PDS5 homolog B
1076	4	30.49	9	ipi.HUMAN	IPI00719085	FAM120A Isoform E of UPF0318 protein FAM120A
1077	5	30.55	9	ipi.HUMAN	IPI00456677	FBXO38 Isoform 2 of F-box only protein 38
1078	5	30.7	5	ipi.HUMAN	IPI00297254	SIPA1L3 Signal-induced proliferation-associated 1-like protein 3
1079	4	30.75	5	ipi.HUMAN	IPI00008091	INOC1 Putative DNA helicase INO80 complex homolog 1
1080	4	30.76	7	ipi.HUMAN	IPI00300384	ERBB2 Receptor tyrosine-protein kinase erbB-2 precursor
1081	4	30.77	4	ipi.HUMAN	IPI00419253	NAP5 Isoform 1 of Nck-associated protein 5
1082	4	30.8	8	ipi.HUMAN	IPI00161085	NOL8 Isoform 1 of Nucleolar protein 8
1083	4	30.82	7	ipi.HUMAN	IPI00332155	GPRIN1 Isoform 1 of G protein-regulated inducer of neurite outgrowth 1
1084	5	30.93	16	ipi.HUMAN	IPI00787050	NPTX1 Neuronal pentraxin-1 precursor
1085	5	30.95	10	ipi.HUMAN	IPI00065435	FGD4 Isoform 1 of FYVE, RhoGEF and PH domain-containing protein 4
1086	4	31.09	2	ipi.HUMAN	IPI00788786	VWF 309 kDa protein
1087	5	31.16	3	ipi.HUMAN	IPI00333753	ITPR1 Isoform 1 of Inositol 1,4,5-trisphosphate receptor type 1
1088	5	31.27	5	ipi.HUMAN	IPI00237884	AKAP12 Isoform 1 of A-kinase anchor protein 12
1089	5	31.37	4	ipi.HUMAN	IPI00873065	HIVEP1 Zinc finger protein 40
1090	4	31.43	8	ipi.HUMAN	IPI00385887	Tyrosine kinase
1091	5	31.55	5	ipi.HUMAN	IPI00642716	MYH7B myosin, heavy polypeptide 7B, cardiac muscle, beta
1092	5	31.62	2	ipi.HUMAN	IPI00168806	MLL3 Myeloid/lymphoid or mixed-lineage leukemia 3 isoform 1
1093	5	31.64	2	ipi.HUMAN	IPI00004233	MKI67 Isoform Long of Antigen KI-67
1094	4	31.67	4	ipi.HUMAN	IPI00030542	CTAGE6 CTAGE family, member 6

Table S1. Supplemental data for Riley et al., 2011 -- Proteins identified from LC-MS proteomics profiling platform

#	Number of Peptides	Spectrum Mill Score	Percent Coverage	Database	Accession Number	Protein Name
1095	4	31.75	3	ipi.HUMAN	IPI00026256	FLG Filaggrin
1096	5	31.77	3	ipi.HUMAN	IPI00289334	FLNB Isoform 1 of Filamin-B
1097	5	31.77	5	ipi.HUMAN	IPI00783228	SPTB spectrin beta isoform a
1098	5	31.79	4	ipi.HUMAN	IPI00336047	MYO9B Isoform Long of Myosin-IXb
1099	4	31.84	6	ipi.HUMAN	IPI00002232	TAOK1 Isoform 1 of Serine/threonine-protein kinase TAO1
1100	5	31.86	9	ipi.HUMAN	IPI00027356	ADCY8 Adenylate cyclase type 8
1101	4	31.88	37	ipi.HUMAN	IPI00045939	ADO 2-aminoethanethiol dioxygenase
1102	4	31.95	2	ipi.HUMAN	IPI00782981	ABCA12 Isoform 1 of ATP-binding cassette sub-family A member 12
1103	5	31.96	3	ipi.HUMAN	IPI00827896	MAST4 Uncharacterized protein MAST4
1104	4	32	11	ipi.HUMAN	IPI00783995	EFHB EF hand domain family, member B
1105	5	32.06	4	ipi.HUMAN	IPI00294728	DMXL1 DmX-like protein 1
1106	4	32.19	3	ipi.HUMAN	IPI00784294	DOCK4 DOCK4 protein
1107	5	32.25	4	ipi.HUMAN	IPI00012347	MXRA5 Matrix-remodeling-associated protein 5 precursor
1108	5	32.3	14	ipi.HUMAN	IPI00879473	ACSBG2 Isoform 2 of Long-chain-fatty-acid--CoA ligase ACSBG2
1109	5	32.32	4	ipi.HUMAN	IPI00292771	NUMA1 Isoform 1 of Nuclear mitotic apparatus protein 1
1110	4	32.34	10	ipi.HUMAN	IPI00479837	TMEM16A Isoform 1 of Transmembrane protein 16A
1111	4	32.44	9	ipi.HUMAN	IPI00292671	TOP3B Isoform 1 of DNA topoisomerase 3-beta-1
1112	4	32.47	5	ipi.HUMAN	IPI00218753	TOP2A Isoform 3 of DNA topoisomerase 2-alpha
1113	5	32.5	5	ipi.HUMAN	IPI00871665	PTPRF 212 kDa protein
1114	5	32.58	12	ipi.HUMAN	IPI00157860	AKAP4 Isoform 1 of A-kinase anchor protein 4 precursor
1115	5	32.59	4	ipi.HUMAN	IPI00178352	FLNC Isoform 1 of Filamin-C
1116	4	32.59	4	ipi.HUMAN	IPI00514462	RGAG1 Retrotransposon gag domain-containing protein 1
1117	5	32.67	7	ipi.HUMAN	IPI00873708	INADL 200 kDa protein
1118	5	32.68	8	ipi.HUMAN	IPI00216007	COL4A5 Isoform 2 of Collagen alpha-5(IV) chain precursor

#	Number of Peptides	Spectrum Mill Score	Percent Coverage	Database	Accession Number	Protein Name
1119	5	32.7	5	ipi.HUMAN	IPI00102678	PCNX Isoform 1 of Pecanex-like protein 1
1120	5	32.74	14	ipi.HUMAN	IPI00020366	PHEX Phosphate-regulating neutral endopeptidase
1121	4	32.77	7	ipi.HUMAN	IPI00747180	WDR52 WD repeat protein 52
1122	4	32.79	2	ipi.HUMAN	IPI00328762	ABCA13 Isoform 1 of ATP-binding cassette sub-family A member 13
1123	4	32.82	3	ipi.HUMAN	IPI00144243	HIVEP2 Human immunodeficiency virus type I enhancer-binding protein 2
1124	5	32.89	4	ipi.HUMAN	IPI00180408	MYH15 Myosin-15
1125	5	32.93	4	ipi.HUMAN	IPI00479307	MYH10 Isoform 2 of Myosin-10
1126	5	33.14	8	ipi.HUMAN	IPI00253050	L1TD1 LINE-1 type transposase domain-containing protein 1
1127	5	33.15	4	ipi.HUMAN	IPI00293845	RIF1 Isoform 1 of Telomere-associated protein RIF1
1128	5	33.19	13	ipi.HUMAN	IPI00186946	COL25A1 Uncharacterized protein COL25A1
1129	5	33.26	6	ipi.HUMAN	IPI00016387	PCF11 Pre-mRNA cleavage complex 2 protein Pcf11
1130	5	33.4	2	ipi.HUMAN	IPI00031411	FAT Cadherin-related tumor suppressor homolog precursor
1131	5	33.47	10	ipi.HUMAN	IPI00385322	MYO3B Isoform 5 of Myosin-IIIB
1132	5	33.47	9	ipi.HUMAN	IPI00292934	USP53 Inactive ubiquitin carboxyl-terminal hydrolase 53
1133	5	33.49	2	ipi.HUMAN	IPI00514002	STARD9 Isoform 1 of StAR-related lipid transfer protein 9
1134	5	33.52	5	ipi.HUMAN	IPI00174345	SPAG17 Sperm-associated antigen 17
1135	3	33.57	7	ipi.HUMAN	IPI00022395	C9 Complement component C9 precursor
1136	5	33.59	1	ipi.HUMAN	IPI00152653	DNAH5 Ciliary dynein heavy chain 5
1137	4	33.68	6	ipi.HUMAN	IPI00107472	PTPRU protein tyrosine phosphatase, receptor type, U isoform 3 precursor
1138	5	33.76	6	ipi.HUMAN	IPI00872178	TNN 148 kDa protein
1139	6	33.88	12	ipi.HUMAN	IPI00740211	ARHGAP23 Isoform 2 of Rho GTPase-activating protein 23
1140	5	34.06	1	ipi.HUMAN	IPI00299635	BIRC6 baculoviral IAP repeat-containing 6
1141	5	34.1	4	ipi.HUMAN	IPI00737363	INTS1 DKFZP586J0619 protein
1142	5	34.19	2	ipi.HUMAN	IPI00006091	DMD Isoform 4 of Dystrophin

#	Number of Peptides	Spectrum Mill Score	Percent Coverage	Database	Accession Number	Protein Name
1143	5	34.26	7	ipi.HUMAN	IPI00007921	NRXN2 Isoform 1 of Neurexin-2-alpha precursor
1144	5	34.3	2	ipi.HUMAN	IPI00796450	LYST Isoform 1 of Lysosomal-trafficking regulator
1145	5	34.33	10	ipi.HUMAN	IPI00178302	SEMA6D Isoform 4 of Semaphorin-6D precursor
1146	5	34.44	2	ipi.HUMAN	IPI00740420	PCLO piccolo isoform 1
1147	4	34.59	7	ipi.HUMAN	IPI00019471	IRS1 Insulin receptor substrate 1
1148	4	34.61	4	ipi.HUMAN	IPI00413900	RTEL1 Isoform 1 of Regulator of telomere elongation helicase 1
1149	5	34.67	10	ipi.HUMAN	IPI00456620	BRPF1 bromodomain and PHD finger-containing protein 1 isoform 1
1150	6	34.73	3	ipi.HUMAN	IPI00009286	MLL Isoform 1 of Zinc finger protein HRX
1151	5	34.76	6	ipi.HUMAN	IPI00294603	ZMYM2 Zinc finger, MYM-type 2
1152	4	34.81	3	ipi.HUMAN	IPI00374065	MIA3 Isoform 1 of Melanoma inhibitory activity protein 3 precursor
1153	5	34.85	3	ipi.HUMAN	IPI00064262	DCHS1 Protocadherin-16 precursor
1154	5	34.87	2	ipi.HUMAN	IPI00180384	DNAH7 dynein, axonemal, heavy chain 7
1155	5	34.9	3	ipi.HUMAN	IPI00871932	SPTBN1 276 kDa protein
1156	5	34.98	7	ipi.HUMAN	IPI00004167	SALL1 Sal-like protein 1
1157	5	35.36	4	ipi.HUMAN	IPI00455210	CHD4 Isoform 2 of Chromodomain-helicase-DNA-binding protein 4
1158	5	35.37	2	ipi.HUMAN	IPI00746934	UBR4 Isoform 2 of E3 ubiquitin-protein ligase UBR4
1159	5	35.42	7	ipi.HUMAN	IPI00177884	SYNGAP1 Isoform 1 of Ras GTPase-activating protein SynGAP
1160	4	35.43	7	ipi.HUMAN	IPI00739099	COL5A2 Collagen alpha-2(V) chain precursor
1161	5	35.53	5	ipi.HUMAN	IPI00024163	POLR3A DNA-directed RNA polymerase III subunit RPC1
1162	6	35.83	4	ipi.HUMAN	IPI00012645	SPTBN2 Isoform 1 of Spectrin beta chain, brain 2
1163	5	35.86	17	ipi.HUMAN	IPI00023576	LRRTM2 Leucine-rich repeat transmembrane neuronal protein 2 precursor
1164	5	35.86	5	ipi.HUMAN	IPI00410401	NUP210L Nuclear pore membrane glycoprotein 210-like precursor
1165	5	35.91	4	ipi.HUMAN	IPI00216408	DOCK9 Isoform 1 of Dedicator of cytokinesis protein 9

#	Number of Peptides	Spectrum Mill Score	Percent Coverage	Database	Accession Number	Protein Name
1166	5	35.98	28	ipi.HUMAN	IPI00169383	PGK1 Phosphoglycerate kinase 1
1167	5	36.09	5	ipi.HUMAN	IPI00815851	LRRIQ1 leucine-rich repeats and IQ motif containing 1 isoform 1
1168	4	36.17	8	ipi.HUMAN	IPI00021727	C4BPA C4b-binding protein alpha chain precursor
1169	5	36.29	10	ipi.HUMAN	IPI00302089	CTAGE5 Isoform MEA6 of Cutaneous T-cell lymphoma-associated antigen 5
1170	6	36.42	3	ipi.HUMAN	IPI00376439	VPS13B Isoform 1 of Vacuolar protein sorting-associated protein 13B
1171	5	36.7	18	ipi.HUMAN	IPI00844159	PGM1 65 kDa protein
1172	6	36.92	4	ipi.HUMAN	IPI00307733	SETD2 Isoform 1 of Histone-lysine N-methyltransferase SETD2
1173	6	36.93	5	ipi.HUMAN	IPI00029700	DSCAM Isoform Long of Down syndrome cell adhesion molecule precursor
1174	6	36.97	6	ipi.HUMAN	IPI00328798	BCL9L Isoform 1 of B-cell CLL/lymphoma 9-like protein
1175	5	37.15	19	ipi.HUMAN	IPI00384444	KRT14 Keratin, type I cytoskeletal 14
1176	5	37.17	3	ipi.HUMAN	IPI00412298	ATR Isoform 1 of Serine/threonine-protein kinase ATR
1177	5	37.18	7	ipi.HUMAN	IPI00382589	RREB1 194 kDa protein
1178	5	37.19	14	ipi.HUMAN	IPI00157757	MICAL1 MICAL1 protein
1179	6	37.27	3	ipi.HUMAN	IPI00003384	CELSR1 Isoform 1 of Cadherin EGF LAG seven-pass G-type receptor 1 precursor
1180	5	37.43	4	ipi.HUMAN	IPI00747713	BAZ2B bromodomain adjacent to zinc finger domain, 2B
1181	5	37.65	4	ipi.HUMAN	IPI00007858	MYH13 Myosin-13
1182	6	37.75	9	ipi.HUMAN	IPI00740545	A26C1B Chimeric POTE-actin protein
1183	6	37.87	7	ipi.HUMAN	IPI00295022	NKTR NK-tumor recognition protein
1184	6	38.24	12	ipi.HUMAN	IPI00010196	NRIP1 Nuclear receptor-interacting protein 1
1185	5	38.38	14	ipi.HUMAN	IPI00019943	AFM Afamin precursor
1186	5	38.47	9	ipi.HUMAN	IPI00218648	ARID4A Isoform III of AT-rich interactive domain-containing protein 4A
1187	6	38.48	4	ipi.HUMAN	IPI00787650	ATRX transcriptional regulator ATRX isoform 1

#	Number of Peptides	Spectrum Mill Score	Percent Coverage	Database	Accession Number	Protein Name
1188	6	38.49	2	ipi.HUMAN	IPI00550232	XIRP2 cardiomyopathy associated 3 isoform 1
1189	6	38.53	7	ipi.HUMAN	IPI00219420	SMC3 Structural maintenance of chromosomes protein 3
1190	6	38.61	3	ipi.HUMAN	IPI00377045	LAMA3 Alpha3A
1191	6	38.87	5	ipi.HUMAN	IPI00022449	DOCK2 Isoform 1 of Dedicator of cytokinesis protein 2
1192	6	38.95	4	ipi.HUMAN	IPI00739940	FRYL Isoform 1 of Protein furry homolog-like
1193	6	39.05	4	ipi.HUMAN	IPI00760877	BDP1 transcription factor-like nuclear regulator
1194	6	39.08	9	ipi.HUMAN	IPI00385480	CASKIN1 Caskin-1
1195	6	39.29	4	ipi.HUMAN	IPI00031410	FRAP1 FKBP12-rapamycin complex-associated protein
1196	6	39.3	4	ipi.HUMAN	IPI00455216	CCDC88C Isoform 1 of Protein Daple
1197	6	39.37	6	ipi.HUMAN	IPI00018251	NCOA2 Nuclear receptor coactivator 2
1198	5	39.42	9	ipi.HUMAN	IPI00783004	PLCB4 phospholipase C beta 4 isoform a
1199	6	39.45	4	ipi.HUMAN	IPI00031545	ITPR2 Isoform Long of Inositol 1,4,5-trisphosphate receptor type 2
1200	5	39.48	6	ipi.HUMAN	IPI00294840	AIM1 Absent in melanoma 1 protein
1201	5	39.57	18	ipi.HUMAN	IPI00386418	MYEF2 Isoform 2 of Myelin expression factor 2
1202	6	39.65	6	ipi.HUMAN	IPI00026089	SF3B1 Splicing factor 3B subunit 1
1203	5	39.73	3	ipi.HUMAN	IPI00065931	AKAP13 Isoform 2 of A-kinase anchor protein 13
1204	6	39.85	3	ipi.HUMAN	IPI00020546	ASH1L Probable histone-lysine N-methyltransferase ASH1L
1205	6	39.92	2	ipi.HUMAN	IPI00795015	SACS Isoform 1 of Sacsin
1206	6	40.05	5	ipi.HUMAN	IPI00786960	UNC13C similar to unc-13 homolog C isoform 4
1207	7	40.34	3	ipi.HUMAN	IPI00830074	SPEG SPEG complex locus
1208	6	40.39	3	ipi.HUMAN	IPI00743813	ASPM Isoform 1 of Abnormal spindle-like microcephaly-associated protein
1209	6	40.43	8	ipi.HUMAN	IPI00026219	CPSF1 Cleavage and polyadenylation specificity factor subunit 1
1210	7	40.43	4	ipi.HUMAN	IPI00375294	LAMA1 Laminin subunit alpha-1 precursor
1211	6	40.48	6	ipi.HUMAN	IPI00302329	MYH8 Myosin-8
1212	5	40.54	10	ipi.HUMAN	IPI00030741	LCA5L Uncharacterized protein C21orf13

#	Number of Peptides	Spectrum Mill Score	Percent Coverage	Database	Accession Number	Protein Name
1213	6	40.57	3	ipi.HUMAN	IPI00855998	CENPF Centromere protein F
1214	6	40.6	7	ipi.HUMAN	IPI00175416	PLCH1 Phospholipase C eta 1
1215	6	40.95	9	ipi.HUMAN	IPI00167498	C9orf93 Isoform 2 of Uncharacterized protein C9orf93
1216	6	41.41	9	ipi.HUMAN	IPI00335849	RASAL2 RAS protein activator like 2 isoform 2
1217	6	41.52	10	ipi.HUMAN	IPI00872658	FSIP2 Fibrous sheath-interacting protein 2
1218	6	41.75	10	ipi.HUMAN	IPI00644279	NLRP12 120 kDa protein
1219	5	41.78	3	ipi.HUMAN	IPI00746613	VPS13A Isoform 3 of Vacuolar protein sorting-associated protein 13A
1220	6	42.12	6	ipi.HUMAN	IPI00412650	SVIL Isoform 1 of Supervillin
1221	6	42.31	4	ipi.HUMAN	IPI00002127	DNAH1 Heat shock regulated-1
1222	7	42.33	7	ipi.HUMAN	IPI00619921	DIDO1 Isoform 4 of Death-inducer obliterator 1
1223	6	42.34	18	ipi.HUMAN	IPI00219000	COL8A1 Cell proliferation-inducing protein 41
1224	7	42.37	3	ipi.HUMAN	IPI00219168	SPTBN5 Spectrin beta chain, brain 4
1225	7	42.7	9	ipi.HUMAN	IPI00217259	TEX14 Isoform 1 of Testis-expressed protein 14
1226	6	42.82	8	ipi.HUMAN	IPI00013096	PTPRT Isoform 1 of Receptor-type tyrosine-protein phosphatase T precursor
1227	6	43.36	14	ipi.HUMAN	IPI00440502	BRD2 Isoform 2 of Bromodomain-containing protein 2
1228	6	43.66	12	ipi.HUMAN	IPI00022296	KIT Mast/stem cell growth factor receptor precursor
1229	6	43.71	5	ipi.HUMAN	IPI00645793	TAF1 Isoform 4 of Transcription initiation factor TFIID subunit 1
1230	7	43.73	4	ipi.HUMAN	IPI00297593	USP34 ubiquitin specific protease 34
1231	6	43.78	6	ipi.HUMAN	IPI00797373	DOCK8 Isoform 1 of Dedicator of cytokinesis protein 8
1232	5	43.87	17	ipi.HUMAN	IPI00032220	AGT Angiotensinogen precursor
1233	7	43.95	3	ipi.HUMAN	IPI00412408	BRCA2 Breast cancer type 2 susceptibility protein
1234	7	44.08	9	ipi.HUMAN	IPI00871446	COL4A3 163 kDa protein
1235	6	44.26	2	ipi.HUMAN	IPI00171494	DYNC2H1 dynein, cytoplasmic 2, heavy chain 1
1236	6	44.52	5	ipi.HUMAN	IPI00166010	CNOT1 Isoform 1 of CCR4-NOT transcription complex subunit 1

#	Number of Peptides	Spectrum Mill Score	Percent Coverage	Database	Accession Number	Protein Name
1237	6	44.91	17	ipi.HUMAN	IPI00470885	ARHGAP19 Isoform 2 of Rho GTPase-activating protein 19
1238	4	44.96	3	ipi.HUMAN	IPI00032291	C5 Complement C5 precursor
1239	6	45.12	7	ipi.HUMAN	IPI00439548	BCOR Isoform 1 of BCL-6 corepressor
1240	7	45.65	2	ipi.HUMAN	IPI00167941	MDN1 Midasin
1241	6	45.77	11	ipi.HUMAN	IPI00064158	TTBK1 Isoform 1 of Tau-tubulin kinase 1
1242	6	45.78	10	ipi.HUMAN	IPI00465166	ATP8A2 ATPase, aminophospholipid transporter-like, Class I, type 8A, member 2
1243	6	45.89	7	ipi.HUMAN	IPI00791236	EFCAB5 Isoform 1 of EF-hand calcium-binding domain-containing protein 5
1244	5	46.2	3	ipi.HUMAN	IPI00410351	NCOR1 Isoform 2 of Nuclear receptor corepressor 1
1245	7	46.67	5	ipi.HUMAN	IPI00646366	CEP110 Centrosomal protein 110kDa
1246	5	46.71	52	ipi.HUMAN	IPI00552578	SAA1
1247	6	46.73	3	ipi.HUMAN	IPI00737920	DNAH3 similar to dynein, axonemal, heavy polypeptide 1
1248	7	47.1	13	ipi.HUMAN	IPI00186460	COL2A1 Isoform 1 of Collagen alpha-1(II) chain precursor
1249	7	47.48	3	ipi.HUMAN	IPI00335711	DNAH11 Ciliary dynein heavy chain 11
1250	7	48.9	5	ipi.HUMAN	IPI00745746	PDZD2 Isoform 1 of PDZ domain-containing protein 2
1251	7	48.95	7	ipi.HUMAN	IPI00302328	MYH6 myosin heavy chain 6
1252	8	49.44	2	ipi.HUMAN	IPI00022479	HERC1 guanine nucleotide exchange factor p532
1253	8	50.22	9	ipi.HUMAN	IPI00021753	KIF13B Kinesin-like protein KIF13B
1254	6	50.32	5	ipi.HUMAN	IPI00021048	FER1L3 Isoform 1 of Myoferlin
1255	8	50.84	9	ipi.HUMAN	IPI00167447	FHAD1 Forkhead-associated (FHA) phosphopeptide binding domain 1
1256	6	51.55	8	ipi.HUMAN	IPI00024467	UGCGL2 UDP-glucose:glycoprotein glucosyltransferase 2 precursor
1257	7	51.56	6	ipi.HUMAN	IPI00787327	similar to melanoma associated antigen (mutated) 1-like 1
1258	7	51.68	17	ipi.HUMAN	IPI00759691	BRDT Isoform 2 of Bromodomain testis-specific protein

#	Number of Peptides	Spectrum Mill Score	Percent Coverage	Database	Accession Number	Protein Name
1259	8	51.95	5	ipi.HUMAN	IPI00012391	APC Isoform Long of Adenomatous polyposis coli protein
1260	7	52.17	6	ipi.HUMAN	IPI00641392	JMJD1C jumonji domain containing 1C isoform b
1261	9	52.34	5	ipi.HUMAN	IPI00152462	DNAH3 Ciliary dynein heavy chain 3
1262	7	52.67	12	ipi.HUMAN	IPI00028932	MAST3 Microtubule-associated serine/threonine-protein kinase 3
1263	8	52.68	3	ipi.HUMAN	IPI00218501	RYR2 Isoform 2 of Ryanodine receptor 2
1264	8	52.97	12	ipi.HUMAN	IPI00297646	COL1A1 Collagen alpha-1(I) chain precursor
1265	7	53.25	5	ipi.HUMAN	IPI00001735	NCOR2 nuclear receptor co-repressor 2 isoform 1
1266	9	53.45	6	ipi.HUMAN	IPI00436632	NIPBL Isoform 1 of Nipped-B-like protein
1267	8	53.81	4	ipi.HUMAN	IPI00741005	MGA MAX gene associated
1268	6	54.07	33	ipi.HUMAN	IPI00166729	AZGP1 alpha-2-glycoprotein 1, zinc
1269	4	54.66	14	ipi.HUMAN	IPI00400826	CLU clusterin isoform 1
1270	8	55.37	4	ipi.HUMAN	IPI00302453	DNAH9 Ciliary dynein heavy chain 9
1271	7	55.58	8	ipi.HUMAN	IPI00012269	MMRN1 Multimerin-1 precursor
1272	8	55.59	4	ipi.HUMAN	IPI00045914	SPEN Msx2-interacting protein
1273	8	55.63	4	ipi.HUMAN	IPI00853516	DNAH17 dynein, axonemal, heavy chain 17
1274	7	56.31	21	ipi.HUMAN	IPI00019568	F2 36 kDa protein
1275	7	56.63	5	ipi.HUMAN	IPI00856012	COL6A6 collagen type VI alpha 6
1276	9	56.67	6	ipi.HUMAN	IPI00217930	SON Isoform J of SON protein
1277	9	57.9	6	ipi.HUMAN	IPI00794779	POLQ DNA polymerase theta
1278	9	58.82	4	ipi.HUMAN	IPI00178743	ALMS1 ALMS1
1279	9	59.86	4	ipi.HUMAN	IPI00179298	HUWE1 482 kDa protein
1280	8	60.17	8	ipi.HUMAN	IPI00396577	ARID1B Isoform 3 of AT-rich interactive domain-containing protein 1B
1281	9	60.24	10	ipi.HUMAN	IPI00028448	BAI3 Brain-specific angiogenesis inhibitor 3 precursor
1282	9	60.26	4	ipi.HUMAN	IPI00786995	PRKDC Isoform 1 of DNA-dependent protein kinase catalytic subunit
1283	4	61.05	48	ipi.HUMAN	IPI00853068	HBA2
1284	7	61.53	7	ipi.HUMAN	IPI00306718	SYCP2 Synaptonemal complex protein 2
1285	8	61.58	8	ipi.HUMAN	IPI00333770	DOCK10 dedicator of cytokinesis 10

#	Number of Peptides	Spectrum Mill Score	Percent Coverage	Database	Accession Number	Protein Name
1286	9	61.73	6	ipi.HUMAN	IPI00784201	CEP290 Isoform 1 of Centrosomal protein Cep290
1287	9	62.33	11	ipi.HUMAN	IPI00419345	CCDC18 CCDC18 protein
1288	9	63.78	4	ipi.HUMAN	IPI00856045	AHNAK2 AHNAK nucleoprotein 2
1289	9	63.79	3	ipi.HUMAN	IPI00783637	DNAH2 dynein heavy chain domain 3
1290	10	64.89	4	ipi.HUMAN	IPI00456969	DYNC1H1 Dynein heavy chain, cytosolic
1291	7	65.62	19	ipi.HUMAN	IPI00797833	KNG1 Kininogen 1
1292	10	66.21	2	ipi.HUMAN	IPI00854629	OBSCN Isoform 6 of Obscurin
1293	9	66.75	12	ipi.HUMAN	IPI00787678	ANKRD30A Ankyrin repeat domain 30A
1294	11	68.39	5	ipi.HUMAN	IPI00607852	MYCBP2 Isoform 2 of Probable E3 ubiquitin-protein ligase MYCBP2
1295	6	68.56	16	ipi.HUMAN	IPI00022371	HRG Histidine-rich glycoprotein precursor
1296	10	69.88	5	ipi.HUMAN	IPI00254408	BPTF bromodomain PHD finger transcription factor isoform 1
1297	10	71.22	6	ipi.HUMAN	IPI00879277	TRRAP 436 kDa protein
1298	12	71.68	5	ipi.HUMAN	IPI00784869	DNAH10 dynein, axonemal, heavy chain 10 isoform 1
1299	11	72.1	4	ipi.HUMAN	IPI00642126	RNF213 Isoform 1 of RING finger protein 213
1300	10	72.27	5	ipi.HUMAN	IPI00873991	DNAH8 537 kDa protein
1301	12	74.32	9	ipi.HUMAN	IPI00025418	COL7A1 Isoform 1 of Collagen alpha-1(VII) chain precursor
1302	7	75.09	32	ipi.HUMAN	IPI00029863	SERPINF2 SERPINF2 protein
1303	11	76.15	5	ipi.HUMAN	IPI00783826	RYR1 Isoform 1 of Ryanodine receptor 1
1304	12	76.27	10	ipi.HUMAN	IPI00003515	TRIP11 Thyroid receptor-interacting protein 11
1305	6	78.08	26	ipi.HUMAN	IPI00298828	APOH Beta-2-glycoprotein 1 precursor
1306	12	80.85	3	ipi.HUMAN	IPI00642259	DST Dystonin
1307	6	81.27	32	ipi.HUMAN	IPI00022431	AHSG Alpha-2-HS-glycoprotein precursor
1308	13	82.75	5	ipi.HUMAN	IPI00472779	ANK3 Ankyrin-3
1309	7	84	70	ipi.HUMAN	IPI00654755	HBB Hemoglobin subunit beta
1310	8	86.18	18	ipi.HUMAN	IPI00220327	KRT1 Keratin, type II cytoskeletal 1
1311	13	86.22	1	ipi.HUMAN	IPI00152154	MUC7 Mucin-7
1312	12	87.45	31	ipi.HUMAN	IPI00171903	HNRPM Isoform 1 of Heterogeneous nuclear ribonucleoprotein M

#	*Number of Peptides*	*Spectrum Mill Score*	*Percent Coverage*	*Database*	*Accession Number*	*Protein Name*
1313	13	90.4	5	ipi.HUMAN	IPI00432363	MACF1 Microtubule-actin cross-linking factor 1, isoform 4
1314	7	95.96	21	ipi.HUMAN	IPI00298971	VTN Vitronectin precursor
1315	8	108.24	74	ipi.HUMAN	IPI00021854	APOA2 Apolipoprotein A-II precursor
1316	10	108.82	22	ipi.HUMAN	IPI00019591	CFB B-factor, properdin
1317	13	110.91	42	ipi.HUMAN	IPI00304273	APOA4 Apolipoprotein A-IV precursor
1318	17	116.78	4	ipi.HUMAN	IPI00873123	NEB nebulin
1319	10	121.33	25	ipi.HUMAN	IPI00291866	SERPING1 Plasma protease C1 inhibitor precursor
1320	17	121.86	7	ipi.HUMAN	IPI00021812	AHNAK Neuroblast differentiation-associated protein AHNAK
1321	9	127.09	38	ipi.HUMAN	IPI00022429	ORM1 Alpha-1-acid glycoprotein 1 precursor
1322	12	131.51	14	ipi.HUMAN	IPI00029739	CFH Isoform 1 of Complement factor H precursor
1323	10	131.58	30	ipi.HUMAN	IPI00555812	GC Vitamin D-binding protein precursor
1324	10	135.93	20	ipi.HUMAN	IPI00292530	ITIH1 Inter-alpha-trypsin inhibitor heavy chain H1 precursor
1325	11	136.15	22	ipi.HUMAN	IPI00305461	ITIH2 Inter-alpha-trypsin inhibitor heavy chain H2 precursor
1326	14	141.65	43	ipi.HUMAN	IPI00550991	SERPINA3 Alpha-1-antichymotrypsin precursor
1327	14	159.76	40	ipi.HUMAN	IPI00032179	SERPINC1 Antithrombin III variant
1328	22	161.38	4	ipi.HUMAN	IPI00749005	SYNE1 1011 kDa protein
1329	12	164.55	55	ipi.HUMAN	IPI00785200	IGL@ IGL@ protein
1330	13	181.83	58	ipi.HUMAN	IPI00855916	Transthyretin
1331	13	193.12	42	ipi.HUMAN	IPI00022895	A1BG Alpha-1B-glycoprotein precursor
1332	12	202.25	51	ipi.HUMAN	IPI00761125	IGKC IGKC protein
1333	16	209.4	48	ipi.HUMAN	IPI00022488	HPX Hemopexin precursor
1334	22	222.53	33	ipi.HUMAN	IPI00294193	ITIH4 Isoform 1 of Inter-alpha-trypsin inhibitor heavy chain H4 precursor
1335	20	253.07	37	ipi.HUMAN	IPI00477090	IGHM IGHM protein
1336	21	299.58	53	ipi.HUMAN	IPI00472610	IGHM IGHM protein
1337	48	306.52	2	ipi.HUMAN	IPI00759542	TTN Isoform 8 of Titin
1338	22	329.75	58	ipi.HUMAN	IPI00877703	FGG 52 kDa protein
1339	29	349.07	44	ipi.HUMAN	IPI00017601	CP Ceruloplasmin precursor

#	*Number of Peptides*	*Spectrum Mill Score*	*Percent Coverage*	*Database*	*Accession Number*	*Protein Name*
1340	27	370.16	38	ipi.HUMAN	IPI00021885	FGA Isoform 1 of Fibrinogen alpha chain precursor
1341	33	377.16	26	ipi.HUMAN	IPI00414283	FN1 fibronectin 1 isoform 4 preproprotein
1342	27	394.72	60	ipi.HUMAN	IPI00641737	HP Haptoglobin precursor
1343	28	426.28	60	ipi.HUMAN	IPI00553177	SERPINA1 Isoform 1 of Alpha-1-antitrypsin precursor
1344	30	445.29	61	ipi.HUMAN	IPI00298497	FGB Fibrinogen beta chain precursor
1345	36	512.14	79	ipi.HUMAN	IPI00021841	APOA1 Apolipoprotein A-I precursor
1346	51	618.5	42	ipi.HUMAN	IPI00418163	C4B C4B1
1347	53	753.93	51	ipi.HUMAN	IPI00478003	A2M Alpha-2-macroglobulin precursor
1348	57	761.94	74	ipi.HUMAN	IPI00022463	TF Serotransferrin precursor
1349	75	785.42	23	ipi.HUMAN	IPI00022229	APOB Apolipoprotein B-100 precursor
1350	72	937.83	56	ipi.HUMAN	IPI00783987	complement component 3 precursor
1351	95	1486.14	92	ipi.HUMAN	IPI00745872	ALB Isoform 1 of Serum albumin precursor
